# Vignette-based comparative analysis of ChatGPT and specialist treatment decisions for rheumatic patients: results of the Rheum2Guide study

**DOI:** 10.1007/s00296-024-05675-5

**Published:** 2024-08-10

**Authors:** Hannah Labinsky, Lea-Kristin Nagler, Martin Krusche, Sebastian Griewing, Peer Aries, Anja Kroiß, Patrick-Pascal Strunz, Sebastian Kuhn, Marc Schmalzing, Michael Gernert, Johannes Knitza

**Affiliations:** 1 Department of Internal Medicine 2, Rheumatology/Clinical Immunology, University Hospital Würzburg, Oberdürrbacher Straße 6, 97080 Würzburg, Germany; 2 Division of Rheumatology and Systemic Inflammatory Diseases, III. Department of Medicine, University Medical Center Hamburg-Eppendorf, Hamburg, Germany; 3 Institute for Digital Medicine, University Hospital Giessen-Marburg, Philipps University, Baldingerstrasse, Marburg, Germany; 4 Stanford Center for Biomedical Informatics Research, Stanford University School of Medicine, Palo Alto, CA USA; 5 Department of Rheumatology, Immunologikum, Hamburg, Germany; 6 AGEIS, Université Grenoble Alpes, Grenoble, France

**Keywords:** Artificial intelligence, Large language model, ChatGPT, Clinical decision support system (CDSS)

## Abstract

**Background:**

The complex nature of rheumatic diseases poses considerable challenges for clinicians when developing individualized treatment plans. Large language models (LLMs) such as ChatGPT could enable treatment decision support.

**Objective:**

To compare treatment plans generated by ChatGPT-3.5 and GPT-4 to those of a clinical rheumatology board (RB).

**Design/methods:**

Fictional patient vignettes were created and GPT-3.5, GPT-4, and the RB were queried to provide respective first- and second-line treatment plans with underlying justifications. Four rheumatologists from different centers, blinded to the origin of treatment plans, selected the overall preferred treatment concept and assessed treatment plans’ safety, EULAR guideline adherence, medical adequacy, overall quality, justification of the treatment plans and their completeness as well as patient vignette difficulty using a 5-point Likert scale.

**Results:**

20 fictional vignettes covering various rheumatic diseases and varying difficulty levels were assembled and a total of 160 ratings were assessed. In 68.8% (110/160) of cases, raters preferred the RB’s treatment plans over those generated by GPT-4 (16.3%; 26/160) and GPT-3.5 (15.0%; 24/160). GPT-4’s plans were chosen more frequently for first-line treatments compared to GPT-3.5. No significant safety differences were observed between RB and GPT-4’s first-line treatment plans. Rheumatologists’ plans received significantly higher ratings in guideline adherence, medical appropriateness, completeness and overall quality. Ratings did not correlate with the vignette difficulty. LLM-generated plans were notably longer and more detailed.

**Conclusion:**

GPT-4 and GPT-3.5 generated safe, high-quality treatment plans for rheumatic diseases, demonstrating promise in clinical decision support. Future research should investigate detailed standardized prompts and the impact of LLM usage on clinical decisions.

**Supplementary Information:**

The online version contains supplementary material available at 10.1007/s00296-024-05675-5.

## Introduction:

The various manifestations and associated comorbidities of rheumatic diseases present considerable challenges for pharmacological treatment. When devising individualized treatment plans, clinicians must carefully evaluate an increasing array of options, considering patient-specific factors, safety criteria, and the latest research and guidelines [1]. The European Alliance of Associations for Rheumatology (EULAR) Committees consistently develop and publish treatment recommendations for various rheumatic conditions. The continuous refinement and extension of these EULAR recommendations ensures that practitioners have access to up-to-date and standardized guidance for managing rheumatic diseases and their specific manifestations.

However, aligning personalized treatment decisions with the latest EULAR recommendations and translating them into personalized treatment decisions is becoming more and more challenging. Increasing patient complexity often results in treatment decisions that resemble a trial-and-error approach, underscoring the confusion among rheumatologists and the critical need for decision support [2].

Rapid advancements in artificial intelligence have led to the development of powerful large language models (LLMs). Generative Pre-trained Transformer (GPT), an advanced LLM developed by OpenAI, is one of the most frequently consulted LLMs. Research into the use of GPT in medical decision-making is burgeoning [3, 4]. In rheumatology, GPT has already demonstrated the ability to pass rheumatology exams [5] and even exceeded the diagnostic accuracy of rheumatologists to diagnose inflammatory rheumatologic diseases based on medical history [6]. GPT-4 provided faster, higher quality and even more emphatic answers to frequently asked questions from patients with systemic lupus erythematosus [7]. EULAR acknowledged the potential of big data and called for benchmarking studies [8]. To our knowledge the ability of GPT in generating treatment plans for rheumatic diseases has not yet been investigated and critical scientific evaluations are urgently needed [9]. This study aimed to compare the acceptance, safety, guideline adherence, medical adequacy, overall quality, completeness and justification of first-line and second-line treatment plans generated by GPT-3.5, GPT-4 and rheumatologists for patients with inflammatory rheumatic diseases.

## Methods

### Study flow

Two LLM models (GPT3.5 and 4) and a clinical rheumatology board (RB) at the department of rheumatology at the University Hospital Würzburg were queried to provide a first-line therapy plan, a second-line plan, and justifications for 20 fictional rheumatology patient vignettes. Four experienced blinded rheumatologists from different German rheumatology centers assessed the six different treatment plans for each case. The Philipps-University Marburg Research Ethics Committee confirmed on December 6th 2023 that no ethical approval was required (23–300 ANZ) for this study as only fictional data was used.

### Vignette creation

20 fictional rheumatology patient vignettes were created to encompass various inflammatory rheumatic diseases, including 6 patients with rheumatoid arthritis, 5 patients with spondyloarthritis, 5 patients with mixed-connective tissue disease, and 4 vasculitis patients.

The complexity of these cases varied, incorporating typical and realistic clinical pitfalls that could impact treatment decisions. This diverse selection was made to mirror both common, straightforward rheumatic conditions and rarer diseases for which treatment guidelines are available.

The vignettes were inspired by real patients recently encountered by HL and were subsequently reviewed, edited, and approved by two colleagues to ensure accuracy and relevance.

Each vignette (supplementary Table 1) included:

- Clinical information (age, gender, BMI)
- Current medical history
- Current physical examination findings
- Laboratory chemical and autoimmune serology results
- Radiological and histological findings (when applicable)
- Pulmonary function tests (when applicable)

### Large language model testing

**Fig. 1:**
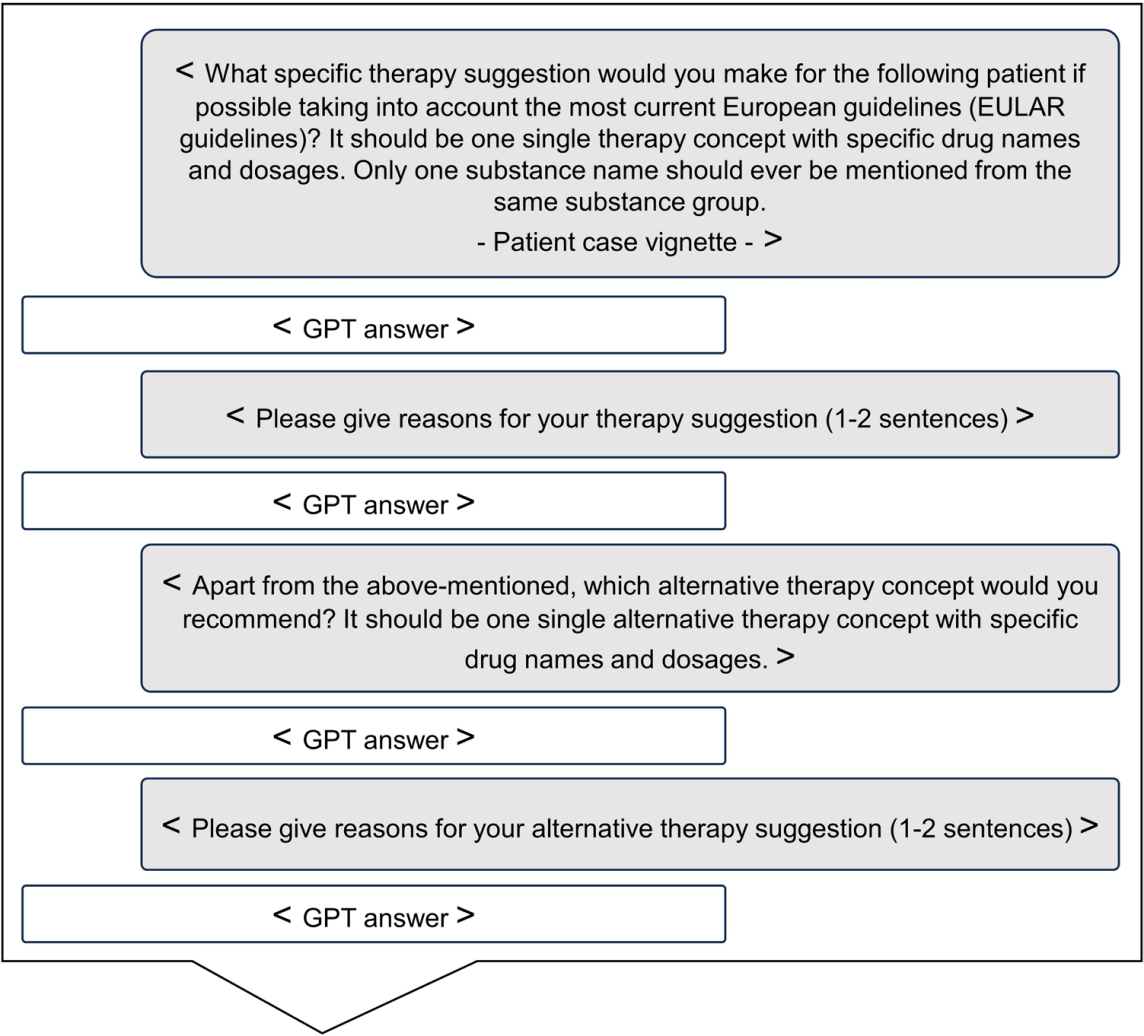
GPT input model

A previously applied[10] standardized prompting strategy was adapted to this study, see Fig. 1. On February 21th 2024, GPT-3.5 and GPT-4 (OpenAI, San Francisco, CA, USA) were tested using the aforementioned 20 vignettes and prompting strategy. Each patient vignette, along with the respective prompts, was entered as a separate, independent chat. The textual inputs included the initial question, followed by the patient case details, and then three follow-up questions. The outputs generated by the models, as well as the responses from the rheumatology board (RB), are provided in the supplementary file 1.

### Comparison of treatment plans

Four blinded senior rheumatologists from four different rheumatology centers assessed the treatment plans. Raters had to select their overall preferred first-line and second-line treatment plan and assess the therapy plans using a 5-point Likert scale (1—strongly disagree, 2—disagree, 3—neither agree nor disagree, 4—agree, 5—strongly agree) based on criteria including treatment plan safety, European treatment guideline (EULAR guideline) adherence, medical adequacy, overall quality, completeness and logic of the treatment plan justification. Both the institutional board and the four raters evaluated the difficulty of the case vignettes on a 5-point Likert scale (1—very easy, 2—easy, 3—moderate, 4—difficult, 5—very difficult). Furthermore, the ratings were assessed for statistically relevant correlation according to the vignette difficulty. Lastly, the character count of the generated treatment plans was compared between the LLMs and RB.

### Statistical analysis

Due to the exploratory character of the trial, no formal sample size calculation was performed. Statistical analysis was performed using Microsoft Excel 2019 and GraphPad Prism 8. The online tool DATAtab (https://datatab.net/statistics-calculator) was used for inter-rater agreement analysis.

Median and range of the data were reported. Statistical differences were assessed by Kruskal–Wallis test with Dunn’s test for multiple comparisons or Spearman correlation analysis (r_s_). P values were reported and P values less than 0.05 were considered significant.

Inter-rater agreement was analyzed by intra-class correlation analysis (metric data) and by Fleiss’ kappa test (categorical data). Intra-class coefficients (ICC) and their 95% confidence intervals (CI) or Fleiss’ kappa coefficients and their 95% CI were reported and interpreted as previously reported [11].

## Results

### Overall therapy concept preference

**Fig. 2:**
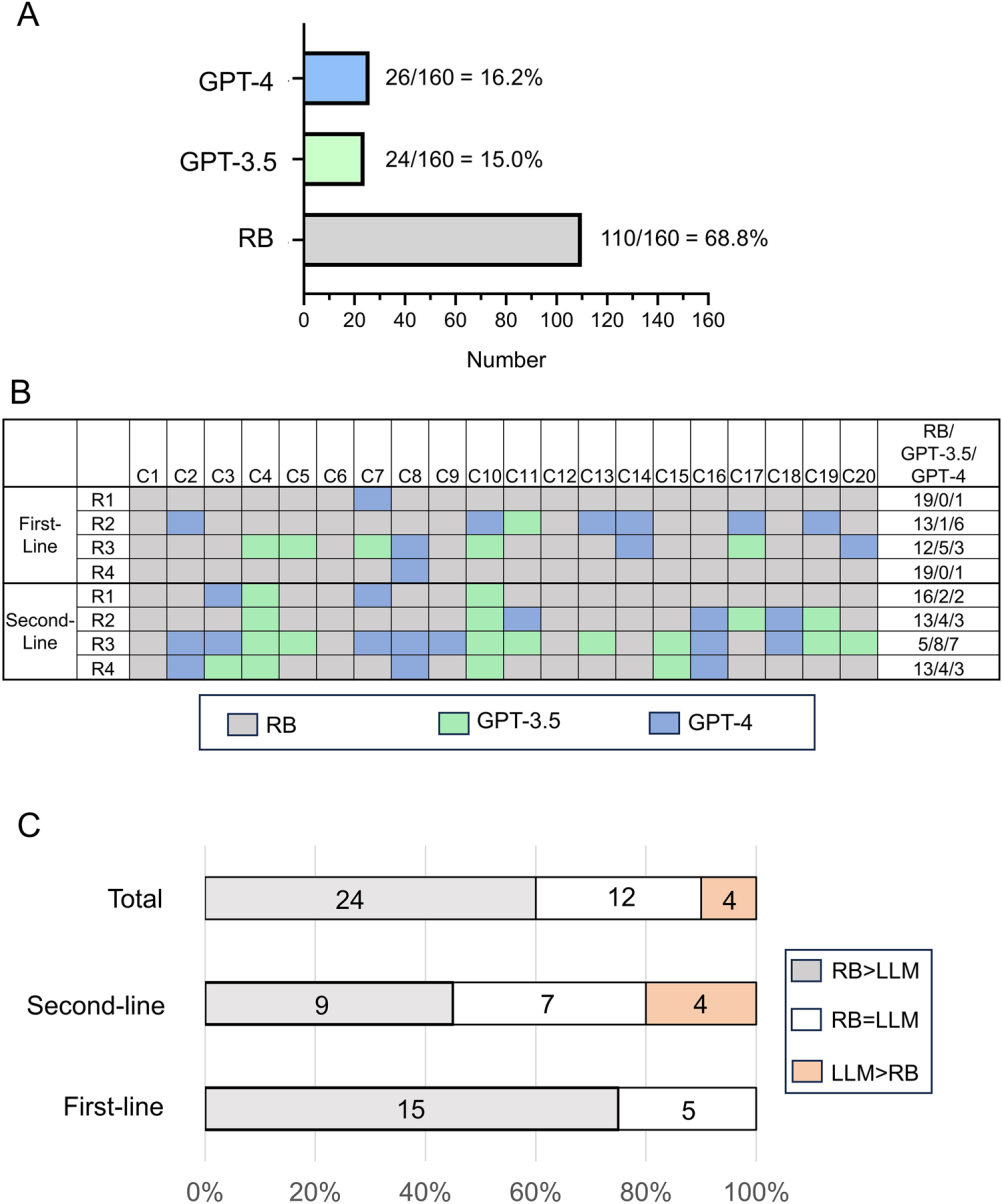
Therapy plan preferences. **A** Total therapy plan preferences are shown in a bar chart. **B** The table displays the various decisions, color-coded for individual case vignettes and raters. **C** The stacked bar charts illustrate the number of case vignettes with a majority favoring (LLM > RB) or opposing (RB > LLM) the LLM (GPT-3.5 and GPT-4), as well as the number of cases resulting in a tie (RB = LLM). The specific counts for each type of decision are indicated. *R1–R4* rater 1–4, *C1–C20* patient case vignette 1–20, *RB* rheumatology board, *LLM* large language model

In the majority of cases, raters preferred the RB treatment plans (110/160 (68.8%)) over concepts generated by GPT-4 (26/160 (16.3%)) and GPT-3.5 (24/160 (15.0%)), see Fig. 2A. Figure 2B displays the treatment preferences according to cases, raters and treatment line. Among the LLM-generated therapy plans, GPT-4 was chosen more frequently for first-line treatments (GPT-3.5: 6/80 (7.5%); GPT-4: 11/80 (13.8%)), whereas GPT-3.5 was preferred more often for second-line treatments (GPT-3.5: 18/80 (22.5%) vs. GPT-4: 15/80 (18.8%)).

**Fig. 3:**
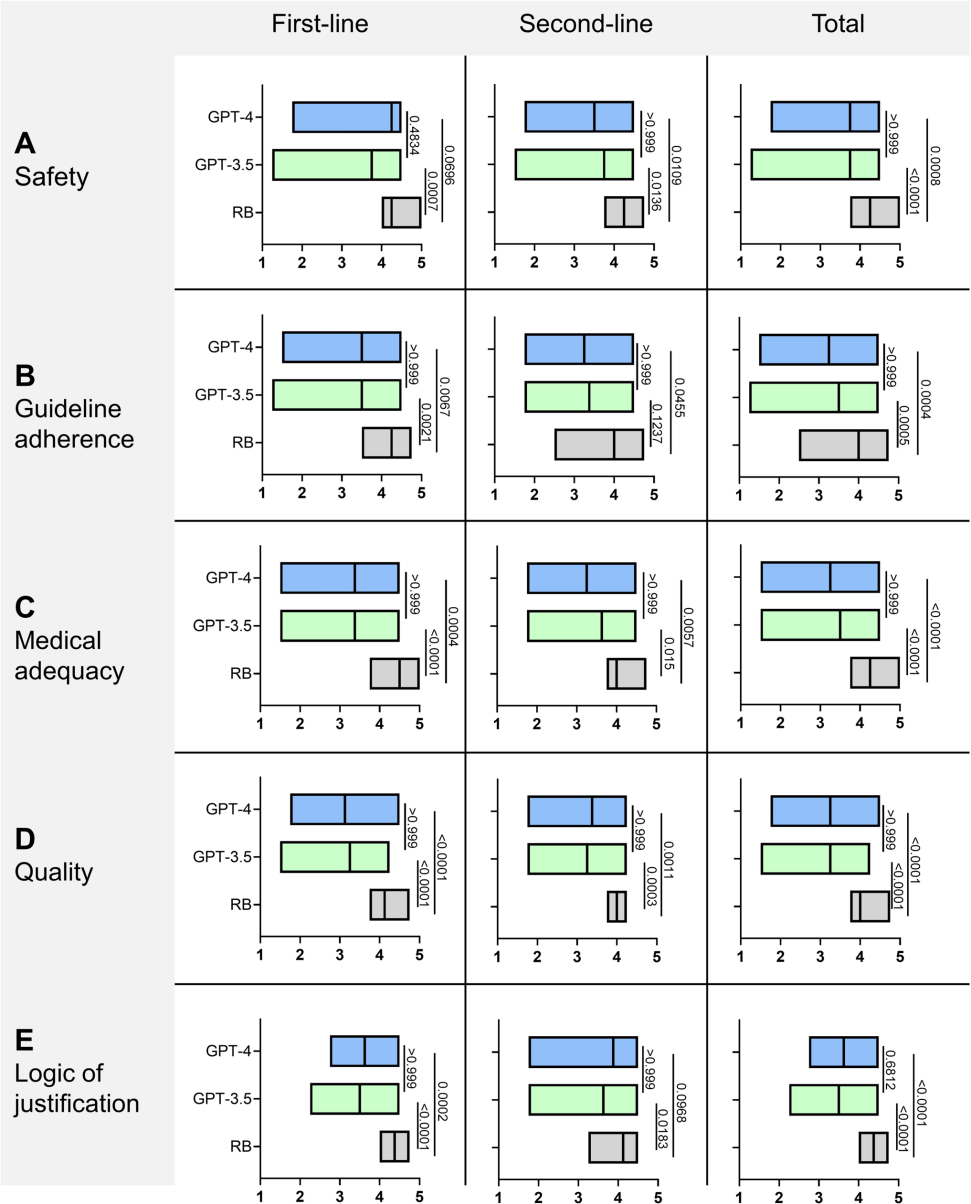
Safety, guideline adherence, medical adequacy, quality and logic of justification. The responses of the RB, GPT-3.5 and GPT-4 were evaluated using criteria (**A**–**E**) on a 5-point Likert scale (x-axis). The median is marked as line in the graphs and P values are reported. *RB* rheumatology board

On a case-based majority assessment for the first-line treatment plans, the majority preferred RB plans (15/20 (75%)), see Fig. 2C, and there was a tie in 5/20 (25%) of the cases. For the second-line treatment plans in 4/20 (20%) of the cases, LLM concepts were favored, and there was a tie in 7/20 (35%) of the cases, see Fig. 3C). Overall, 4/40 (10%) of patient case vignettes were decided in favor of the LLM plans, 12/40 (30%) resulted in a tie, and 24/40 (60%) were decided in favor of the RB plan, see Fig. 2C.

### Treatment plan safety

The median safety of the first- and second-line (total) treatment plans of the RB, GPT-3.5, and GPT-4 was rated 4.25 (range 3.75–5.0), 3.75 (1.25–4.5) and 3.75 (1.75–4.5), respectively. No significant differences were observed between the RB and GPT-4 regarding the safety of the first-line treatment plans, see Fig. 3A, indicating a numerical superiority of GPT-4 compared to GPT-3.5.

### Guideline adherence

The median guideline adherence of the total treatment plans of the RB, GPT-3.5 and GPT-4 was rated 4.0 (range 2.5–4.75), 3.5 (1.25–4.5) and 3.25 (1.5–4.5), respectively. The RB’s ratings scored significantly higher than those of GPT-3.5 (P = 0.0005) and GPT-4 (P = 0.0004), see Fig. 3B.

### Medical adequacy

The median medical adequacy of the total treatment plans of the RB, GPT-3.5, GPT-4 was rated 4.25 (3.75–5.0), 3.5 (1.5–4.5) and 3.25 (1.5–4.5), respectively. The RB was rated significantly better than GPT-3.5 and GPT-4 (P < 0.0001 for both, see Fig. 3C.

### Overall quality

The median quality of the total treatment concepts of the RB, GPT-3.5 and GPT-4 was rated 4.0 (3.75–4.75), 3.25 (1.5–4.25) and 3.25 (1.75–4.5), respectively. The rating for the RB was significantly higher than for GPT-3.5 and GPT-4 (P < 0.0001 for both), see Fig. 3D.

### Logic of the treatment plan justification

The median logic of justification of the treatment concepts of the RB, GPT-3.5 and GPT-4 was rated 4.375 (range 4.0–4.75), 3.5 (2.25–4.5) and 3.625 (2.75–4.5), respectively. The ratings of both LLM were significantly lower compared to RB, with the exception of GPT-4 in second-line therapy (difference compared to RB not significant), indicating a numerical superiority of GPT-4 compared to GPT-3.5, see Fig. 3E.

### Completeness of the treatment plans

**Fig. 4:**
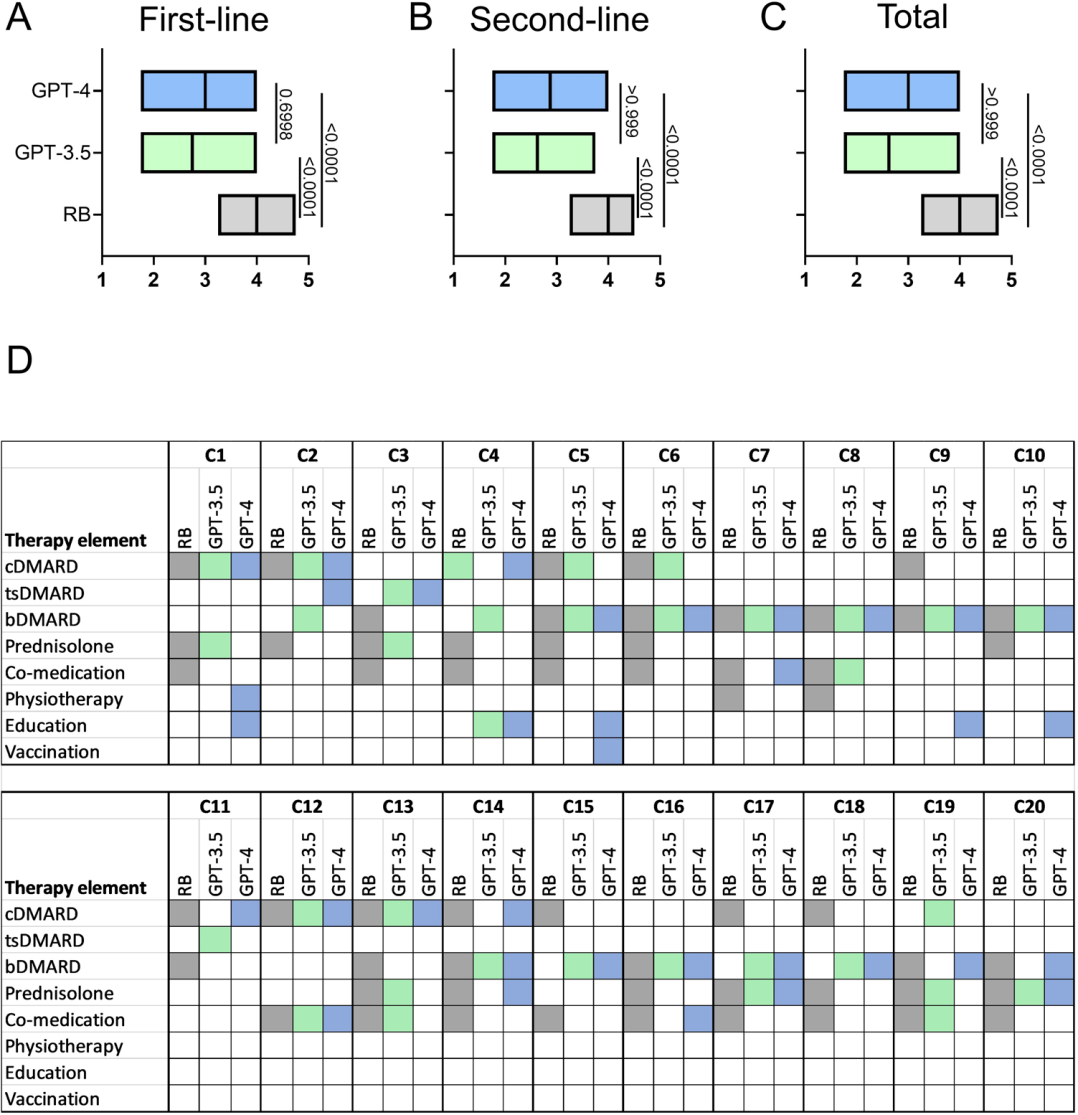
Completeness of the therapy concepts. The various therapy elements were color-coded: grey/colored: mentioned in the therapy concept, white: not mentioned. *C* case, *RB* rheumatology board, *cDMARD* conventional disease-modifying antirheumatic drug, *tsDMARD* targeted synthetic disease-modifying antirheumatic drug, *bDMARD* biological disease-modifying antirheumatic drug

The median completeness of the treatment concepts of the RB, GPT-3.5 and GPT-4 was rated 4.0 (range 3.25–4.75), 2.625 (1.75–4.0) and 3.0 (1.75–4.0), respectively (P < 0.0001), see Fig. 4. The RB’s ratings scored significantly higher than those of GPT-3.5 and GPT-4. A more detailed analysis of the therapy elements that were included in the LLM and RB treatment plans, see Fig. 3D, showed that LLM occasionally suggested tsDMARDs (particularly Janus kinase inhibitors), which the RB completely omitted. Prednisolone and co-medications like vitamin D, folic acid, and antibiotic prophylaxis were more frequently mentioned by the RB. On the other hand, the LLM occasionally included educational measures and vaccinations, which the RB never did.

### Patient vignette difficulty

The vignette collection included cases with a wide variation of difficulty levels. According to the ratings of the RB (range 2–5, easy to difficult) and the four raters (median range 1–4, very easy to difficult), median difficulty level was 3 (moderate). The ratings did not correlate with the case difficulty levels except for a marginally significant negative correlation with safety assessments for the RB treatment plans, see Supplementary Table 2.

### Interrater agreement

Interrater agreement on patient case difficulty was fair, with an ICC of 0.27 (95% CI 0.07–0.53). For safety, adherence, overall quality, logic of treatment plan justification, and completeness, the RB's interrater agreement was poor for both first- and second-line treatment plans. In contrast, the LLM's ratings showed slight, fair or moderate agreement, see Supplementary Table 3. Regarding the overall therapy concept preference, agreement was poor for first-line treatment plans (Fleiss’ kappa coefficient: − 0.03, 95% CI − 0.17 to 0.11) and fair for second-line treatment concepts (Fleiss’ kappa coefficient: 0.24, 95% CI 0.11–0.37).

### Treatment plan length

**Fig. 5:**
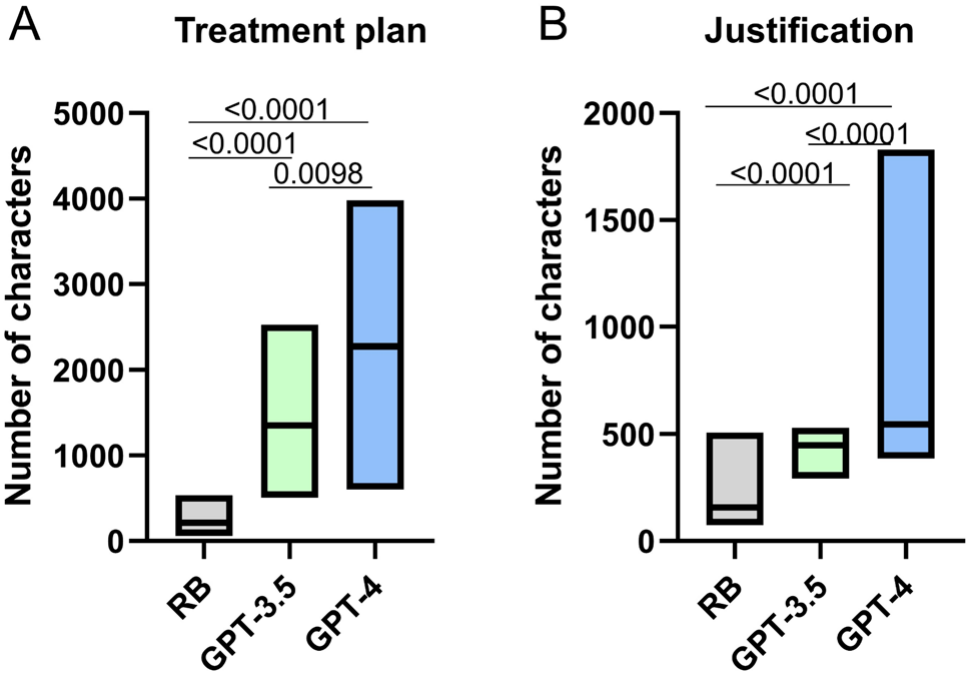
Number of characters of GPT responses. Number of characters of **A** the first- and second-line therapy concepts and **B** the rationales for the therapy concepts are compared (n = 40). *RB* rheumatology board, *GPT* generative pre-training transformer

Compared to the RB (216.5 characters, 60–536), the median character count for the written therapy concepts was over six times higher for GPT-3.5 (1352 characters, 506–2521) and more than ten times higher for GPT-4 (2274.5 characters, 604–2603) (P < 0.0001), as shown in Fig. 5A. There was no restriction on the length of the therapy concept descriptions. However, the justification was required to be formulated in a maximum of two sentences. Despite this, GPT-4 responses were significantly longer than those of GPT-3.5 for both the treatment concepts (P = 0.0098) and the justifications (P < 0.0001), see Fig. 5B.

## Discussion

This study aimed to compare treatment plans generated by GPT-3.5, GPT-4 and rheumatologists for rheumatic patients. Overall, rheumatologist-derived treatment plans were preferred in the majority of cases by blinded rheumatologists. In addition, RB treatment plans were rated significantly better regarding all investigated parameters except for the safety of the first-line GPT-4 treatment plans and the logic of justification of the second-line GPT-4 treatment plans. Overall, inter-rater agreement was low, indicating notable disagreement among raters regarding the quality and preferences of treatment concepts. This underlines the potential of treatment decision support systems.

A closer examination of the individual treatment decisions revealed that rheumatologists’ plans were shorter and more nuanced, whereas the LLM treatment plans were lengthier and more formulaic. For instance, the rheumatology board (RB) made situational decisions favoring dose increases (case 10) of established therapies and local steroid injections (case 2). In contrast, the LLM consistently recommended changes in DMARD therapy.

Non-pharmacological therapies were not regularly mentioned by the LLM and the RB, despite physiotherapy being a fundamental component of treatment for conditions like axial spondylarthritis, as reflected in the 2022 guidelines[12].

Relevant comedication, such as cotrimoxazole for pneumocystis prophylaxis during treatment with cyclophosphamide or rituximab alongside prednisolone therapy[13], entecavir prophylaxis for hepatitis B status under rituximab therapy[14], antibiotic prophylaxis for latent tuberculosis and planned TNF inhibition[14] or vitamin D prophylaxis for prednisolone treatments[15], was sometimes, but not regularly recommended by the LLM (cases 5, 7, 13, 17–20).

In defense of the LLM, it can be argued that the absence of certain considerations was due to the lack of explicit prompts. However, comedication and non-pharmacological therapies were not excluded from the prompts, underscoring the importance of prompting using LLMs like OpenAI's ChatGPT in clinical practice: what is not explicitly mentioned may likely be overlooked. This necessitates situational optimization of prompting, implying that the required response is somewhat anticipated and that prior knowledge is needed to identify gaps in the responses. Venerito et al. recently suggested that prompt engineering, which involves the systematic design and refinement of prompts to enhance a model's performance on specific tasks, could become a crucial skill in rheumatology [16]. Future research could focus on developing comprehensive yet standardized and consented treatment plan prompts for inflammatory rheumatic diseases.

The LLM treatment plans contained several incorrect or at least debatable recommendations. ChatGPT-4 repeatedly listed hydroxychloroquine (quensyl) as a treatment option for RA (cases 1 and 4), despite prompts for guideline-compliant treatments. According to the EULAR 2022 guideline [1], hydroxychloroquine should only be chosen if the other csDMARDs are contraindicated or not tolerated. Rituximab was sometimes given in incorrect dosages or application intervals (cases 14 and 19). GPT-4 recommended the JAK inhibitor baricitinib to a patient with breast cancer and deep vein thrombosis (case 3), despite the EMA recommendations in this regard and the results of the ORAL Surveillance study [17]. In the patient with high-risk antiphospholipid syndrome (APS) and a history of venous thromboembolism while taking the direct oral anticoagulant (DOAC) apixaban, the RB advocated a switch to a vitamin K antagonist, while the LLM would continue the DOAC. While the EULAR guideline allows for continuation of therapy in the absence of arterial thromboses [18], it does not specifically recommend it. However all four raters unanimously preferred changing the therapy to a vitamin K antagonist. This preference appears to have no alternative given the uncertain study situation regarding DOACs and APS [19], as well as current German guidelines [20] and warnings [21].

In the case of SSc (case 15), it is important to note that at the time of the study the current guideline was from 2017 [22] and did not yet include therapies such as tocilizumab [23], rituximab [24], and antifibrotic therapy with nintedanib [25], whose effectiveness are proven. Both the LLM and the RB selected these newer guideline-incompliant therapy options, demonstrating the LLM's capacity for intelligent adaptation and application of up-to-date medical knowledge.

Not only the medical treatment decisions, but also their justifications were rated significantly better. Compared to the LLM, the RB's justifications were more concise, more concrete, and directly related to the patient case. In contrast, the LLM often provided general explanations about why the chosen medication is effective for the disease. Elsewhere, the RB's answers were more critical, for example by pointing to a differential diagnosis, namely drug abuse with an unusual dual-positive MPO- and PR3-ANCA positive status in the context of small vessel vasculitis (case 18).

The lack of significant differences between GPT-3.5 and GPT-4 was surprising, as other studies, such as those evaluating performance on the Spanish rheumatology exam [5], showed that the more advanced GPT-4 performed significantly better. Compared to OpenAI's free version ChatGPT-3.5, the paid version GPT-4 includes more recent content. The significantly higher word count in ChatGPT-4 responses did not result in significantly better evaluations. None of the treatment decisions made by the LLMs included therapies approved after 2022. In contrast, the RB sometimes opted for more modern treatments, such as anifrolumab, which was approved by the EMA for SLE in 2022 (case 14), and bimekizumab (approved by the EMA for SpA in 2023).

Our results are consistent with study results from other disciplines, in which ChatGPT produced partly presentable results with regard to therapy decisions in e.g. (breast) cancer [10, 26] and urological diseases [27], and partly made inadequate, inaccurate and dangerous therapy decisions. Large performance variations were previously observed between different LLMs and according to the individual cases [28], in line with our results. Our study demonstrates that ChatGPT is still inadequate for routine use in therapy decision-making in rheumatology and cannot replace physicians in the foreseeable future. Despite unresolved ethical concerns about accountability, transparency, and health data security, it is impressive how quickly large language models (LLMs) can generate high-quality and safe treatment plans even for complex and rare inflammatory rheumatology cases.

The further development of LLMs and, for example, the addition of retrieval-augmented generation (RAG), which provides the model with additional clinical information, and grounding, which links the model’s responses to real-world data and established knowledge bases [29], could also improve LLMs with regard to treatment decisions in rheumatology [30]. Additional input of multimodal data, such as imaging data could further enhance LLM performance. Truhn et al. previously reported that GPT-4 generated clinically useful orthopedic recommendations solely based on MRI reports [31].

The study has several strengths and limitations. To our knowledge, this is the first study comparing rheumatology treatment concepts generated by LLMs and rheumatologists. The inclusion of various different diseases with varying difficulty levels and clinical pitfalls represent methodological strengths of this study. Furthermore, the multicenter nature of the study represent strengths. A major confounder is that the consistently longer responses from the LLM might have inadvertently unblinded the raters concerning treatment plan source. While more specific prompting including a certain answer length could have been employed, simple and uniform prompts were intentionally chosen to simulate real-world usage, where the LLMs would be used without extensive time and expert knowledge in prompt engineering. Future studies are warranted with a similar answer length to enable better blinding of raters. The board represented a group-based decision, enabling a solid gold-standard for comparison, yet most clinical decisions are seldomly derived in groups. Low inter-rater agreement is a limitation of this study, however this supports the need for decision support. Future studies could investigate whether LLMs might increase overall agreement, treatment confidence, and guideline adherence.

## Conclusion

LLMs demonstrated the ability to generate mostly safe and high-quality treatment concepts for various rheumatic diseases. While models like GPT-4 showed promise in creating treatment plans, they frequently fell short of the nuanced, situation-specific decisions made by experienced rheumatologists. Consequently, rheumatologists generally preferred the treatment concepts generated by the RB over those from LLMs, highlighting that LLMs cannot replace rheumatologists in making therapeutic decisions for rheumatic diseases. Future research should aim to enhance LLMs' performance through standardized prompting strategies and investigate the impact of LLM usage on clinical decisions.

## Supplementary Information

Below is the link to the electronic supplementary material.

Supplementary file1 (PDF 1089 KB)

Supplementary file2 (DOCX 32 KB)

## Data Availability

The patient cases and answers from the rheumatology board and ChatGPT are attached to the manuscript as a supplement. The raw data supporting the conclusions of this article will be made available by the authors upon reasonable request.
