# Correction: Vignette-based comparative analysis of ChatGPT and specialist treatment decisions for rheumatic patients: results of the Rheum2Guide study

**DOI:** 10.1007/s00296-024-05705-2

**Published:** 2024-08-24

**Authors:** Hannah Labinsky, Lea-Kristin Nagler, Martin Krusche, Sebastian Griewing, Peer Aries, Anja Kroiß, Patrick-Pascal Strunz, Sebastian Kuhn, Marc Schmalzing, Michael Gernert, Johannes Knitza

**Affiliations:** 1 Department of Internal Medicine 2, Rheumatology/Clinical Immunology, University Hospital Würzburg, Oberdürrbacher Straße 6, 97080 Würzburg, Germany; 2 Division of Rheumatology and Systemic Inflammatory Diseases, III. Department of Medicine, University Medical Center Hamburg-Eppendorf, Hamburg, Germany; 3 Institute for Digital Medicine, University Hospital Giessen-Marburg, Philipps University, Baldingerstrasse, Marburg, Germany; 4 Stanford Center for Biomedical Informatics Research, Stanford University School of Medicine, Palo Alto, CA USA; 5 Department of Rheumatology, Immunologikum, Hamburg, Germany; 6 AGEIS, Université Grenoble Alpes, Grenoble, France

**Rheumatology International**

10.1007/s00296-024-05675-5

In this article, the first and last author names were swapped and incorrectly written as Labinsky Hannah · Nagler Lea‑Kristin · Krusche Martin · Griewing Sebastian · Aries Peer · Kroiß Anja · Strunz Patrick‑Pascal · Kuhn Sebastian · Schmalzing Marc ·Gernert Michael · Knitza Johannes

The original article has been corrected.

