# Supplementary material for: Vignette-based comparative analysis of ChatGPT and specialist treatment decisions for rheumatic patients: results of the Rheum2Guide study
Source: Rheumatol Int. 2024 Aug 10;44(10):2043–53. doi: 10.1007/s00296-024-05675-5 (PMC11392980; doi:10.1007/s00296-024-05675-5)
Supplement: Supplementary file 1 — Supplementary file1 (PDF 1089 KB) [file 296_2024_5675_MOESM1_ESM.pdf]

Fictional patient vignettes and treatment concepts

CASE VIGNETTE 1:

- Age: 81 years (late onset disease);
- Sex: female;
- Body height in cm: 152; Body weight in kg: 47; BMI: 20.1 kg/m^2;
- Diagnosis: Rheumatoid arthritis (RA);
- Disease duration: 3 months;
- Previous illnesses: diabetes mellitus type 2, chronic kidney dysfunction; dyslipidemia; arterial hypertension;
- Current medical history: The patient reports initial swelling and pain in both wrists and multiple metacarpophalangeal (MCP) joints and proximal interphalangeal (PIP) joints for 3 months. She suffers from morning stiffness lasting 2 hours. She had lost 2 kg in weight during this time and was suffering from heavy night sweats.
- Current clinical examination: tenderness and swelling of both wrists, MCP joint 2 and 3 on both sides and PIP 2 and 3 on both sides. The further physical examination is unremarkable;
- Swollen joint count (SJC): 5;
- Tender joint count (TJC): 10;
- Visual Analogue Scale (VAS) pain scoring: 9 (reference range 0-10);
- Anti-cyclic citrullinated peptide (CCP) titer: positive;
- Rheumatoid factor (RF): positive;
- C-reactive protein (CRP): 1.37 mg/dl (reference range < 0.5 mg/dl);
- Erythrocyte sedimentation rate (ESR): 67.0 mm/h (reference range 3-8 mm/h);
- Other lab results: estimated glomerular filtration rate (eGFR) 29 ml/min (reference range ≥ 60 ml/min), aspartate aminotransferase AST/GOT 25 U/l (reference range 10-35 U/l), alanine aminotransferase ALT/GPT 27 U/l (reference range 10-35 U/l), uric acid 3.8 mg/dl (reference range 2.4-5.7 mg/dl);
- Previous therapies for rheumatoid arthritis: none;
- Medications taken regularly: lercanidipine, rosuvastatine, long- and short-acting insulins;
- Current DAS-28: 5.46 (high disease activity);
- Current X-ray findings: hands on both sides: no erosions, polyarthrotic changes, especially of the distal interphalangeal (DIP) joints and PIP joints;
- Arthrosonography of the right wrist: degeneratively altered osseous structures. No recognizable erosions. Echo-free proliferation of substance in the carpal joints. Grade III hyperperfusion, short anechoic rim around the extensor and flexor carpal tendons. Median nerve sonographically unremarkable;
- Arthrosonography of the MCP joints 2 and 3 on the right: rounded, degeneratively altered osseous structures with grade III capsular dilatation, grade II hyperperfusion.

TREATMENT CONCEPTS FOR CASE VIGNETTE 1:

	Therapy Concept	Reasoning behind the therapy concept	Alternative therapy concept	Reasoning behind the alternative therapy concept
**RB**	Start Leflunomide 20mg orally daily. Prednisolone 15mg daily (tapering in 2.5mg steps and stopping after 4 weeks). Colecalciferol 1000IE daily (as long as prednisolone is taken)	Methotrexate would be the first choice, but renal function is impaired and therefore contraindicated.	Initiate Sulfasalazine 1000mg twice a day. Prescribe prednisolone 15mg daily (tapering in 2.5mg steps and stopping after 4 weeks) and Colecalciferol 1000IE daily (as long as prednisolone is taken).	Sulfasalazine might have a weaker anti-rheumatic effect than leflunomide, but is recommended as alternative treatment, when methotrexate is contraindicated.
**GPT-3.5**	Based on the provided information and considering the EULAR guidelines, a suitable therapy suggestion for this patient would be initiating treatment with a conventional synthetic disease-modifying antirheumatic drug (csDMARD) in combination with glucocorticoids, given the high disease activity.<br><br>Specifically, I would recommend starting the patient on methotrexate (MTX), a first-line csDMARD, at a low dosage due to her age and comorbidities. The initial dosage could be 7.5 mg orally once weekly, with gradual escalation as tolerated, under close monitoring of liver and renal function, given her chronic kidney dysfunction and abnormal liver enzymes.<br><br>Additionally, considering the patient's high disease activity and the need for rapid control of inflammation, a short-term oral glucocorticoid such as prednisone could be initiated at a low to moderate dose to help alleviate symptoms and inflammation while waiting for the DMARD to take effect. A typical starting dose might be 5-10 mg orally once daily, which can be tapered down over time as disease activity improves.<br><br>This combination therapy approach aligns with the current EULAR recommendations for managing rheumatoid arthritis, aiming to achieve early disease control and prevent joint damage and functional impairment, while also considering the patient's age and comorbidities. Regular monitoring of disease activity, laboratory parameters, and adverse effects is essential to optimize treatment efficacy and safety.	The recommendation to initiate methotrexate (MTX) as a first-line conventional synthetic DMARD (csDMARD) is supported by its well-established efficacy in controlling rheumatoid arthritis (RA) disease activity and slowing disease progression. Additionally, the adjunctive use of glucocorticoids helps to swiftly alleviate symptoms and inflammation, aiming for rapid disease control while waiting for the DMARD to achieve its full therapeutic effect.	An alternative therapy concept, considering the patient's comorbidities and high disease activity, could involve initiating therapy with a biologic disease-modifying antirheumatic drug (bDMARD) in combination with a csDMARD.<br><br>Specifically, I would recommend starting the patient on tocilizumab, a biologic agent targeting interleukin-6 (IL-6) receptors, along with methotrexate (MTX) as a conventional synthetic DMARD. The dosage of tocilizumab typically starts at 4 mg/kg intravenously every 4 weeks, adjusted based on the patient's response and tolerability. MTX can be initiated at 7.5 mg orally once weekly, with gradual escalation as tolerated.<br><br>This combination therapy approach offers a potent anti-inflammatory effect and disease control, addressing the patient's high disease activity while considering her comorbidities. Tocilizumab's mechanism of action targeting IL-6 provides an alternative therapeutic option for patients who may not respond adequately to conventional DMARDs alone. Regular monitoring of disease activity, laboratory parameters, and adverse effects remains crucial for optimizing treatment outcomes and patient safety.	The recommendation to initiate tocilizumab, a biologic targeting interleukin-6 (IL-6) receptors, along with methotrexate (MTX) is supported by its efficacy in achieving rapid and sustained control of disease activity, particularly in patients with high disease activity and inadequate response to conventional DMARDs alone. This combination provides a potent anti-inflammatory effect while also considering the patient's comorbidities, offering an alternative therapeutic approach to achieve disease control and improve outcomes.

**GPT-4**	Given the patient's profile and the details provided, including her age, comorbidities (diabetes mellitus type 2, chronic kidney dysfunction, dyslipidemia, arterial hypertension), and the specific characteristics of her rheumatoid arthritis (RA), the therapy suggestion must be carefully tailored to minimize risks while effectively managing her RA. Considering the most current European League Against Rheumatism (EULAR) guidelines for the management of RA, a key aspect is to prioritize safety, especially renal function, and the potential for drug interactions given her comorbid conditions and current medications.<br><br>For this patient, Methotrexate, which is often considered a first-line treatment for RA due to its efficacy in controlling disease activity and slowing disease progression, might not be the best choice primarily due to her chronic kidney dysfunction. Methotrexate is renally excreted and can accumulate in patients with reduced renal function, leading to toxicity.<br><br>An alternative that is often considered in RA patients with renal impairment or when Methotrexate is contraindicated or not tolerated is Hydroxychloroquine. Hydroxychloroquine has a favorable safety profile, especially concerning renal function and is considered effective for mild to moderate RA. It also has the benefit of a low risk of exacerbating comorbid conditions such as diabetes mellitus and cardiovascular diseases.<br><br>For this patient, considering her advanced age, renal impairment, and comorbid conditions, a suitable single therapy suggestion following the EULAR guidelines could be:<br><br>Hydroxychloroquine: The typical dosage for RA management is up to 5 mg/kg real body weight per day. Given her body weight of 47 kg, the dose would be calculated based on the 5 mg/kg recommendation. However, dosing should be initiated cautiously and adjusted based on clinical response and tolerability, taking into account her renal function and potential for drug interactions. It's crucial to monitor the patient closely for any adverse effects, especially considering her age and comorbid conditions. Regular follow-ups to assess efficacy, tolerance, and safety of the therapy, including monitoring of her renal function, are essential. Additionally, since RA is a systemic disease, a comprehensive approach that includes physical therapy, patient education, and possibly the involvement of a multidisciplinary team may enhance her quality of life and overall disease management.<br><br>Given the complexity of RA management, especially in patients with significant comorbidities and late-onset disease, these recommendations should be considered in conjunction with specialist (rheumatologist) advice and tailored to the individual patient's needs, preferences, and medical history.	I suggested Hydroxychloroquine for the following reasons: Firstly, its renal safety profile makes it suitable for patients with chronic kidney dysfunction, as it is not primarily excreted by the kidneys and thus presents a lower risk of toxicity in this population. Secondly, Hydroxychloroquine's efficacy in managing mild to moderate RA symptoms and its relatively low risk for significant adverse effects align well with the patient's clinical profile, which includes advanced age, multiple comorbidities, and the need for a medication that is unlikely to exacerbate existing conditions or interact adversely with her current medications.	Considering the patient's specific circumstances, including advanced age, chronic kidney dysfunction, and other comorbidities, an alternative therapy concept that minimizes renal involvement and is suitable for individuals with contraindications or intolerance to conventional DMARDs like Methotrexate or Hydroxychloroquine would be the use of a biological DMARD (bDMARD). Among bDMARDs, Tocilizumab, an interleukin-6 receptor inhibitor, emerges as a suitable option because of its mode of administration and efficacy profile.<br><br>Tocilizumab: For rheumatoid arthritis, it can be administered as an intravenous infusion or a subcutaneous injection. The subcutaneous route may be more convenient and preferable for this patient, considering her chronic conditions and the goal of minimizing hospital visits. The standard subcutaneous dose is 162 mg administered once every week or once every other week, depending on the patient's clinical response and tolerability.<br>Tocilizumab's efficacy in reducing symptoms of RA, including joint swelling and pain, as well as its ability to slow radiographic progression, makes it a compelling option. Its dosing flexibility and subcutaneous administration route also support adherence and patient comfort. Importantly, while Tocilizumab is generally well-tolerated, monitoring for potential adverse effects, such as infections, elevated liver enzymes, and lipid profile changes, is essential. This therapy choice takes into account the need for effective disease management alongside a favorable safety profile given the patient's renal impairment and overall health status. Regular follow-up and monitoring are crucial to ensure the therapy's efficacy and safety, adjusting as necessary based on the patient's response and any side effects.	Tocilizumab was chosen as an alternative therapy because it directly targets the interleukin-6 pathway, which is significantly involved in the inflammatory process of rheumatoid arthritis, offering potent disease control without reliance on renal excretion, making it suitable for patients with renal impairment. Additionally, its administration via subcutaneous injection provides a convenient option for patients, potentially enhancing adherence and allowing for flexibility in managing RA while minimizing the risk of adverse effects associated with traditional DMARDs in individuals with complex comorbid conditions.

CASE VIGNETTE 2:

- Age: 54 years;
- Sex: male;
- Body height in cm: 174; Body weight in kg: 89; BMI: 29.4 kg/m^2;
- Diagnosis: Rheumatoid arthritis (RA);
- Disease duration: 2 years;
- Previous illnesses: none;
- Current medical history: The patient reports progredient swelling and pain in the right knee joint and right wrist for 2 weeks. The joint complaints had started as part of an upper respiratory tract infection, which has now been completely overcome.
- Current clinical examination: tenderness and swelling of the right knee joint and the right wrist. The further physical examination is unremarkable;
- Swollen joint count (SJC): 2;
- Tender joint count (TJC): 2;
- Visual Analogue Scale (VAS) pain scoring: 7 (reference range 0-10);
- Anti-cyclic citrullinated peptide (CCP) titer: negative;
- Rheumatoid factor (RF): positive;
- C-reactive protein (CRP): 0.4 mg/dl (reference range < 0.5 mg/dl);
- Erythrocyte sedimentation rate (ESR): 9 mm/h (reference range 3-8 mm/h);
- Other lab results: estimated glomerular filtration rate (eGFR) 92 ml/min (reference range ≥ 60 ml/min), aspartate aminotransferase AST/GOT 153 U/l (reference range 10-35 U/l); alanine aminotransferase ALT/GPT 170 U/l (reference range 10-35 U/l);
- Tuberculosis elispot: negative;
- Hepatitis B serology: negative;
- Previous therapies for rheumatoid arthritis: methotrexate (MTX, ongoing);
- Medications taken regularly: methotrexate 20 mg subcutaneously once per week; Folic acid 5 mg 24 h after methotrexate; ibuprofen 1600 mg daily;
- Current DAS-28: 2.82 (low disease activity);
- Current X-ray findings: Chest X-ray: No effusions, no infiltrates, no post-specific changes;
- Current arthrosonography: Puncture-worthy joint effusion of the right knee joint with hyperperfusion grade III. Hypoechoic carpal substance increase with hyperperfusion grade II.

TREATMENT CONCEPTS FOR CASE VIGNETTE 2:

	Therapy Concept	Reasoning behind the therapy concept	Alternative therapy concept	Reasoning behind the alternative therapy concept
**RB**	Administer local therapy of the right knee and right wrist with intraarticular injection of triamcinolonacetonid 20mg and 10mg, respectively. Methotrexate therapy is continued unchanged.	Low disease activity might have been triggered by infection, so methotrexate might still be efficatious, and only a temorary increase in medication is needed.	Stop Methotrexate. Begin leflunomide 20mg daily. Prescribe prednisolone 15mg daily (tapering in 2.5mg steps and stopping after 4 weeks). Add Colecalciferol 1000IE daily (as long as prednisolone is taken).	The csDMARD is changed because there are no negative prognostic parameters, but the treatment goal (remission) has not yet been achieved.
**GPT-3.5**	Based on the provided information and considering the European League Against Rheumatism (EULAR) guidelines, the recommended therapy for this patient could be to add a biologic disease-modifying antirheumatic drug (bDMARD) to their current regimen of methotrexate (MTX) and ibuprofen. Specifically, considering the patient's low disease activity (DAS-28: 2.82), positive rheumatoid factor (RF), and tender/swollen joint counts, along with the ongoing joint symptoms despite MTX therapy, initiating treatment with a tumor necrosis factor-alpha (TNF-α) inhibitor would be appropriate.<br><br>The specific drug recommendation, adhering to the guideline of mentioning only one substance from the same group, would be:<br><br>Adalimumab: 40 mg subcutaneously every other week in addition to the current MTX therapy.<br><br>Adalimumab is a TNF-α inhibitor and is recommended in patients with rheumatoid arthritis who have inadequate response to conventional synthetic DMARDs like methotrexate, which seems to be the case for this patient. The dosage and frequency mentioned are in accordance with standard prescribing practices.<br><br>It's important to note that the decision to initiate bDMARD therapy should be made in consultation with a rheumatologist, taking into consideration the patient's individual clinical characteristics and preferences, as well as potential contraindications or comorbidities. Regular monitoring for efficacy and safety is also essential in managing rheumatoid arthritis patients receiving biologic therapy.	The addition of adalimumab, a tumor necrosis factor-alpha (TNF-α) inhibitor, to the patient's current methotrexate (MTX) regimen is recommended based on the European guidelines due to persistent disease activity despite MTX therapy, positive rheumatoid factor, and tender/swollen joint counts. Adalimumab has shown efficacy in improving disease control and physical function in rheumatoid arthritis patients with inadequate response to conventional DMARDs like MTX.	An alternative therapy concept, considering the patient's low disease activity and ongoing joint symptoms despite methotrexate (MTX) therapy, could involve switching to a different class of biologic disease-modifying antirheumatic drug (bDMARD) with a distinct mechanism of action. In this case, I would recommend:<br>Rituximab: Initiate with a dosage of 1000 mg intravenously on days 1 and 15, then repeat every 6 months in addition to the current MTX therapy.<br>Rituximab targets B cells and has demonstrated efficacy in patients with rheumatoid arthritis who have inadequate response to conventional synthetic DMARDs like methotrexate. This alternative therapy option provides a different mechanism of action compared to TNF-α inhibitors, potentially offering benefit for patients who do not respond adequately to TNF-α inhibitors or have contraindications to them.	Rituximab, a monoclonal antibody targeting CD20 on B cells, presents an alternative therapy option for patients with rheumatoid arthritis who have not achieved sufficient disease control with conventional synthetic DMARDs like methotrexate. Its distinct mechanism of action offers potential efficacy in patients who may not respond optimally to TNF-α inhibitors or have contraindications to them, thereby broadening the therapeutic options available for managing rheumatoid arthritis.

**GPT-4**	Given the detailed clinical profile of the patient with Rheumatoid Arthritis (RA) who is currently on Methotrexate (MTX) with persistent disease activity, the European League Against Rheumatism (EULAR) guidelines for the management of RA would suggest the optimization of RA treatment to achieve target disease control, considering the patient's liver enzyme elevations, which might be an issue with continuing or escalating conventional synthetic Disease-Modifying Antirheumatic Drugs (csDMARDs) like Methotrexate.<br><br>Given the patient's current disease state, with low disease activity (DAS-28: 2.82) but with signs of active synovitis (puncture-worthy joint effusion and hypoechoic carpal substance increase with hyperperfusion), and considering the elevated liver enzymes (AST and ALT), it's crucial to select a treatment option that is both effective for RA and has a favorable hepatic safety profile. The patient has been on Methotrexate, a cornerstone therapy for RA, but the liver enzyme alterations necessitate a careful approach in further management.<br><br>In this context, the most suitable option, according to the latest EULAR guidelines, would be to introduce a biological DMARD (bDMARD) or a targeted synthetic DMARD (tsDMARD) that can be used in combination with Methotrexate or as monotherapy, particularly if Methotrexate has to be discontinued due to liver enzyme elevations. Among bDMARDs and tsDMARDs, one has to choose a drug with a relatively safer profile regarding hepatic effects.<br><br>Given the need to mention only one substance and considering the patient's profile, Tofacitinib could be a suitable option. Tofacitinib is a Janus kinase (JAK) inhibitor, classified under tsDMARDs, effective in RA treatment as both monotherapy and in combination with Methotrexate. It provides an alternative mechanism of action compared to bDMARDs and could be advantageous in a patient with elevated liver enzymes, although monitoring liver function is still necessary due to potential hepatotoxicity.<br><br>Therapy Suggestion:<br>Drug: Tofacitinib<br>Dosage: 5 mg orally twice daily<br>This recommendation is made with the assumption that the patient's liver function is closely monitored, and any signs of further liver enzyme elevations are addressed promptly. Tofacitinib's efficacy in RA, including in those who have had an inadequate response to Methotrexate, makes it a valuable option in this scenario. However, it's crucial to reassess the patient regularly, considering both the clinical response and the safety profile, including liver enzymes and the risk of infections.<br><br>It's essential to note that while Tofacitinib offers a viable option, the final decision should be based on a comprehensive evaluation of the patient's condition, potential side effects, and contraindications, in close consultation with the patient, considering his preferences and any other comorbidities.	I suggested Tofacitinib for several reasons: Firstly, it's a targeted synthetic DMARD (tsDMARD) effective in patients with RA who have had an inadequate response to Methotrexate, offering an alternative mechanism of action. Secondly, given the patient's elevated liver enzymes, Tofacitinib can be a safer option than increasing the dose of Methotrexate or adding certain other medications that might further compromise liver function, although it still necessitates careful monitoring of liver enzymes due to potential hepatotoxicity.	Considering the patient's condition and the need for an alternative therapy concept to Tofacitinib, particularly due to the raised liver enzymes and the requirement for a drug that might be less hepatotoxic, an appropriate alternative could be the initiation of a biological DMARD (bDMARD) that is typically well-tolerated and effective in controlling RA disease activity. Among bDMARDs, Etanercept stands out as a viable option. Etanercept is a tumor necrosis factor (TNF) inhibitor that can be used in cases where Methotrexate is insufficient alone and has a relatively favorable safety profile, including in the context of liver enzyme elevations.<br><br>Alternative Therapy Suggestion:<br>Drug: Etanercept<br>Dosage: 50 mg administered subcutaneously once weekly<br>Etanercept is chosen for its efficacy in reducing the signs and symptoms of RA, inhibiting the progression of structural damage, and improving physical function in adult patients with moderately to severely active RA. This therapy can be considered as monotherapy or in combination with Methotrexate when the latter cannot be increased due to liver concerns. Importantly, Etanercept does not require regular liver monitoring as strictly as Methotrexate or Tofacitinib but monitoring should still be in place for overall safety.	Etanercept was suggested as an alternative due to its mechanism as a TNF inhibitor, which is effective in controlling rheumatoid arthritis symptoms and preventing joint damage, making it suitable for patients with active disease despite Methotrexate therapy. Its favorable safety profile, including a relatively lower risk of hepatotoxicity compared to conventional synthetic DMARDs and certain other biologics or tsDMARDs, makes it a safer choice for a patient with elevated liver enzymes, while still effectively managing RA symptoms.

CASE VIGNETTE 3:

- Age: 69 years;
- Sex: female;
- Body height in cm: 163; Body weight in kg: 77; BMI: 29.0 kg/m^2;
- Diagnosis: rheumatoid arthritis;
- Disease duration: 10 years;
- Previous illnesses: Deep vein thrombosis and pulmonary artery embolism after a long-haul flight 2 years ago, carcinoma of the left breast and breast-conserving surgery 5 years ago, no recurrence since then;
- Current medical history: Several finger joints have been increasingly swollen and painful for about 3 months. Prednisolone therapy with initial 20 mg for 6 weeks had a transient effect, but now, after reducing the prednisolone, the symptoms are increasing again.
- Current clinical examination: tenderness and swelling of metacarpophalangeal (MCP) joint 2 and PIP joint 2 and 3 of the left hand and MCP joints 2 and 3 of the right hand. The further physical examination is unremarkable;
- Swollen joint count (SJC): 5;
- Tender joint count (TJC): 8;
- Visual Analogue Scale (VAS) pain scoring: 8 (reference range 0-10);
- Anti-cyclic citrullinated peptide (CCP) titer: positive;
- Rheumatoid factor (RF): positive;
- C-reactive protein (CRP): 2.2 mg/dl (reference range < 0.5 mg/dl);
- Erythrocyte sedimentation rate (ESR): 72.0 mm/h (reference range 3-8 mm/h);
- Other lab results: estimated glomerular filtration rate (eGFR) 62 ml/min (reference range ≥ 60 ml/min), aspartate aminotransferase AST/GOT 53 U/l (reference range 10-35 U/l), alanine aminotransferase ALT/GPT 51 U/l (reference range 10-35 U/l);
- Tuberculosis elispot: negative;
- Hepatitis B serology: negative;
- Previous therapies for rheumatoid arthritis: condition after adalimumab and methotrexate over 3 years, secondary effect failure; condition after etanercept over 6 months, primary effect failure;
- Medications taken regularly: tocilizumab for 5 years, prednisolone 5 mg 1-0-0, apixaban 5 mg 1-0-1;
- Current DAS-28: 5.3 (high disease activity);
- Current X-ray findings: Chest X-ray: No effusions, no infiltrates, no post-specific changes;
- Current arthrosonography: Erosive changes in several finger joints. Marked capsular dilation and hyperperfusion grade II-III over MCP joint 2 and 3 of the right hand and MCP joint 2 and proximal interphalangeal (PIP) joints 2 and 3 of the left hand.

TREATMENT CONCEPTS FOR CASE VIGNETTE 3:

	Therapy Concept	Reasoning behind the therapy concept	Alternative therapy concept	Reasoning behind the alternative therapy concept
**RB**	Initiate Rituximab 1g (d1 + d15) i.v.. Prednisolone 15mg daily (tapering in 2.5mg steps until 5mg daily). Add Colecalciferol 1000IE daily (as long as prednisolone is taken).	The reasons were the patient's high disease activity, the elevated transaminases, the history of deep vein thrombosis, the history of underlying malignancy and the treatment failure of adalimumab, etanercept and tocilizumab.	Initiate sarilumab 200mg every other week. Prescribe prednisolone 15mg daily (tapering in 2.5mg steps until 5mg daily). Add Colecalciferol 1000IE daily (as long as prednisolone is taken).	The reasons were the patient's high disease activity, the elevated transaminases, the history of deep vein thrombosis, the history of underlying malignancy and the treatment failure of adalimumab, etanercept and tocilizumab.
**GPT-3.5**	Based on the provided patient information and considering the EULAR guidelines for rheumatoid arthritis, a suitable therapy suggestion would be:<br><br>Therapy Concept:<br>Introduce a targeted synthetic disease-modifying antirheumatic drug (tsDMARD) in combination with the ongoing prednisolone therapy.<br><br>Specific Drug and Dosage:<br>Baricitinib 4 mg once daily, in addition to continuing prednisolone 5 mg once daily.<br><br>Rationale:<br>Baricitinib is a Janus kinase (JAK) inhibitor, which has been shown to be effective in reducing the signs and symptoms of rheumatoid arthritis and inhibiting radiographic progression in patients who have had an inadequate response to conventional synthetic DMARDs (csDMARDs) or biologic DMARDs (bDMARDs). The addition of baricitinib to the existing therapy with tocilizumab and prednisolone aims to achieve better disease control and improvement in symptoms, particularly in patients with high disease activity like the case described. Additionally, the choice of baricitinib takes into account the patient's previous failures with biologic DMARDs (adalimumab and etanercept), making a switch to a targeted synthetic DMARD a reasonable option. However, close monitoring for adverse effects, particularly in relation to liver function (given the elevated AST and ALT levels), renal function (due to the decreased eGFR), and thrombotic events (due to the history of deep vein thrombosis and pulmonary embolism), is essential. Regular follow-up visits should be scheduled to assess the response to therapy and adjust treatment if necessary, adhering to the treat-to-target principle advocated by EULAR guidelines for rheumatoid arthritis management.	The therapy suggestion of adding baricitinib to the existing regimen of tocilizumab and prednisolone is based on the patient's high disease activity, demonstrated inefficacy with previous biologic DMARDs, and the need for a targeted synthetic DMARD to achieve better disease control and symptom improvement, as per EULAR guidelines for rheumatoid arthritis management.	An alternative therapy concept would be:<br><br>Therapy Concept:<br>Switching to a different biologic disease-modifying antirheumatic drug (bDMARD) in combination with the ongoing prednisolone therapy.<br><br>Specific Drug and Dosage:<br>Rituximab 1,000 mg administered as two 500 mg intravenous infusions separated by two weeks, repeated every six months, in addition to continuing prednisolone 5 mg once daily.<br><br>Rationale:<br>Rituximab, a monoclonal antibody targeting CD20-positive B cells, has demonstrated efficacy in rheumatoid arthritis, particularly in patients who have failed to respond adequately to other bDMARDs. Given the patient's previous failures with adalimumab and etanercept, rituximab presents a viable alternative option with a different mechanism of action. The addition of rituximab aims to achieve better disease control and symptom improvement, while the concurrent use of prednisolone helps to manage acute flares. Regular monitoring for infusion reactions and infections, as well as follow-up evaluations to assess treatment response, are essential components of this therapy approach.	The alternative therapy suggestion involves switching to rituximab due to the patient's inadequate response to previous biologic DMARDs, aligning with the EULAR guidelines which recommend considering alternative bDMARDs in such cases. Rituximab's unique mechanism of action targeting CD20-positive B cells offers a different therapeutic approach that may lead to improved disease control in rheumatoid arthritis.

**GPT-4**	Given the detailed patient history, current medical condition, and previous therapy outcomes, along with considering the European League Against Rheumatism (EULAR) guidelines for the management of rheumatoid arthritis (RA), the recommended therapy should focus on efficacy, safety, and the patient's specific comorbidities and previous treatment responses. Given the patient's history of deep vein thrombosis and pulmonary artery embolism, as well as breast cancer, it is essential to choose a therapy that minimizes the risk of adverse effects related to these conditions. Additionally, the patient has a history of secondary and primary failure with TNF inhibitors (adalimumab and etanercept) and is currently on tocilizumab, which suggests an inadequate response or loss of efficacy over time.<br><br>Considering the latest EULAR guidelines and the patient's specific situation, the recommendation would be to switch to a Janus kinase (JAK) inhibitor, given the inadequate response to TNF inhibitors and tocilizumab, an IL-6 receptor antagonist. Among JAK inhibitors, Baricitinib could be considered appropriate due to its once-daily oral administration, which might improve patient compliance and quality of life. Baricitinib has been shown to be effective in RA patients who have had an inadequate response to one or more TNF inhibitors.<br><br>Suggested Therapy:<br><br>Baricitinib, 4 mg once daily orally.<br>This recommendation is based on the drug's efficacy in controlling RA symptoms, its safety profile, and the convenience of oral administration. However, the choice of specific therapy must consider the patient's overall health status, potential drug interactions (especially considering the patient is on apixaban and prednisolone), and monitoring for adverse effects. It's crucial to monitor liver enzymes given the elevated AST and ALT levels and adjust the dose of baricitinib if necessary, especially in the context of the patient's reduced eGFR. Regular monitoring for infections, blood counts, and lipid profiles is also recommended due to the known side effects associated with JAK inhibitors.<br><br>Before initiating baricitinib, it's important to ensure that there are no contraindications, such as active infections. Given the patient's medical history, careful consideration and regular monitoring are advised to manage the risk of thrombosis associated with JAK inhibitors, particularly in patients with a history of thromboembolic events.<br><br>This therapy suggestion is based on the information provided and the most current EULAR guidelines available up to my last knowledge update in April 2023. Clinical decisions should always be made based on the most recent guidelines and the patient's ongoing health status.	The choice of Baricitinib is guided by its mechanism of action as a JAK inhibitor, which offers a different therapeutic pathway compared to the previously used TNF inhibitors and IL-6 receptor antagonist, making it suitable for patients with an inadequate response to those therapies. Additionally, its oral administration route can improve patient compliance, and its efficacy in RA management has been supported by clinical trials, providing a beneficial option for controlling disease activity while considering the patient's complex medical history and the need for careful monitoring of potential side effects.	Given the patient's history and the need for an alternative to JAK inhibitors, considering the specific concerns related to safety and previous treatment failures, a biologic agent from a different class could be a suitable option. Rituximab, a CD20-directed cytolytic antibody, represents an alternative therapeutic strategy, particularly for patients who have had an inadequate response to TNF inhibitors and an IL-6 receptor antagonist.<br><br>Suggested Alternative Therapy:<br><br>Rituximab, administered as two intravenous infusions of 1000 mg each, separated by two weeks, as one treatment course. Repeat courses should be based on clinical evaluation but typically are administered every 6 months.<br>Rituximab targets B cells, which play a crucial role in the pathogenesis of rheumatoid arthritis, leading to a reduction in inflammation and structural joint damage. This therapy is particularly considered for patients with a history of malignancy like breast cancer, as its mode of action is distinct and has been used safely in patients with various types of cancers. The dosing regimen provides the flexibility of monitoring the patient's response and adjusting treatment intervals as needed. It is important to premedicate with methylprednisolone before each rituximab infusion to reduce infusion-related reactions and to screen for hepatitis B virus (HBV) infection to prevent reactivation. This recommendation is based on its efficacy in patients with an inadequate response to anti-TNF therapy and its manageable safety profile, considering the patient's complex medical history.	The recommendation for Rituximab is based on its unique mechanism of action targeting B cells, offering an effective treatment pathway for patients who have not responded adequately to TNF inhibitors and an IL-6 receptor antagonist, thereby addressing the patient's persistent disease activity through a different immunological target. Additionally, Rituximab's established use in patients with a history of malignancy, such as breast cancer, along with its safety profile in the context of autoimmune diseases, makes it a suitable alternative, providing a balance between efficacy and safety for this patient with complex medical history.

CASE VIGNETTE 4:

- Age: 33 years;
- Sex: female;
- Body height in cm: 172; Body weight in kg: 65; BMI: 22.0 kg/m^2;
- Diagnosis: Rheumatoid arthritis (RA);
- Disease duration: 8 months;
- Previous illnesses: none;
- Current medical history: The patient reports pain of several finger joints and the right shoulder for 8 months. She has the desire to have a child. Conception is planned. She and her husband are not currently using contraception;
- Current clinical examination: Tenderness of all proximal interphalangeal (PIP) joints and metacarpophalangeal (MCP) joints 2 to 5 on both sides, swelling of PIP joint 3 of both sides. Pain in movement of the right shoulder. The further physical examination is unremarkable;
- Swollen joint count (SJC): 2;
- Tender joint count (TJC): 9;
- Visual Analogue Scale (VAS) pain scoring: 10 (reference range 0-10);
- Anti-cyclic citrullinated peptide (CCP) titer: negative;
- Rheumatoid factor (RF): positive;
- C-reactive protein (CRP): 0.8 mg/dl (reference range < 0.5 mg/dl);
- Erythrocyte sedimentation rate (ESR): 41 mm/h (reference range 3-8 mm/h);
- Other lab results: Blood count, liver and kidney function values are normal;
- Tuberculosis elispot: negative;
- Hepatitis B serology: negative;
- Previous therapies for rheumatoid arthritis: none;
- Medications taken regularly: none;
- Current DAS-28: 4.8 (moderate disease activity);
- Current X-ray findings: Chest X-ray: No effusions, no infiltrates, no post-specific changes; Hands/ feet X-ray: One erosion at the MCP joint 2 on the right, no significant degenerative changes;
- Current arthrosonography of the right shoulder: Significant capsular dilatation, large draining effusion with synovial proliferation and hyperperfusion II°.

TREATMENT CONCEPTS FOR CASE VIGNETTE 4:

	Therapy Concept	Reasoning behind the therapy concept	Alternative therapy concept	Reasoning behind the alternative therapy concept
**RB**	Start with sulfasalazine 1000mg twice daily . Supplement folic acid 5mg daily. Prednisolone 15mg daily (tapering in 2.5mg steps and stopping after 4 weeks) . Add colecalciferol 1000IE daily (as long as prednisolone is taken).	The patient has an active disease, has currently no DMARD and is planning a pregnancy.	Certolizumab-pegol s.c. 400mg in week 0, 2 and 4, then 200mg every other week and prednisolone 15mg daily (tapering in 2.5mg steps and stopping after 4 weeks). Add colecalciferol 1000IE daily (as long as prednisolone is taken).	The therapy concept would be off-label as first line treatment and is not covered by EULAR recommendations as first line treatment. However, the therapy concept would most likely be highly effective. This regimen should only be applied if sulfasalazine is not possible.
**GPT-3.5**	Based on the provided clinical information and considering the patient's desire to conceive, a recommended therapy option according to the most current European guidelines (EULAR guidelines) for rheumatoid arthritis would be as follows:<br>Methotrexate (MTX) monotherapy is recommended as the initial treatment for rheumatoid arthritis according to the EULAR guidelines. However, due to the patient's desire to conceive, MTX should be avoided.<br>In this case, considering the patient's disease activity and the desire for pregnancy, the recommended therapy would be:<br><br>Therapy Concept:<br>Treat-to-Target Strategy with Biological Disease-Modifying Antirheumatic Drug (bDMARD)<br>Drug Recommendation:<br>Adalimumab<br>Dosage:<br>40 mg subcutaneously every other week<br>Rationale:<br>Adalimumab (a TNF-alpha inhibitor): EULAR guidelines recommend biological DMARDs, such as TNF-alpha inhibitors, as first-line options in patients with moderate to high disease activity who have contraindications to methotrexate or are intolerant to it. Adalimumab has shown efficacy in controlling disease activity and improving symptoms in rheumatoid arthritis. It has also been shown to be relatively safe during pregnancy, with limited evidence of adverse effects on fetal outcomes.<br>Dosing Schedule: Adalimumab is typically administered subcutaneously at a dose of 40 mg every other week, which is the standard dosing regimen.<br>Treat-to-Target Strategy: The goal of treatment is to achieve low disease activity or remission. The patient should be monitored regularly to assess disease activity and treatment response, with adjustments made as necessary to achieve treatment targets.<br>Contraception: Although the patient is currently not using contraception, it is important to discuss the potential risks of pregnancy while on adalimumab therapy and the importance of effective contraception until a decision is made to discontinue or change the treatment regimen.<br>Preconception Counseling: Given the patient's desire to conceive, preconception counseling should be provided to discuss the potential risks and benefits of adalimumab therapy during pregnancy, as well as the possibility of	Adalimumab is recommended due to its efficacy in controlling disease activity and improving symptoms in rheumatoid arthritis, particularly in patients with contraindications or intolerance to methotrexate. Additionally, adalimumab has shown relative safety during pregnancy with limited evidence of adverse fetal outcomes, making it a suitable option for the patient who desires conception.	An alternative therapy concept, considering the patient's desire for conception and the current disease activity, would be:<br><br>Therapy Concept:<br>Conventional Synthetic Disease-Modifying Antirheumatic Drug (csDMARD) Monotherapy with Sulfasalazine<br><br>Drug Recommendation:<br>Sulfasalazine<br><br>Dosage:<br>500 mg orally twice daily, with gradual dose escalation as tolerated, up to a maximum of 2-3 grams daily<br><br>Rationale:<br>Sulfasalazine, a conventional synthetic DMARD, is an alternative option for patients with rheumatoid arthritis who cannot tolerate or have contraindications to methotrexate or biological agents. It has shown efficacy in reducing disease activity and joint inflammation. Moreover, sulfasalazine is considered relatively safe during pregnancy, making it a suitable option for patients planning conception. Regular monitoring for efficacy and adverse effects is recommended during treatment.	Sulfasalazine is recommended as an alternative therapy due to its efficacy in reducing disease activity and joint inflammation in rheumatoid arthritis, particularly in patients unable to tolerate or contraindicated to methotrexate or biological agents. Additionally, sulfasalazine is considered relatively safe during pregnancy, aligning with the patient's desire for conception.

	discontinuing or switching to alternative therapies prior to conception.<br>It's crucial to regularly monitor disease activity, treatment response, and potential adverse effects, as well as to provide appropriate support and counseling regarding family planning and pregnancy throughout the treatment course. Close collaboration between the rheumatologist and obstetrician is recommended to optimize both rheumatoid arthritis management and pregnancy outcomes.			
**GPT-4**	Given the patient's desire for pregnancy and the absence of contraindications based on her medical history, current European League Against Rheumatism (EULAR) guidelines for the management of rheumatoid arthritis (RA) in the context of pregnancy would suggest prioritizing treatment options that are safe for pregnancy. Among the disease-modifying antirheumatic drugs (DMARDs), hydroxychloroquine and sulfasalazine are considered safe during pregnancy, but in the context of your request for a single therapy concept with specific drug names and dosages, and considering the need for effective control of moderate disease activity, one appropriate option could be:<br><br>Hydroxychloroquine: This medication is often used for its mild to moderate efficacy in RA, its safety profile during pregnancy, and its lack of association with adverse pregnancy outcomes. The typical dosage for RA management is 200 to 400 mg daily, administered orally. The exact dosage should be tailored to the patient's weight to not exceed 5 mg/kg/day (based on the ideal body weight), due to the risk of retinopathy with higher dosages.<br>For a 65 kg patient, a common starting dose could be 200 mg twice daily, but considering pregnancy plans and the need to minimize any potential risk, a lower end of the dosing range might be preferable initially or adjusting to maintain efficacy while ensuring safety.<br><br>It's important to note that while hydroxychloroquine is generally well-tolerated, regular ophthalmologic monitoring is recommended due to the risk of retinopathy, especially with long-term use. Additionally, because RA management is highly individualized, ongoing evaluation of disease activity and adjustment of therapy may be necessary to ensure optimal disease control while minimizing risks to both the patient and the potential pregnancy.<br><br>Furthermore, it's crucial for the patient to have a pre-conception counseling session to understand the implications of RA and its treatment on pregnancy and to plan for a pregnancy with the least possible risk of disease flare-ups. This should be complemented by close monitoring of RA activity and adjustments in therapy as needed throughout the pre-conception period, during pregnancy, and postpartum.<br><br>Given the evolving nature of clinical guidelines and the importance of individual patient factors in treatment decisions, consulting the most current EULAR guidelines or a rheumatologist specializing in pregnancy and RA is advisable for	Hydroxychloroquine is recommended for this patient due to its safety profile during pregnancy, making it an ideal choice for someone planning conception, and its effectiveness in managing rheumatoid arthritis symptoms with a low risk of serious side effects. It allows for disease control without posing significant risks to fetal development, aligning with the patient's reproductive plans while addressing moderate disease activity and joint symptoms.	An alternative therapy concept, considering the patient's desire for pregnancy and need for effective RA management, would be:<br><br>Sulfasalazine: This medication is considered safe for use during pregnancy and is effective in treating rheumatoid arthritis. The typical starting dosage for RA is 500 mg orally twice daily, with the possibility to increase up to 2,000-3,000 mg per day in divided doses, based on patient response and tolerance.<br>For a patient planning pregnancy, starting at a lower dose and gradually increasing as needed can help minimize side effects while providing effective disease control. Sulfasalazine's safety profile during pregnancy makes it a suitable option for women with RA who are or wish to become pregnant, offering an effective treatment alternative with a well-established record of use in this patient population.<br>It's important to monitor for side effects, such as gastrointestinal discomfort or rare hematologic abnormalities, and adjust the treatment as necessary in consultation with a healthcare provider.	Sulfasalazine is chosen as an alternative due to its safety during pregnancy and effectiveness in managing rheumatoid arthritis, providing a viable option for women considering conception. It offers a favorable balance between controlling disease activity and ensuring the safety of both the mother and the future child, with a long history of use in pregnancy-associated RA management.

	the most personalized and up-to-date treatment advice.				

CASE VIGNETTE 5:

- Age: 75 years;
- Sex: female;
- Body height in cm: 159; Body weight in kg: 80; BMI: 31.6 kg/m^2;
- Diagnosis: rheumatoid arthritis (RA);
- Disease duration: 25 years;
- Previous illnesses: arterial hypertension, diabetes mellitus type 2
- Current medical history: The patient reports eye pain, sensitivity to light, reduced vision for 2 weeks on the right eye. This is the second episode of scleritis. According to the ophthalmologists, the findings are consistent with sterile non-infectious scleritis. Non-erosive scleritis occurred for the first time 5 years ago and was already considered an ocular manifestation of rheumatoid arthritis at that time. It was treated with systemic prednisolone and methotrexate. Apart from load-dependent joint complaints, there is no clear joint pain.
- Current clinical examination: redness and corneal ulcer of the right eye. Slight chronic swelling of metacarpophalangeal (MCP) joints 1 to 2 on both sides. The further physical examination is unremarkable;
- Swollen joint count (SJC): 2;
- Tender joint count (TJC): 0;
- Visual Analogue Scale (VAS) pain scoring: 1 (reference range 0-10);
- Anti-cyclic citrullinated peptide (CCP) titer: positive;
- Rheumatoid factor (RF): positive;
- C-reactive protein (CRP): 1.1 mg/dl (reference range < 0.5 mg/dl);
- Erythrocyte sedimentation rate (ESR): 52 mm/h (reference range 3-8 mm/h);
- Other lab results: hemoglobin 9.8 g/dl (reference range 12-14.6 g/dl), liver and kidney function values normal;
- Tuberculosis elispot: negative;
- Hepatitis B serology: negative;
- Previous therapies for rheumatoid arthritis: hydroxychloroquine for 10 years, leflunomide for 10 years;
- Medications taken regularly: methotrexate 20 mg subcutaneously once per week for 5 years (ongoing), vitamin d, metformin, ramipril;
- Current DAS-28: 3.17 (moderate disease activity);
- Current X-ray findings: Chest X-ray: no effusions, no infiltrates, no post-specific changes;
- Current arthrosonography: not done;
- Ophthalmologic findings: Slit lamp examination: Findings of scleritis with peripheral ulcerative keratitis.

TREATMENT CONCEPTS FOR CASE VIGNETTE 5:

	Therapy Concept	Reasoning behind the therapy concept	Alternative therapy concept	Reasoning behind the alternative therapy concept
**RB**	Start adalimumab 40mg s.c. every other week and prednisolone 80mg daily (tapering 10mg each week until 40mg/day, then tapering 5mg each week until 20mg/day, then tapering 2.5mg each week until 10mg/day, then tapering 1mg each week until 5mg/day). Add colecalciferol 1000IE daily (as long as prednisolone is taken). Continue methotrexate 20mg s.c. once per week and folic acid 5mg p.o. once per week 24h after methotrexate.	Scleritis in the context of rheumatoid arthritis needs intensification of immunosuppression and glucocorticoid pulse (1mg/kg body weight). No international guidelines exist for this case.	Rituximab 1g (d1, d15) i.v. and prednisolone 80mg daily (tapering 10mg each week until 40mg/day, then tapering 5mg each week until 20mg/day, then tapering 2.5mg each week until 10mg/day, then tapering 1mg each week until 5mg/day). Add colecalciferol 1000IE daily (as long as prednisolone is taken). Continuate methotrexate 20mg s.c. once per week and folic acid 5mg p.o. once per week 24h after methotrexate.	Rituximab may be as efficacious as adalimumab, but takes longer until onset of biological action.
**GPT-3.5**	Based on the provided patient information and considering the most current European guidelines (EULAR guidelines), a suitable therapy concept for this patient with rheumatoid arthritis (RA) and ocular manifestations (scleritis) would involve adding a biologic disease-modifying antirheumatic drug (bDMARD) to the existing treatment regimen. Given the patient's moderate disease activity (DAS-28 of 3.17) despite ongoing methotrexate therapy and previous use of conventional synthetic DMARDs (hydroxychloroquine and leflunomide), the addition of a bDMARD is warranted.<br><br>The specific bDMARD that could be considered, as per EULAR recommendations, is Adalimumab. Adalimumab is a tumor necrosis factor-alpha (TNF-α) inhibitor and is commonly used in the management of RA, particularly in patients with inadequate response to conventional DMARDs.<br><br>The recommended dosage for Adalimumab in RA is 40 mg administered subcutaneously every other week. It can be self-administered by the patient after appropriate training.<br><br>Adalimumab has shown efficacy in improving signs and symptoms of RA, inhibiting structural damage, and reducing ocular manifestations associated with the disease.<br><br>Therefore, the therapy suggestion for this patient would be to continue methotrexate at the current dose of 20 mg subcutaneously once per week and add Adalimumab 40 mg subcutaneously every other week to the treatment regimen. This combination therapy aims to achieve better disease control, including the management of scleritis, and to improve overall outcomes in the management of rheumatoid arthritis. However, the final decision should be made in consultation with the patient's rheumatologist considering individual factors, including comorbidities, preferences, and potential contraindications.	The addition of Adalimumab, a tumor necrosis factor-alpha (TNF-α) inhibitor, is warranted in this patient due to moderate disease activity despite ongoing methotrexate therapy and previous inadequate response to conventional synthetic DMARDs. Adalimumab has demonstrated efficacy in improving both joint symptoms and ocular manifestations associated with rheumatoid arthritis, thereby addressing the patient's current clinical presentation and providing comprehensive disease management.	An alternative therapy concept for this patient, considering the ocular manifestations and moderate disease activity, could involve initiating Tocilizumab, a biologic disease-modifying antirheumatic drug (bDMARD) targeting the interleukin-6 (IL-6) pathway.<br><br>The recommended dosage for Tocilizumab in rheumatoid arthritis is 162 mg administered subcutaneously once weekly. Tocilizumab has shown efficacy in reducing both joint inflammation and ocular manifestations associated with RA, offering a viable alternative to TNF-α inhibitors like Adalimumab.	The recommendation for Tocilizumab stems from its targeting of the interleukin-6 (IL-6) pathway, which plays a crucial role in the pathogenesis of rheumatoid arthritis and associated ocular manifestations. Given the patient's moderate disease activity and previous inadequate response to conventional DMARDs, Tocilizumab offers an alternative mechanism of action to effectively manage both joint symptoms and scleritis.

**GPT-4**	Given the patient's complex clinical picture, including a long-standing history of rheumatoid arthritis (RA), non-infectious scleritis as an extra-articular manifestation of RA, previous therapy with methotrexate and biologics not mentioned, and comorbid conditions such as diabetes mellitus type 2 and arterial hypertension, a targeted therapy approach adhering to the most current European League Against Rheumatism (EULAR) guidelines is essential.<br><br>Considering the patient's moderate disease activity as indicated by a DAS-28 score of 3.17, positive serology for RA (Anti-CCP and RF positive), non-responsiveness to conventional synthetic DMARDs (methotrexate and leflunomide), and the occurrence of extra-articular manifestations (scleritis), the introduction of a biologic DMARD (bDMARD) or targeted synthetic DMARD (tsDMARD) that can address both joint and extra-articular manifestations without worsening the patient's comorbid conditions is recommended.<br><br>Among bDMARDs, Tocilizumab, an IL-6 receptor inhibitor, has shown efficacy in controlling inflammation, reducing disease activity, and improving quality of life in RA patients. It can be effective in patients who have had an inadequate response to one or more DMARDs, including methotrexate. Tocilizumab is available for both intravenous (IV) and subcutaneous (SC) administration. Considering the patient's advanced age, comorbidities, and convenience, the SC route might be preferable.<br><br>Suggested Therapy: Tocilizumab<br><br>Route and Dosage: Subcutaneously, 162 mg once every week.<br>This suggestion takes into account the need for careful monitoring of the patient's response to therapy and potential side effects, given her age, comorbid conditions, and the chronic nature of her RA. Tocilizumab's dosing recommendation aligns with its ability to manage moderate to severe RA in adults who have responded inadequately to one or more DMARDs.<br><br>Important Considerations:<br><br>Monitoring: Regular monitoring of liver enzymes, blood counts, and lipid levels is necessary due to the potential side effects of tocilizumab. Given the patient's diabetes, careful monitoring of glycemic control is also advised, as tocilizumab can affect blood glucose levels.<br>Vaccination: Ensuring up-to-date vaccinations, especially against pneumococcus and influenza, is advisable before initiating therapy with tocilizumab, considering the patient's age and immunosuppressed state.<br>Infection Risk: As with all bDMARDs, there is an increased risk of infections. The patient and caregivers should be educated on recognizing signs of infection and seeking prompt medical attention.<br>This treatment recommendation is based on the assumption that the patient has not previously failed therapy with tocilizumab or other bDMARDs targeting similar pathways, and that there are no contraindications specific to tocilizumab for this patient. It is crucial to tailor	The recommendation for Tocilizumab is based on its efficacy in reducing symptoms and halting disease progression in RA patients who have had an inadequate response to conventional DMARDs, including methotrexate, and its potential benefit for treating RA-associated extra-articular manifestations like scleritis. Furthermore, its safety profile is manageable with careful monitoring, making it suitable for a patient with comorbid conditions such as diabetes and hypertension, considering the need to balance immunosuppression with the risk of exacerbating existing comorbidities.	An alternative therapy concept for this patient, considering the moderate disease activity of RA, previous treatments, and the presence of extra-articular manifestations such as scleritis, would be the initiation of a Janus kinase (JAK) inhibitor. Among the JAK inhibitors, Baricitinib has been approved for the treatment of moderate to severe RA in adults who have had an inadequate response to one or more DMARDs. This class of medication can be particularly useful in patients with an inadequate response to both conventional synthetic DMARDs and biologic DMARDs, offering a different mechanism of action focused on intracellular signaling pathways that contribute to the inflammation and joint damage seen in RA.<br><br>Suggested Therapy: Baricitinib<br><br>Route and Dosage: Orally, 4 mg once daily.<br>Reasons for this therapy suggestion:<br><br>Effectiveness in RA: Baricitinib has demonstrated efficacy in reducing symptoms, improving physical function, and inhibiting the progression of structural joint damage in RA patients who have not adequately responded to or cannot tolerate methotrexate or other DMARDs.<br>Ease of Administration: The oral route of administration might improve adherence and be more convenient for the patient, especially considering the patient's age and potential difficulties in managing subcutaneous injections.<br>Important Considerations:<br><br>Monitoring: Regular monitoring for signs of infection, changes in blood counts, liver enzymes, and lipid profiles is necessary due to the risk of serious infections, thrombosis, and other potential adverse effects associated with JAK inhibitors.<br>Infection Risk: Patients should be screened for latent tuberculosis and viral hepatitis before starting treatment with Baricitinib, and monitoring for the reactivation of these conditions is recommended during therapy.<br>This alternative therapy is proposed with the understanding that the patient has not previously been treated with or had an inadequate	The choice of Baricitinib as an alternative therapy is driven by its demonstrated effectiveness in controlling moderate to severe RA symptoms and its potential to improve quality of life in patients who have not responded adequately to conventional DMARDs or bDMARDs, offering a convenient oral administration route. Additionally, its mechanism of action as a JAK inhibitor addresses the inflammatory pathways directly, providing an option for patients with extra-articular manifestations and those seeking an alternative to injectable therapies.

	the choice of therapy to the patient's specific conditions, preferences, and prior treatment history, in alignment with the most current guidelines and clinical evidence.		response to JAK inhibitors and considering the patient's comorbid conditions and overall treatment goals. As with any therapy, the decision to initiate treatment with Baricitinib should be made after a thorough evaluation of the potential benefits and risks, tailored to the patient's specific clinical situation.		

CASE VIGNETTE 6:

- Age: 59 years;
- Sex: male;
- Body height in cm: 168; Body weight in kg: 75; BMI: 26.6 kg/m^2;
- Diagnosis: rheumatoid arthritis (RA);
- Disease duration: 13 years;
- Previous illnesses: arterial hypertension, coronary heart disease, post-myocardial infarction 10 years ago;
- Current medical history: The patient recently reported having a dry cough and being short of breath. The symptoms had slowly increased over 6 months. He has joint pain, partly at rest and partly on exertion, in the finger and toe joints on the left and right, but did not notice any joint swellings;
- Current clinical examination: Pressure pain in all metacarpophalangeal (MTP) joints and proximal interphalangeal (PIP) joints on both sides, no palpable swelling. Auscultation of the lungs reveals subtle crackling rales basally on both sides. The further physical examination is unremarkable;
- Swollen joint count (SJC): 0;
- Tender joint count (TJC): 15;
- Visual Analogue Scale (VAS) pain scoring: 5 (reference range 0-10);
- Anti-cyclic citrullinated peptide (CCP) titer: positive;
- Rheumatoid factor (RF): positive;
- C-reactive protein (CRP): 2.6 mg/dl (reference range < 0.5 mg/dl);
- Erythrocyte sedimentation rate (ESR): 62 mm/h (reference range 3-8 mm/h);
- Other lab results: blood counts normal, eGFR 65 ml/min (reference range ≥ 60 ml/min), liver function values normal;
- Tuberculosis elispot: negative;
- Hepatitis B serology: negative;
- Previous therapies for rheumatoid arthritis: sulfasalazine for 5 years, then methotrexate and sulfasalazine for 1 year, then methotrexate and sulfasalazine and hydroxychloroquine for 1 year. Sulfasalazine was discontinued because adverse effects (headache) had occurred.
- Medications taken regularly: HCQ 200 mg twice daily 6 days a week and once daily 1 day a week, methotrexate 20 mg subcutaneously once per week, folic acid 5 mg 24 h after methotrexate application, ASS 100 mg daily, ramipril, rosuvastatin;
- Current DAS-28: 5.2 (high disease activity);
- Current arthrosonography: not performed;
- Pulmonary function test: Progressive restrictive ventilation disorder with diffusion disorder;
- Computed tomography (CT) of the chest: interstitial reticular subpleural lung changes, marked on both lower lobes, concomitant minor alveolitis;
- Bronchoscopy and histological examination suggest organizing pneumonia; virological and microbiological examinations revealed no evidence of an infectious genesis;

TREATMENT CONCEPTS FOR CASE VIGNETTE 6:

	Therapy Concept	Reasoning behind the therapy concept	Alternative therapy concept	Reasoning behind the alternative therapy concept
**RB**	Rituximab 1g (d1, d15) i.v. Prednisolone 30mg daily (tapering: 30mg for 2 weeks, 25mg for 2 weeks, 20mg for 2 weeks, 15mg for 2 weeks, 12.5mg for 4 weeks, 10mg for 4 weeks, 7.5mg for 4 weeks, maintain 5mg daily) + Colecalciferol 1000IE daily (as long as prednisolone is taken). Continue methotrexate and folic acid unchanged, stop hydroxychloroquine (HCQ).	Rheumatoid arthritis-associated interstitial lung disease (ILD) should be treated with rituximab and will be implemented in the ACR treatment guidelines for connective tissue disease-associated interstitial lung diseases (CTD-ILD) recommendations.	Abatacept s.c. 125mg per week + prednisolone 30mg daily (tapering: 30mg for 2 weeks, 25mg for 2 weeks, 20mg for 2 weeks, 15mg for 2 weeks, 12.5mg for 4 weeks, 10mg for 4 weeks, 7.5mg for 4 weeks, maintain 5mg daily). Colecalciferol 1000IE daily (as long as prednisolone is taken). Continue methotrexate and folic acid unchanged, stop hydroxychloroquine (HCQ).	There is available evidence in registers for the efficacy of abatacept in RA-ILD.
**GPT-3.5**	Based on the patient's clinical presentation and the most current European guidelines (EULAR guidelines), a suitable therapy suggestion for this patient would be to initiate a biological disease-modifying antirheumatic drug (bDMARD) therapy. Specifically, considering the patient's comorbidities and the pulmonary involvement, a tumor necrosis factor (TNF) inhibitor would be a reasonable choice.<br><br>Therefore, the therapy concept would involve initiating adalimumab, a TNF inhibitor, at a dose of 40 mg subcutaneously every other week. This would be in addition to the current treatment regimen, which includes methotrexate and hydroxychloroquine.<br><br>It's important to closely monitor the patient for any signs of worsening pulmonary symptoms or adverse effects related to the new therapy. Additionally, regular follow-up assessments should be conducted to evaluate the response to treatment, disease activity, and any potential need for adjustments in the therapeutic approach.	The therapy suggestion of initiating adalimumab, a TNF inhibitor, is based on the patient's high disease activity as indicated by a DAS-28 score of 5.2, positive anti-CCP and rheumatoid factor, along with inadequate response to previous conventional synthetic DMARDs. Additionally, the presence of pulmonary involvement, specifically organizing pneumonia, necessitates a therapy choice with favorable pulmonary safety profiles, making TNF inhibitors a suitable option according to current EULAR guidelines.	An alternative therapy concept for this patient, considering the pulmonary involvement and previous treatment failures, would be to initiate a Janus kinase (JAK) inhibitor. Specifically, tofacitinib could be considered at a dose of 5 mg orally twice daily. This treatment would aim to address the inadequately controlled disease activity while potentially minimizing adverse effects on the lungs compared to some other DMARD options.	The alternative therapy suggestion of initiating tofacitinib, a JAK inhibitor, is based on its efficacy in treating rheumatoid arthritis, particularly in patients with inadequate responses to conventional DMARDs. Additionally, tofacitinib's oral administration and alternative mechanism of action offer a viable option, especially in patients with pulmonary involvement, potentially minimizing the risk of exacerbating lung-related symptoms compared to biologics like TNF inhibitors.
**GPT-4**	Given the patient's comprehensive medical history, current symptoms, and previous therapy for rheumatoid arthritis (RA), along with the considerations for comorbid conditions such as arterial hypertension, coronary heart disease, post-myocardial infarction, and the recent findings of organizing pneumonia, the therapeutic approach needs to be carefully chosen to minimize risks while effectively managing the RA.<br><br>Considering the most current European League Against Rheumatism (EULAR) guidelines available as of my last update in April 2023, a suitable single therapy concept for this patient would	I suggested Tocilizumab for several reasons: Firstly, it targets IL-6, a cytokine involved in the inflammatory process of RA, and has shown efficacy in patients who have not adequately responded to traditional	Given the patient's complex medical history, including rheumatoid arthritis with high disease activity, organizing pneumonia, and cardiovascular comorbidities, an alternative therapy concept that balances efficacy in controlling RA with a minimal impact on lung and heart health is crucial.<br><br>An alternative option could be Baricitinib, a Janus kinase (JAK) inhibitor. JAK inhibitors are a	Baricitinib was recommended as an alternative due to its efficacy in treating moderate to severe rheumatoid arthritis in patients who have not responded well to conventional DMARDs, providing a convenient oral administration route that may enhance adherence. Additionally, its mechanism as a JAK inhibitor allows for targeted action within the immune response pathway

	involve using a biologic disease-modifying antirheumatic drug (bDMARD) or a targeted synthetic disease-modifying antirheumatic drug (tsDMARD) that does not exacerbate the patient's lung condition or increase the risk of infections, given the organizing pneumonia and the patient's cardiovascular comorbidities.<br><br>Given the patient's previous therapy and the need for a medication that has a favorable safety profile for his lung condition, Tocilizumab might be a suitable option. Tocilizumab is an interleukin-6 (IL-6) receptor antagonist that can be used in patients who have had an inadequate response to one or more DMARDs. It has been shown to be effective in reducing the signs and symptoms of RA, improving physical function, and inhibiting the progression of structural damage in joints.<br><br>Specific Therapy Suggestion:<br>Drug Name: Tocilizumab<br>Dosage and Administration: For the treatment of RA in adults, tocilizumab can be administered either as an intravenous (IV) infusion or a subcutaneous (SC) injection. Given the patient's profile and the need to minimize hospital visits, the SC route may be preferred.<br>Subcutaneous Dosage: The standard dosage is 162 mg administered once every week.<br>It's crucial to closely monitor the patient for signs of infection, given the immunosuppressive action of tocilizumab, and to manage any potential increase in lipid levels, which could be a concern given the patient's history of coronary heart disease. Before initiating tocilizumab, it would also be prudent to re-evaluate lung condition and ensure the patient is closely monitored for any respiratory symptoms given his history of organizing pneumonia.<br><br>This therapy should be considered in the context of a comprehensive treatment plan, including ongoing assessment of cardiovascular risk, management of hypertension, and regular monitoring of the patient's lung condition. Collaboration with a pulmonologist and a cardiologist would be advisable to manage the patient's complex comorbid conditions alongside his RA.<br><br>Please note, specific recommendations can vary based on new research findings, drug availability, and individual patient factors, so it's essential to consult the most recent guidelines and collaborate with a multidisciplinary team to tailor the treatment plan to the patient's needs.	DMARDs, aligning with this patient's history of insufficient response to methotrexate and sulfasalazine. Secondly, tocilizumab's safety profile is favorable in the context of the patient's existing lung condition (organizing pneumonia) and cardiovascular comorbidities, as it does not significantly exacerbate these conditions and can be monitored and managed with regular clinical assessments.	class of medication known as targeted synthetic disease-modifying antirheumatic drugs (tsDMARDs) that have been shown to be effective in treating RA, particularly in patients who have had an inadequate response to conventional DMARDs.<br><br>Alternative Therapy Suggestion:<br>Drug Name: Baricitinib<br>Dosage and Administration: The recommended dosage for the treatment of adult patients with moderately to severely active RA who have had an inadequate response to one or more DMARDs is 4 mg once daily, taken orally.<br>Baricitinib's mechanism of action, inhibiting the activity of one or more of the Janus kinase enzymes, helps interfere with the JAK-STAT signaling pathway, which plays a critical role in the pathogenesis of RA. This option is particularly valuable for its oral administration route, offering convenience compared to injectable treatments.<br><br>This choice considers the patient's need for an effective RA management strategy that does not exacerbate existing lung conditions. However, it's important to monitor the patient for signs of infection due to the immunosuppressive effect of JAK inhibitors. Regular monitoring for potential adverse effects, including blood counts and liver enzymes, is also advised.<br><br>Choosing Baricitinib provides a balance between managing RA's symptoms and progression and considering the patient's comorbid conditions, with a convenient administration route that may improve adherence to the treatment plan.	involved in RA, potentially offering a favorable benefit-risk profile in the context of the patient's organizing pneumonia and cardiovascular comorbidities by closely monitoring and managing potential side effects.

CASE VIGNETTE 7:

- Age: 30 years;
- Sex: male;
- Body height in cm: 182; Body weight in kg: 64; BMI: 19.3 kg/m^2;
- Diagnosis: axial spondyloarthritis (axSpA);
- Disease duration: 18 months;
- Previous illnesses: none;
- Current medical history: Patient describes low back pain at night with improvement with movement. Diclofenac and ibuprofen were each taken for at least 2-4 weeks, but this only improved the pain slightly;
- Current clinical examination: Positive sacroiliac pain provocation test (Patrick's or FABER test) on the left and greater on the right, straight leg raise or Lasègue test negative, no peripheral joint swelling and no tenderness in the peripheral joints. The further physical examination is unremarkable;
- HLA-B27 status: positive;
- Visual Analogue Scale (VAS) pain scoring: 9 (reference range 0-10);
- Patient's global assessment of disease activity (PtGA): 9 (reference range 0-10);
- Bath Ankylosing Spondylitis Disease Activity Index (BASDAI): 2.8 (reference range 0-10);
- BASMI: 0;
- C-reactive protein (CRP): 0.4 mg/dl (reference range < 0.5 mg/dl);
- Erythrocyte sedimentation rate (ESR): 12 mm/h (reference range 3-8 mm/h);
- ASDAS-CRP (Ankylosing Spondylitis Disease Activity Score): 3.1 (high disease activity);
- Other lab results: blood counts normal, kidney and liver function values normal;
- Tuberculosis elispot: positive;
- Hepatitis B serology: negative;
- Previous rheumatological therapies: none (apart from NSAIDs);
- Medications taken regularly: Diclofenac 75 mg 1-0-1;
- Current X-ray findings: Chest X-ray: No effusions, no infiltrates, no post-specific changes;
- Current MRI findings: Active sacroiliitis with bilateral bone marrow edema on the left more than on the right. Three subchondral erosions of the left sacroiliac joint on the iliac side;
- Current arthrosonography: not done.

TREATMENT CONCEPTS FOR CASE VIGNETTE 7:

	Therapy Concept	Reasoning behind the therapy concept	Alternative therapy concept	Reasoning behind the alternative therapy concept
**RB**	Ixekizumab 1st injection 160mg, followed by 80mg s.c. every 4 weeks (beginning 4 weeks after initiation of isoniazide). Antibiotic therapy with isoniazide 300mg daily for 9 months and pyridoxinhydrochloride 60mg daily for 9 months. Implement physiotherapy.	SpA with high disease activity should be treated with bDMARDs or tsDMARDs. Due to latent tuberculosis antibiotics prophylaxis with isoniazide or rifampicin is necessary.	Adalimumab 40mg s.c. each other week (beginning 4 weeks after initiation of isoniazide). Isoniazide 300mg daily for 9 months and pyridoxinhydrochloride 60mg daily for 9 months. Implement physiotherapy.	Adalimumab has a higher risk of tuberculosis reactivation compared to ixekizumab, so in this case it is only the 2nd choice.
**GPT-3.5**	Based on the provided clinical information and considering the most recent European guidelines (EULAR), I would recommend initiating treatment with a biologic disease-modifying antirheumatic drug (bDMARD) due to the high disease activity and inadequate response to nonsteroidal anti-inflammatory drugs (NSAIDs). The specific drug that I would suggest is secukinumab, a interleukin-17A inhibitor.<br><br>The recommended dosage for secukinumab in axial spondyloarthritis (axSpA) according to EULAR guidelines is as follows:<br><br>Initial dose: 150 mg subcutaneously at weeks 0, 1, 2, 3, and 4.<br>Maintenance dose: 150 mg subcutaneously every 4 weeks thereafter.<br>This treatment approach aims to target the underlying inflammation associated with axSpA, particularly in patients with inadequate response to NSAIDs. Secukinumab has demonstrated efficacy in reducing disease activity and improving symptoms in patients with axSpA, including those with sacroiliitis.<br><br>It's important to closely monitor the patient for response to treatment, adverse effects, and adherence to therapy. Additionally, tuberculosis screening should be performed before initiating secukinumab due to the positive Tuberculosis elispot result.<br><br>As with any medication, the decision to start secukinumab should be made in consultation with the patient, taking into account individual preferences, comorbidities, and potential risks and benefits. Regular follow-up and assessment of disease activity are essential to optimize treatment outcomes.	Secukinumab, as an interleukin-17A inhibitor, targets the key inflammatory pathway implicated in axial spondyloarthritis (axSpA), effectively reducing disease activity and symptoms. Its efficacy has been supported by clinical trials and aligns with EULAR guidelines for treating patients with high disease activity and inadequate response to NSAIDs, offering a targeted therapeutic option with potential for significant symptom improvement.	An alternative therapy concept, considering the patient's profile and disease activity, would be to initiate treatment with a tumor necrosis factor (TNF) inhibitor. Specifically, I would recommend adalimumab, which is a TNF inhibitor commonly used in the treatment of axial spondyloarthritis (axSpA).<br><br>The recommended dosage for adalimumab in axSpA is:<br><br>Initial dose: 80 mg subcutaneously, followed by 40 mg subcutaneously every other week starting one week after the initial dose.<br>Adalimumab has demonstrated efficacy in reducing inflammation, improving symptoms, and inhibiting progression of structural damage in axSpA patients, particularly in those who have not responded adequately to NSAIDs. It is an established biologic therapy with a well-established safety profile. However, it's important to consider individual patient factors, preferences, and potential risks and benefits when selecting the appropriate treatment option.	Adalimumab, as a tumor necrosis factor (TNF) inhibitor, has been extensively studied and proven effective in reducing inflammation and symptoms in axial spondyloarthritis (axSpA), including sacroiliitis. It offers a well-established therapeutic option with demonstrated efficacy, particularly in patients with inadequate response to NSAIDs, providing an alternative biologic treatment approach to target underlying inflammation and improve disease outcomes.

**GPT-4**	Given the patient's diagnosis of axial spondyloarthritis (axSpA), their HLA-B27 positive status, high disease activity scores (VAS pain scoring: 9, PtGA: 9, ASDAS-CRP: 3.1), and the presence of active sacroiliitis with bone marrow edema as shown on MRI, along with the failure of NSAID therapy (Diclofenac and Ibuprofen) to provide sufficient relief, the treatment approach should be escalated according to the most current European League Against Rheumatism (EULAR) guidelines for the management of axial spondyloarthritis.<br><br>Considering the patient's positive Tuberculosis (TB) Elispot test, it's crucial to address latent tuberculosis before initiating biological therapy. The guidelines recommend screening for latent TB and treating it according to local protocols before starting biologic treatment, particularly tumor necrosis factor inhibitors (TNFi) or interleukin-17 inhibitors (IL-17i), to reduce the risk of reactivation.<br><br>The specific therapy suggestion, taking into account the EULAR guidelines and the patient's clinical profile, would be:<br><br>Treatment with a Tumor Necrosis Factor inhibitor (TNFi): Given the patient's high disease activity, positive MRI findings, and insufficient response to NSAIDs, a TNFi would be appropriate. However, before initiation, the patient must receive treatment for latent TB due to the positive Elispot test.<br>One TNFi option is:<br><br>Adalimumab: Adalimumab is a fully human monoclonal antibody that inhibits TNF, a key cytokine involved in the inflammatory processes of axSpA.<br>Dosage:<br><br>40 mg subcutaneously every other week<br>Before initiating Adalimumab, the patient should undergo treatment for latent tuberculosis, considering the positive TB Elispot result. The standard treatment for latent TB involves isoniazid (INH) with or without rifampin, depending on the local guidelines and the patient's liver function tests. The duration of latent TB treatment typically ranges from 3 to 9 months, depending on the specific regimen used.<br><br>It is essential to monitor the patient for signs of TB reactivation, hepatitis B reactivation (given the negative hepatitis B serology, this risk is primarily of concern in patients with prior exposure), and other potential side effects of TNFi therapy, including infections and injection site reactions. Regular follow-up appointments should be scheduled to assess the efficacy of the treatment and any adverse effects, adjusting the therapy as necessary.<br><br>This treatment recommendation is based on the information provided and the most current EULAR guidelines available up to my last update. Always consult the most recent guidelines and consider the patient's unique clinical circumstances when making treatment decisions.	I suggested Adalimumab, a Tumor Necrosis Factor inhibitor (TNFi), due to its efficacy in reducing inflammation and managing symptoms in patients with axial spondyloarthritis, especially in those with high disease activity and insufficient response to NSAIDs, as well as positive MRI findings of sacroiliitis. Before initiation, addressing latent tuberculosis is critical to prevent TB reactivation, aligning with safety protocols for TNFi treatment and EULAR guidelines for managing axSpA.	As an alternative to TNFi treatment, considering the patient's specific clinical profile and the need for an alternative therapy concept, an Interleukin-17A (IL-17A) inhibitor would be a suitable option. IL-17A inhibitors have shown effectiveness in treating axial spondyloarthritis, especially in patients who are either intolerant to, have contraindications for, or have an inadequate response to TNFi therapy.<br><br>Given the patient's clinical features and the requirements specified, the alternative recommendation would be:<br><br>Secukinumab: Secukinumab is a fully human monoclonal antibody that selectively targets IL-17A, a key cytokine involved in the pathogenesis of axial spondyloarthritis. It has been shown to reduce the signs and symptoms of the disease, improve physical function, and inhibit the progression of spinal structural damage.<br>Dosage:<br><br>150 mg administered by subcutaneous injection once weekly for the first five weeks, followed by 150 mg every four weeks<br>Before starting Secukinumab, it is essential to ensure that the patient has been adequately screened for infections, including tuberculosis, even though the patient has been identified with latent TB and should have been treated based on the previous recommendation. Monitoring for signs of infection and other potential side effects is also necessary during treatment.<br><br>This alternative therapy option provides an effective treatment pathway for axial spondyloarthritis with a different mechanism of action than TNFi, offering a potential treatment route for patients with specific needs or in cases where TNFi therapy is not suitable.	I recommended Secukinumab, an Interleukin-17A (IL-17A) inhibitor, as an alternative therapy due to its proven efficacy in reducing symptoms, inhibiting disease progression, and improving quality of life in patients with axial spondyloarthritis, particularly for those who may not respond to or tolerate TNF inhibitors. This option offers a distinct mechanism of action targeting IL-17A, a pivotal cytokine in the inflammatory process of axSpA, providing a tailored approach for managing the disease in diverse patient profiles.

CASE VIGNETTE 8:

- Age: 37 years;
- Sex: female;
- Body height in cm: 159; Body weight in kg: 82; BMI: 32.4 kg/m^2;
- Diagnosis: Psoriatic arthritis (PsA) with axial involvement;
- Disease duration: 24 months;
- Previous illnesses: obesity;
- Current medical history: The patient has had psoriasis capitis since childhood. With topical therapy, the psoriasis was little active for a long time. There is now pain in the cervical spine area, especially at rest. The patient reports no peripheral joint involvement;
- Current clinical examination: Multiple florid, very red, raised and bloody scratched lesions on the scalp. Ear canal red and scaly in the visible area. Pain when pressure is placed on the cervical spine, cervical spine rotation is only possible up to 30 degrees due to the pain; no peripheral joint swelling and no tenderness in the peripheral joints. The further physical examination is unremarkable;
- HLA-B27 status: negative;
- Visual Analogue Scale (VAS) pain scoring: 7 (reference range 0-10);
- Patient's global assessment of disease activity (PtGA): 8 (reference range 0-10);
- Bath Ankylosing Spondylitis Disease Activity Index (BASDAI): 3.8 (reference range 0-10);
- C-reactive protein (CRP): 2.1 mg/dl (reference range < 0.5 mg/dl);
- Erythrocyte sedimentation rate (ESR): 38 mm/h (reference range 3-8 mm/h);
- ASDAS-CRP (Ankylosing Spondylitis Disease Activity Score): 3.5 (high disease activity);
- DAPSA (Disease Activity in PSoriatic Arthritis) score: 20 (high disease activity);
- Psoriasis Area Severity Index (PASI) score: 5.5, moderate disease activity (reference range 0-72);
- Other lab results: blood counts normal, kidney and liver function values normal;
- Tuberculosis elispot: negative;
- Hepatitis B serology: negative;
- Previous therapies for SpA: none;
- Medications taken regularly: vitamin D, magnesium;
- Current X-ray findings: Chest: No effusions, no infiltrates, no post-specific changes;
- Current MRI findings: Active inflammation of the facet joints of cervical vertebrae 3-7 and the anterior longitudinal ligament. Signs of synovitis in the sternoclavicular joints on both sides with slight joint effusion;
- Current arthrosonography: not done.

TREATMENT CONCEPTS FOR CASE VIGNETTE 8:

	Therapy Concept	Reasoning behind the therapy concept	Alternative therapy concept	Reasoning behind the alternative therapy concept
**RB**	Secukinumab 300mg s.c. in weeks 0, 1, 2, 3, and 4, then 300mg every 4 weeks. Ibuprofen 600mg as needed (max. 2400mg daily). Implement physiotherapy.	The patient has active skin and active axial involvement. Due to moderate Psoriasis activity 300mg dosage should be applied.	Ixekizumab 1st injection 160mg, followed by 80mg s.c. In weeks 0, 2, 4, 6, 8, 10, and 12, then 80mg every 4 weeks. Diclofenac 50mg as needed (maximum 150 mg daily). Implement physiotherapy.	Secukinumab and ixekizumab might be similar in efficacy and adverse events. Different pain killers might need to be tested concerning efficacy.
**GPT-3.5**	Based on the provided clinical information and the current European guidelines (EULAR guidelines), a suitable therapy concept for this patient with psoriatic arthritis (PsA) with axial involvement would be initiating treatment with a biologic disease-modifying antirheumatic drug (bDMARD). Specifically, considering the high disease activity and axial involvement, along with the presence of psoriasis, the recommendation would be to start treatment with a tumor necrosis factor (TNF) inhibitor.<br><br>The specific drug suggested would be Adalimumab, administered at an initial dose of 80 mg subcutaneously, followed by 40 mg subcutaneously every other week. Adalimumab is a TNF inhibitor that has demonstrated efficacy in treating both peripheral arthritis and axial involvement in PsA, as well as improving skin manifestations of psoriasis.<br><br>Additionally, the patient may continue with their current medications of vitamin D and magnesium. Regular monitoring of disease activity, including assessment of joint symptoms, skin involvement, and inflammatory markers, should be performed to evaluate treatment response and adjust therapy as necessary.<br><br>It's important to note that treatment decisions should always be made in consultation with a healthcare professional, considering the individual patient's preferences and any specific contraindications or concerns.	Adalimumab, a TNF inhibitor, is recommended as a first-line biologic therapy for psoriatic arthritis with axial involvement due to its proven efficacy in reducing disease activity, improving axial symptoms, and addressing both joint and skin manifestations, thus providing comprehensive treatment for this patient's multi-faceted disease presentation. Additionally, adalimumab's favorable safety profile and convenient dosing regimen make it a suitable choice for long-term management in this patient population.	An alternative therapy concept, especially if there are concerns about initiating a TNF inhibitor, could involve starting treatment with an interleukin-17 (IL-17) inhibitor. Specifically, Secukinumab would be a suitable alternative.<br><br>Secukinumab is a monoclonal antibody targeting IL-17A, which has demonstrated efficacy in treating both peripheral arthritis and axial involvement in psoriatic arthritis, as well as improving skin symptoms. The recommended dosage for secukinumab would be an initial loading dose of 150 mg subcutaneously, followed by maintenance dosing of 150 mg every four weeks. This alternative provides another effective option for addressing the patient's high disease activity and axial involvement while offering a different mechanism of action compared to TNF inhibitors.	Secukinumab, as an IL-17 inhibitor, offers a targeted approach to addressing the inflammatory pathways involved in psoriatic arthritis, including both peripheral and axial manifestations, while also addressing skin involvement. Its efficacy and safety profile make it a viable alternative for patients who may have contraindications or prefer an alternative to TNF inhibitors.

**GPT-4**	Based on the detailed patient profile provided, the most appropriate therapy according to the latest European League Against Rheumatism (EULAR) guidelines for the management of psoriatic arthritis (PsA) with axial involvement would focus on addressing both the axial symptoms and the skin manifestations effectively, considering the patient's obesity and the absence of peripheral joint involvement but significant axial disease and psoriasis.<br><br>Based on the details provided and considering the most current European League Against Rheumatism (EULAR) guidelines for the management of psoriatic arthritis (PsA) with axial involvement, a specific therapy suggestion for this 37-year-old female patient with high disease activity and no previous systemic therapies for PsA would be:<br><br>Therapy Suggestion: Ixekizumab<br><br>Rationale: Ixekizumab, an IL-17A inhibitor, is recommended for patients with PsA who have axial involvement, especially when there's also significant skin involvement, as is the case here. This recommendation is based on the efficacy of IL-17A inhibitors in controlling both the axial and skin manifestations of psoriatic disease. Given the patient's high disease activity indicated by the ASDAS-CRP and DAPSA scores, moderate psoriasis activity reflected by the PASI score, and the presence of axial involvement without peripheral joint involvement, an IL-17A inhibitor like Ixekizumab would be appropriate.Dosage and Administration: The initial dose is 160 mg (as two 80 mg injections) at week 0, followed by 80 mg every 4 weeks. This dosing regimen is based on the drug's prescribing information and is adjusted for subcutaneous administration.<br><br>Monitoring and Precautions: Regular monitoring for signs of infection, potential allergic reactions, and monitoring of liver function tests is recommended due to the immunosuppressive action of biologic therapies. Despite the patient's obesity, dosage adjustment based on body weight is not required for ixekizumab, simplifying the treatment regimen.<br><br>Justification: This recommendation aligns with the EULAR guidelines, which prioritize treatment efficacy across all domains of PsA, including axial disease, psoriasis, enthesitis, and dactylitis, while also considering patient safety and comorbidities. The choice of an IL-17A inhibitor over a TNF inhibitor or other biologic therapies (e.g., IL-12/23 inhibitors) is influenced by the specific clinical features of the case, including significant axial involvement and moderate to severe psoriasis, for which IL-17A inhibitors have shown strong efficacy.This therapy suggestion is contingent upon the latest EULAR guidelines and available clinical evidence. It is crucial to tailor the treatment plan to the individual patient's needs, preferences, and any emerging evidence or guidelines. Continuous assessment of treatment efficacy and safety is essential, with adjustments made as necessary to optimize patient outcomes.	Ixekizumab is chosen due to its proven efficacy in treating both the axial symptoms of psoriatic arthritis and the skin manifestations of psoriasis, addressing the patient's primary concerns effectively. This choice is further supported by its safety profile and the patient's specific clinical presentation, including significant axial involvement without peripheral joint swelling, high disease activity, and moderate psoriasis severity, making it an ideal single therapy option under the EULAR guidelines.	As an alternative therapy concept for this patient with psoriatic arthritis (PsA) with axial involvement, considering the EULAR guidelines, another effective option would be:<br><br>Therapy Suggestion: Secukinumab<br><br>Rationale: Secukinumab, another IL-17A inhibitor, has been shown to be effective in treating both the axial and dermatological manifestations of PsA, similar to Ixekizumab. Given the patient's specific condition, including axial involvement, high disease activity, and moderate psoriasis severity, Secukinumab offers a comprehensive approach to managing the disease's multifaceted nature.<br><br>Dosage and Administration: The recommended starting dosage for Secukinumab in PsA with axial involvement is 150 mg by subcutaneous injection with loading doses at weeks 0, 1, 2, 3, and 4, followed by monthly maintenance dosing. For patients with concomitant moderate to severe plaque psoriasis or those who do not respond adequately to the 150 mg dose, an increased dose of 300 mg may be considered.<br><br>Monitoring and Precautions: Similar to Ixekizumab, monitoring for infections, signs of allergic reactions, and routine blood work to assess liver function is advised due to the immunosuppressive nature of the treatment. Secukinumab's administration does not require adjustment for body weight, simplifying treatment for patients of various sizes.<br><br>This alternative is chosen for its similar mode of action to Ixekizumab, targeting IL-17A to reduce inflammation and provide relief from both joint and skin symptoms associated with PsA. Secukinumab's efficacy in axial symptoms specifically makes it a strong alternative for patients with significant spinal involvement, as is the case here.	Secukinumab is selected as an alternative due to its targeted action on IL-17A, effectively addressing the axial and cutaneous manifestations of PsA, which aligns with the patient's clinical presentation and therapeutic needs. Its proven efficacy in improving axial symptoms and moderate to severe psoriasis, alongside a favorable safety profile, makes it a compelling alternative option for comprehensive disease management in line with EULAR recommendations.

CASE VIGNETTE 9:

- Age: 22 years;
- Sex: female;
- Body height in cm: 165; Body weight in kg: 55; BMI: 20.2 kg/m^2;
- Diagnosis: axial Spondyloarthritis (axSpA);
- Disease duration: 6 months;
- Previous illnesses: gastrointestinal ulcer occurred when taking NSAIDs regularly after a radius fracture 2 years ago;
- Current medical history: Back pain in the low back occurred for the first time 6 months ago. The pain is persistent since then, at rest and also when moving. Morning stiffness is 30 minutes. A painkiller (Tilidine) was ineffective. There is also pain and swelling in both knee joints and pain, particularly when running, in the left heel. Both the patient's brother and father suffer from axial spondyloarthritis;
- Current clinical examination: Tenderness and swelling in both knee joints. Overheating and thickening of the left achilles tendon. Positive sacroiliac pain provocation test (Patrick's or FABER test) on both sides. The further physical examination is unremarkable;
- HLA-B27 status: positive;
- Visual Analogue Scale (VAS) pain scoring: 8 (reference range 0-10);
- Patient's global assessment of disease activity (PtGA): 8 (reference range 0-10);
- Bath Ankylosing Spondylitis Disease Activity Index (BASDAI): 6.4 (reference range 0-10);
- C-reactive protein (CRP): 0.7 mg/dl (reference range < 0.5 mg/dl);
- Erythrocyte sedimentation rate (ESR): 23 mm/h (reference range 3-8 mm/h);
- ASDAS-CRP (Ankylosing Spondylitis Disease Activity Score): 3.8 (very high disease activity);
- Other lab results: blood counts normal, kidney and liver function values normal;
- Tuberculosis elispot: negative;
- Hepatitis B serology: negative;
- Previous therapies for SpA: none;
- Medications taken regularly: vitamin D, magnesium;
- Current X-ray findings: Chest: No effusions, no infiltrates, no post-specific changes;
- Current MRI findings: Active sacroiliitis with bilateral bone marrow edema. No erosions;
- Current arthrosonography: Left knee: Suprapatellar capsule distension C with synovial proliferation, hyperperfusion III°. Cartilage over the femoral condyles was sonographically normal.

TREATMENT CONCEPTS FOR CASE VIGNETTE 9:

	Therapy Concept	Reasoning behind the therapy concept	Alternative therapy concept	Reasoning behind the alternative therapy concept
**RB**	Adalimumab 40mg s.c. each other week. Implement physiotherapy. If adalimumab is not sufficient for peripheral arthritis methotrexate can be added to therapy regimen after 12 weeks.	Active axial and peripheral spondyloarthritis with contraindication for NSAIDs.	Ixekizumab 1st injection 160mg, followed by 80mg s.c. every 4 weeks. Implement physiotherapy. If ixekizumab is not sufficient for peripheral arthritis sulfasalazine can be added to therapy regimen after 12 weeks.	Active axial and peripheral spondyloarthritis with contraindication for NSAIDs.
**GPT-3.5**	Based on the provided clinical information and the most current European guidelines for the management of axial spondyloarthritis (axSpA), a recommended therapy concept for this patient would involve initiating treatment with a biologic disease-modifying antirheumatic drug (bDMARD).<br><br>Given the high disease activity, positive HLA-B27 status, and the presence of active sacroiliitis with bilateral bone marrow edema on MRI, along with failed response to painkillers and previous gastrointestinal ulcer with NSAID use, a tumor necrosis factor-alpha (TNF-alpha) inhibitor would be an appropriate choice for this patient.<br><br>Specifically, I would recommend initiating treatment with Adalimumab, which is a TNF-alpha inhibitor. The recommended dosage for Adalimumab in axial spondyloarthritis would be 40 mg subcutaneously every other week.<br><br>This therapy concept addresses the underlying inflammation, reduces disease activity, and aims to improve symptoms and function in this young patient with axSpA while considering her previous medical history and current clinical status. Additionally, regular monitoring for treatment response and potential adverse effects is crucial according to EULAR guidelines.	The recommendation of Adalimumab, a TNF-alpha inhibitor, is supported by its effectiveness in reducing disease activity and improving symptoms in axial spondyloarthritis, particularly in patients with high disease activity and sacroiliitis evident on MRI. Additionally, considering the patient's history of gastrointestinal ulcer and lack of response to NSAIDs, a biologic therapy like Adalimumab offers a safer alternative with a lower risk of gastrointestinal adverse effects.	An alternative therapy concept, considering the patient's high disease activity, positive HLA-B27 status, and sacroiliitis evident on MRI, would involve initiating treatment with a Janus kinase (JAK) inhibitor.<br><br>Specifically, I would recommend initiating treatment with Tofacitinib, a JAK inhibitor. The recommended dosage for Tofacitinib in axial spondyloarthritis is 5 mg orally twice daily.<br><br>This therapy concept offers a targeted approach to modulating inflammation by inhibiting JAK pathways, which are involved in the pathogenesis of axSpA. Tofacitinib provides an alternative to TNF-alpha inhibitors and may be particularly suitable for patients with contraindications or intolerance to biologic therapies.	The recommendation of Tofacitinib, a JAK inhibitor, is supported by its efficacy in reducing disease activity and improving symptoms in axial spondyloarthritis, particularly in patients with inadequate response or intolerance to TNF-alpha inhibitors. Additionally, as an oral therapy, Tofacitinib offers convenience and may be preferable for patients who prefer oral medications or have concerns about injections.

**GPT-4**	Based on the patient's profile and the details provided, considering the most current European League Against Rheumatism (EULAR) guidelines for the management of axial Spondyloarthritis (axSpA), a single therapy concept can be proposed. It is essential to note that any treatment plan should be discussed with and monitored by a healthcare professional experienced in managing axSpA.<br><br>Given the patient's history of gastrointestinal ulcer related to NSAID use, the presence of very high disease activity as indicated by ASDAS-CRP, and the ineffective response to non-steroidal anti-inflammatory drugs (NSAIDs) as well as a contraindication due to previous adverse effects, the use of a biologic therapy is recommended.<br><br>Considering the patient's positive HLA-B27 status, family history of axSpA, and MRI findings of active sacroiliitis without erosions, along with the ineffectiveness and contraindications of NSAIDs, the following therapy is suggested:<br><br>Therapy Suggestion: Initiation of a Tumor Necrosis Factor inhibitor (TNFi), specifically Adalimumab.<br><br>Drug: Adalimumab<br>Dosage: 40 mg subcutaneously every other week Adalimumab is a fully human monoclonal antibody that inhibits TNF-alpha, a key cytokine involved in the inflammation process of axSpA. This treatment option is aligned with the EULAR recommendations for patients with axSpA who have active disease despite conventional treatment and are not suitable for NSAID therapy due to contraindications or intolerance.<br><br>It is important to ensure that the patient is screened for latent tuberculosis and hepatitis B before initiating biologic therapy, even though the provided information indicates a negative tuberculosis test and hepatitis B serology. Regular monitoring for potential side effects, disease activity, and adjustment of therapy as needed is also essential.<br><br>Before proceeding with any treatment, it's crucial to have a detailed discussion with the patient regarding the benefits and potential risks associated with biologic therapy, including the risk of infections. The decision to initiate a specific treatment should be made in the context of a shared decision-making process between the healthcare provider and the patient, taking into account the patient's preferences, comorbidities, and risk factors.	The choice of Adalimumab for this patient is based on its efficacy in reducing inflammation and managing symptoms in axial Spondyloarthritis, especially in patients with contraindications to NSAIDs due to gastrointestinal issues, and those with high disease activity and evidence of inflammation on MRI. Additionally, biologic therapy with a TNF inhibitor like Adalimumab is recommended by EULAR guidelines for patients with axSpA who have not responded to first-line NSAIDs or for whom these are contraindicated, aligning with the patient's clinical profile and ensuring a targeted approach to reduce disease activity and improve quality of life.	For an alternative therapy concept, considering the need to avoid NSAIDs due to the patient's gastrointestinal history and the necessity for effective management of very high disease activity in axial Spondyloarthritis, an IL-17 inhibitor would be an appropriate choice.<br><br>Secukinumab is recommended as an alternative. It is a fully human monoclonal antibody that selectively targets IL-17A, a key cytokine involved in the pathogenesis of spondyloarthritis, leading to reduced inflammation and progression of the disease.<br><br>Dosage: The recommended dose for adult patients with axial spondyloarthritis is 150 mg by subcutaneous injection with loading doses at weeks 0, 1, 2, 3, and 4, followed by monthly maintenance dosing. Depending on the clinical response and if the patient has active psoriatic arthritis or psoriasis, the dose may be increased to 300 mg.<br><br>Secukinumab offers a targeted approach to manage the inflammatory process in axSpA and has been shown to improve symptoms, function, and quality of life in patients with axSpA. This option is particularly relevant for patients who have contraindications or have failed to respond to TNF inhibitors, providing a beneficial alternative that addresses the underlying pathophysiology of the disease.	Secukinumab, an IL-17 inhibitor, was chosen as an alternative due to its targeted mechanism of action against IL-17A, a cytokine pivotal in the inflammation and progression of axial Spondyloarthritis, making it effective for patients with high disease activity and specific inflammatory pathways. This therapy is particularly suitable for patients needing an alternative to TNF inhibitors, offering a distinct and effective approach to managing symptoms and improving quality of life while minimizing gastrointestinal risks associated with NSAIDs.

CASE VIGNETTE 10:

- Age: 47 years;
- Sex: male;
- Body height in cm: 182; Body weight in kg: 101; BMI: 30.5 kg/m^2;
- Diagnosis: Psoriatic arthritis
- Disease duration: 12 years;
- Previous illnesses: none;
- Current medical history: The patient has a long-standing history of florid psoriatic arthritis. After initial treatment with sulfasalazine and methotrexate for almost 11 years, the therapy was switched to ustekinumab 45 mg s.c. every 3 months 7 months ago for persistent polyarthritis (both knee joints, both elbow joints, enthesitis of the left Achilles tendon) and florid psoriatic lesions. The skin has improved significantly under ustekinumab and is currently well controlled under additional topical therapy. The joints have also improved significantly. Currently, there is still florid arthritis in the right knee joint, which limits the patient's function considerably. Walking is only possible with crutches. Due to lack of exercise, the patient has gained 7 kg in weight since the last presentation 5 months ago. The patient has never had a joint injection before;
- Current clinical examination: Swelling, pressure pain and overheating of the right knee. Residual, healing psoriatic plaque of the right elbow. The rest of the clinical examination is unremarkable. There is no pressure pain and no swelling of other joints. The further physical examination is unremarkable;
- HLA-B27 status: negative;
- Visual Analogue Scale (VAS) pain scoring: 9 (reference range 0-10);
- Patient's global assessment of disease activity (PtGA): 5 (reference range 0-10);
- DAPSA (Disease Activity in PSoriatic Arthritis) score: 18 (high disease activity);
- Psoriasis Area Severity Index (PASI) score: 0.6, low disease activity (reference range 0-72);
- C-reactive protein (CRP): 1.7 mg/dl (reference range < 0.5 mg/dl);
- Erythrocyte sedimentation rate (ESR): 39 mm/h (3-8 mm/h);
- Other lab results: blood counts normal, kidney and liver function values normal
- Tuberculosis elispot: negative
- Hepatitis B serology: negative
- Previous therapies for PsA: sulfasalazine for 5 years, then methotrexate and sulfasalazine in combination for another 6 years;
- Medications taken regularly: ustekinumab 45 mg subcutaneously every 3 months, ibuprofen 600 mg 3 times per day, pantoprazole 40 mg 1-0-0;
- X-ray findings: Chest (2 years ago): No effusions, no infiltrates, no post-specific changes;
- Current MRI findings: not done;
- Current arthrosonography: Right knee: Osseous structures unremarkable. Suprapatellar capsular distension with synovial proliferation, hyperperfusion III°. Cartilage above the femoral condyles unremarkable sonographically.

TREATMENT CONCEPTS FOR CASE VIGNETTE 10:

	Therapy Concept	Reasoning behind the therapy concept	Alternative therapy concept	Reasoning behind the alternative therapy concept
**RB**	Increase ustekinumab dose to 90mg s.c. every 12 weeks. Additionally, intraarticular injection of triamcinolonacetonid 20mg can be performed.	Body weight has increased over 100kg, so ustekinumab might be underdosed.	Stop ustekinumab. Begin guselkumab 100mg s.c. every 4 weeks.	Another IL-23 inhibitor might also be efficatious for skin and articular inflammation.
**GPT-3.5**	Based on the provided information and considering the EULAR guidelines for the treatment of psoriatic arthritis, a suitable therapy suggestion for this patient could be to initiate treatment with a tumor necrosis factor (TNF) inhibitor. Specifically, adalimumab could be considered as the next step in therapy.<br><br>Here's the therapy concept:<br><br>Drug: Adalimumab<br>Dosage: Initiate with a loading dose of 80 mg subcutaneously, followed by 40 mg every other week thereafter.<br>This recommendation aligns with the EULAR recommendations for the management of psoriatic arthritis, particularly in cases of inadequate response or intolerance to conventional synthetic disease-modifying antirheumatic drugs (csDMARDs) and in patients with active disease. Adalimumab is a TNF inhibitor that has demonstrated efficacy in improving signs and symptoms of psoriatic arthritis, including joint manifestations and skin involvement.<br><br>Additionally, since the patient is already on ustekinumab and still experiences significant arthritis in the right knee joint, switching to a different class of biologic agent such as a TNF inhibitor like adalimumab may provide additional therapeutic benefit. Furthermore, the patient's high disease activity, as evidenced by the elevated DAPSA score and significant pain, justifies the initiation of a biologic agent.<br><br>It's important to monitor the patient closely for both efficacy and safety, including assessment of disease activity, joint symptoms, skin involvement, and laboratory parameters. Regular follow-up visits will allow for adjustments in treatment as needed to achieve optimal disease control and improve the patient's functional status.	The recommendation for adalimumab stems from its well-established efficacy in treating psoriatic arthritis, particularly in patients who have not adequately responded to previous treatments such as csDMARDs. Its mechanism of action as a TNF inhibitor targets the inflammatory cascade underlying the disease, addressing both joint symptoms and skin manifestations, thus potentially providing significant relief for the patient's persistent arthritis and enhancing overall disease control.	An alternative therapy concept for this patient could involve switching to a different class of biologic agent targeting interleukin-17 (IL-17) pathway. Specifically, secukinumab could be considered.<br>Here's the alternative therapy concept:<br>Drug: Secukinumab<br>Dosage: Initiate with a loading dose of 150 mg subcutaneously, followed by 150 mg every 4 weeks thereafter.<br>This recommendation is based on the patient's persistent arthritis despite ustekinumab therapy, suggesting potential inadequacy of IL-12/IL-23 inhibition alone in controlling his disease. Secukinumab, as an IL-17A inhibitor, offers an alternative mechanism of action that may provide improved disease control, particularly in patients with active psoriatic arthritis.	The recommendation for secukinumab is based on its efficacy in targeting the IL-17 pathway, which plays a crucial role in the pathogenesis of psoriatic arthritis. Given the patient's inadequate response to ustekinumab, which targets the IL-12/IL-23 pathway, switching to a different class of biologic agent such as an IL-17 inhibitor like secukinumab offers a targeted approach to address the underlying inflammation and potentially achieve better disease control.

**GPT-4**	Given the detailed case of a 47-year-old male with a long-standing history of Psoriatic Arthritis (PsA), showing significant improvement under ustekinumab treatment for skin lesions and joint symptoms, except for persisting florid arthritis in the right knee joint, a tailored therapy adjustment is needed. Considering the most current European League Against Rheumatism (EULAR) guidelines for the management of Psoriatic Arthritis, which prioritize both symptom control and the prevention of further joint damage, the following specific therapy suggestion is made:<br><br>Therapy Suggestion: Initiate treatment with an interleukin-17A (IL-17A) inhibitor, Secukinumab.<br><br>Rationale: Since the patient has shown partial response to ustekinumab, an IL-12/23 inhibitor, but still has active arthritis in the right knee that significantly limits function, switching to or adding a biologic from a different class could be beneficial. IL-17A inhibitors have been shown to be effective in treating both the articular and dermatological manifestations of PsA. Secukinumab, specifically, has demonstrated efficacy in reducing the signs and symptoms of PsA, improving physical function, and inhibiting the progression of peripheral joint damage.<br><br>Specific Dosage and Administration: The recommended dose of Secukinumab for Psoriatic Arthritis is 150 mg by subcutaneous injection with loading doses at weeks 0, 1, 2, 3, and 4, followed by monthly maintenance dosing. Considering the patient's significant knee involvement and previous biologic therapy, a higher dose of 300 mg may be considered based on clinical judgment and patient response.<br><br>Monitoring and Follow-up: Regular monitoring for therapeutic response and adverse effects is essential. Follow-up assessments should include clinical evaluation of joint symptoms, Psoriasis Area Severity Index (PASI) for skin lesions, and laboratory tests for inflammation markers (CRP, ESR). Adjustments to therapy should be made based on these assessments and patient feedback.<br><br>Note: Before initiating Secukinumab, it is crucial to screen for tuberculosis (TB) and other infections due to the increased risk of infections with biologic treatments. Although the patient's TB screening is negative, monitoring for signs of infection during treatment is important.<br><br>This recommendation aligns with the EULAR guidelines, emphasizing individualized treatment approaches based on disease activity, clinical features, and previous therapy responses. It's important to discuss this therapeutic option with the patient, considering potential benefits, risks, and the patient's preferences and lifestyle.	The choice of Secukinumab is based on its efficacy in targeting IL-17A, a key cytokine involved in the pathogenesis of Psoriatic Arthritis, particularly in patients who have an inadequate response to previous treatments like ustekinumab. Its ability to significantly reduce symptoms, improve physical function, and inhibit joint damage progression in PsA, coupled with the patient's need for a more effective control of joint inflammation and preservation of joint function, makes it a suitable therapeutic option according to the latest EULAR guidelines.	As an alternative therapy concept for a patient with Psoriatic Arthritis (PsA) who has had an inadequate response to ustekinumab and traditional disease-modifying antirheumatic drugs (DMARDs), the next recommendation would be to initiate treatment with a Phosphodiesterase 4 (PDE4) inhibitor, specifically:<br><br>Alternative Therapy Suggestion: Apremilast<br><br>Rationale: Apremilast works by inhibiting PDE4, leading to increased intracellular cAMP levels, which helps to modulate the expression of inflammatory cytokines. It is effective in reducing the signs and symptoms of PsA, including joint pain, swelling, and skin lesions, and has the advantage of being an oral medication, which might be preferred by some patients over injectable treatments.<br><br>Specific Dosage and Administration: The recommended starting dosage of Apremilast is 10 mg orally once daily in the morning on the first day, with titration over the first week to the maintenance dose: Day 1: 10 mg in the morning; Day 2: 10 mg twice daily; Day 3: 10 mg in the morning and 20 mg in the evening; Day 4: 20 mg twice daily; Day 5: 20 mg in the morning and 30 mg in the evening; thereafter, 30 mg twice daily.<br><br>Monitoring and Follow-up: Patients should be monitored for side effects such as diarrhea, nausea, and headache, which are commonly reported but tend to decrease over time. Additionally, monitoring for weight loss and depressive symptoms is recommended, as these are potential side effects of Apremilast.<br><br>Apremilast offers a different mechanism of action compared to biologic therapies and may be particularly appealing for patients seeking an oral medication. It provides a beneficial option for managing both joint and skin manifestations of PsA without the need for routine laboratory monitoring, aligning with patient preferences and clinical needs.	The recommendation of Apremilast as an alternative therapy is due to its unique mechanism of action as a PDE4 inhibitor, offering an oral administration route that may be preferred by patients averse to injections. Its efficacy in reducing inflammatory symptoms of Psoriatic Arthritis and psoriasis, with a safety profile that allows for use without extensive laboratory monitoring, makes it an attractive option for patients with inadequate responses to or intolerances of biologic therapies and traditional DMARDs.

:

CASE VIGNETTE 11:

- Age: 58 years;
- Sex: male;
- Body height in cm: 175; Body weight in kg: 81; BMI: 26.4 kg/m^2;
- Diagnosis: Psoriatic arthritis (PsA)
- Disease duration: 35 years
- Previous illnesses: Hashimoto's thyroiditis, degenerative spinal changes of the cervical spine, thoracic spine and lumbar spine
- Current medical history: The patient has long-standing psoriatic arthritis with peripheral joint and tendon involvement, recurrent uveitis and psoriasis vulgaris and pustulosa and nail psoriasis. Many therapy attempts in the past have been frustrating. The list of all psoriatic arthritis therapies applied to date and the reasons for discontinuation in each case are listed below.
  Today, the patient describes increasing psoriasis gluteal, on the extensor sides of the knees, the elbows and intraauricular psoriasis. He had pain in the index finger of his right hand, which was also swollen, and in both knee joints. He is not prone to infections and his weight is stable. Otherwise, he had no other complaints. Inflammatory back pain is denied.
- Current clinical examination: Dactylitis of the index finger of the right hand with marked pressure pain of the finger, overheating and swelling of both knee joints, highly florid and extensive psoriasis lesions gluteal, extensor on the knee joints, the elbows. Intraauricular scaling and redness on both sides. The further physical examination is unremarkable;
- HLA-B27 status: positive;
- Visual Analogue Scale (VAS) pain scoring: 9 (reference range 0-10);
- Patient's global assessment of disease activity (PtGA): 8 (reference range 0-10);
- DAPSA (Disease Activity in PSoriatic Arthritis) score: 29 (high disease activity);
- Psoriasis Area Severity Index (PASI) score: 16 (high disease activity);
- C-reactive protein (CRP): 4.1 mg/dl (reference range < 0.5 mg/dl);
- Erythrocyte sedimentation rate (ESR): 23 mm/h (3-8 mm/h);
- Other lab results: blood counts normal, kidney and liver function values normal;
- Tuberculosis elispot: negative;
- Hepatitis B serology: negative;
- Previous therapies for PsA:
  - sulfasalazine for 3.5 years in case of recurrence, discontinued in case of remission, 2-year treatment break
  - methotrexate (MTX) 15 mg subcutaneously once a week for psoriasis flare-up and polyarthritis, 1 year of MTX monotherapy
  - sulfasalazine+ MTX 20 mg subcutaneously once a week for arthritis over 3 months, increasing liver enzymes, re-exposure with MTX 10 mg subcutaneously once a week , again stopped due to elevated liver enzymes
  - leflunomide 20 mg daily for 9 months, improvement in joints, worsening of psoriasis
  - adalimumab over 1 month, insufficiently effective (skin psoriasis, daktylitis and enthesitis)
  - infliximab for 1 year, initially good disease control, then secondary failure (severe skin and nail psoriasis);
  - etanercept for 4 months, primary efficacy failure (skin and nail psoriasis, polyarthritis, enthesitis);
  - ustekinumab +leflunomide for 6 months, ineffective for arthritis and first occurrence of anterior uveitis on the right;

- golimumab and leflunomide for 5 months, ineffective for skin and nail psoriasis, polyarthritis, enthesitis;
- certolizumab pegol + leflunomide for 3 years, good disease control temporarily, then secondary failure, polyarthritis and mild uveitis on the right;
- secukinumab 150 mg and then 300 mg for 12 months, partially effective, meanwhile 3 mild uveitides, additional topical steroid therapy necessary, polyarthritis insufficiently controlled;
- adalimumab for 4 months, ineffective for skin psoriasis (PASI 22);
- apremilast+ leflunomide for 1 month, partially effective, but apremilast poorly tolerated with initial manifestation of a depressive episode, leflunomide washed out due to suspected allergic reaction;
- abatacept for 2 years, partially effective, finally secondary failure of efficacy and severe relapse with arthritis and severe skin psoriasis (PASI 24);
- secukinumab for 2 years, partially effective, skin psoriasis completely regressed, finally secondary failure of efficacy and severe relapse with arthritis and severe uveitis;
- ixekizumab for 6 months, ineffective for arthritis and uveitis;

- Medications taken regularly: upadacitinib 15 mg daily (started 8 months ago) vitamin D, magnesium;
- X-ray findings: Chest (1 year ago): No effusions, no infiltrates, no post-specific changes;
- Current MRI findings: Not done.
- Current arthrosonography: Right knee: Osseous structures irregular. Suprapatellar capsular distension with synovial proliferation, hyperperfusion III°. Medial cartilage above the femoral condyles thinned; Left knee: Osseous structures unremarkable. Suprapatellar capsular distension with synovial proliferation, hyperperfusion III°. Cartilage above the femoral condyles unremarkable sonographically.

TREATMENT CONCEPTS FOR CASE VIGNETTE 11:

	Therapy Concept	Reasoning behind the therapy concept	Alternative therapy concept	Reasoning behind the alternative therapy concept
**RB**	Bimekizumab 320mg s.c. in weeks 0, 4, 8, 12, 16, then 320 mg every 8 weeks. Ibuprofen 600mg as needed (maximum 2400mg daily). Stop upadacitinib. If recurrent uveitis appears, ciclosporine A can be coadministrated to bimekizumab.	Bimkizumab might not suppress uveitis activity, but might address arthritis and skin.	Tofacitinib 11 mg once daily. Ibuprofen 600mg as needed (maximum 2400mg daily). Stop upadacitinib.	Many DMARDs have failed, meaning that only 2 alternatives (apart from bimekizumab) for in-label therapy are left. Tofacitinib or guselkumab. Tofacitinib might work faster than guselkumab.
**GPT-3.5**	Based on the patient's history of psoriatic arthritis (PsA) and the failure of multiple previous therapies, including conventional synthetic DMARDs (disease-modifying antirheumatic drugs) and multiple biologic agents, as well as their current high disease activity status, the most appropriate therapy concept according to the European guidelines (EULAR guidelines) would likely involve a targeted biologic agent.<br><br>Considering the patient's history of inadequate response or adverse events with several biologic agents, the EULAR recommendations suggest the consideration of newer targeted therapies with different mechanisms of action.<br><br>For this patient, given the lack of response to previous TNF inhibitors (e.g., adalimumab, infliximab, etanercept, golimumab, certolizumab pegol), IL-17 inhibitors (e.g., secukinumab, ixekizumab), and IL-12/23 inhibitors (e.g., ustekinumab), as well as their positive HLA-B27 status, a promising therapeutic option would be a Janus kinase (JAK) inhibitor.<br><br>Therefore, for this patient, I would suggest:<br><br>Therapy Concept:<br><br>Drug: Tofacitinib<br>Dosage: Tofacitinib 5 mg twice daily (BID) initially, with possible escalation to 10 mg BID based on response and tolerability.<br>Tofacitinib is a JAK inhibitor that has shown efficacy in the treatment of PsA and other inflammatory conditions. Its mechanism of action differs from TNF inhibitors and other biologic agents, which may provide a novel approach for this patient who has failed multiple previous therapies.<br><br>Before initiating tofacitinib, it is crucial to assess the patient's current tuberculosis (TB) status and screen for other potential infections, as JAK inhibitors are associated with an increased risk of infections. Additionally, monitoring for liver function tests, lipid levels, and blood cell counts is recommended during treatment with tofacitinib.<br><br>As with any medication, the decision to initiate tofacitinib should involve a discussion with the patient regarding potential risks and benefits, considering their medical history and treatment	The recommendation of tofacitinib for this patient is based on their extensive history of treatment failure with multiple biologic agents, including TNF inhibitors, IL-17 inhibitors, and IL-12/23 inhibitors. Tofacitinib offers a different mechanism of action as a JAK inhibitor, potentially providing a novel therapeutic approach for their refractory psoriatic arthritis while considering their positive HLA-B27 status.	An alternative therapy concept for this patient, considering their extensive treatment history and lack of response to various biologic agents, could involve targeting a different pathway involved in the pathogenesis of psoriatic arthritis. Given their HLA-B27 positivity and the presence of enthesitis, a promising alternative option would be an interleukin-23 (IL-23) inhibitor.<br><br>Therefore, the alternative therapy concept I would recommend is:<br><br>Alternative Therapy Concept:<br><br>Drug: Guselkumab<br>Dosage: Guselkumab 100 mg subcutaneously at weeks 0, 4, and then every 8 weeks thereafter.<br>Guselkumab is a selective IL-23 inhibitor that has shown efficacy in the treatment of psoriatic arthritis, particularly in patients with enthesitis and moderate to severe skin involvement. By targeting the IL-23 pathway, guselkumab offers a different mechanism of action compared to TNF inhibitors and may provide therapeutic benefit in this refractory case. Regular monitoring for response and adverse effects is essential during treatment with guselkumab.	The recommendation of guselkumab as an alternative therapy for this patient is based on its mechanism of action as an IL-23 inhibitor, offering a different therapeutic approach from previous biologic agents. Given the patient's HLA-B27 positivity and the presence of enthesitis, targeting the IL-23 pathway may provide effective disease control in psoriatic arthritis while addressing their treatment refractoriness.

	goals. Regular follow-up visits should be scheduled to monitor treatment response and any adverse effects.			
**GPT-4**	Given the detailed case of a 58-year-old male with long-standing Psoriatic Arthritis (PsA), extensive previous therapy attempts with inadequate response or side effects, and current active disease despite upadacitinib treatment, the next step in therapy should be carefully considered. The European League Against Rheumatism (EULAR) recommendations for the management of PsA emphasize a tailored approach based on disease activity, specific manifestations (e.g., peripheral arthritis, enthesitis, dactylitis, axial disease, skin, and nail psoriasis), and comorbidities, as well as previous therapies' responses and tolerability.<br><br>Considering the patient's history of insufficient response to conventional synthetic DMARDs (csDMARDs), multiple biologic DMARDs (bDMARDs) including TNF inhibitors (adalimumab, infliximab, etanercept, certolizumab pegol), an IL-12/23 inhibitor (ustekinumab), IL-17 inhibitors (secukinumab, ixekizumab), and a T-cell co-stimulation modulator (abatacept), as well as an inadequate response to a targeted synthetic DMARD (tsDMARD) (upadacitinib, a JAK inhibitor), the choice of next therapy should target a different pathway or mechanism of action not yet explored in this patient.<br><br>Given the current EULAR guidelines and considering the patient's extensive drug history and the need to target a different pathway, a reasonable next step would be to consider a therapy that targets the IL-23 pathway exclusively, as this has not been directly targeted in this patient's previous therapies (ustekinumab targets both IL-12 and IL-23).<br><br>Guselkumab, an IL-23 inhibitor, could be a potential option. It is approved for the treatment of moderate to severe plaque psoriasis and has shown efficacy in Psoriatic Arthritis. Guselkumab targets a different aspect of the inflammatory pathway that has not been specifically targeted in this patient's therapy history. However, the decision to use guselkumab should be based on a thorough review of the patient's medical history, potential contraindications, and current EULAR guidelines.<br><br>It's essential to note that specific dosages and treatment regimens should be determined by a healthcare professional based on the patient's overall health status, potential for drug interactions, and other factors. This recommendation is made based on the information provided and with the understanding that treatment of PsA is complex and requires individualized consideration.	Guselkumab is suggested due to its mechanism of action targeting the IL-23 pathway, which is a key driver in the pathogenesis of psoriatic disease and has not been directly targeted by the patient's previous therapies. This choice is motivated by the need to explore a different inflammatory pathway, given the patient's history of inadequate response to therapies targeting TNF, IL-17, IL-12/23, and JAK inhibition, aiming to improve symptoms and control disease activity in a patient with refractory Psoriatic Arthritis.	Given the patient's extensive history of inadequate response to multiple therapies for Psoriatic Arthritis (PsA), an alternative therapy concept worth considering involves targeting a different inflammatory pathway that has not been directly targeted in this patient's treatment history.<br><br>Tofacitinib is an oral Janus kinase (JAK) inhibitor that modulates the immune system response. While the patient has been treated with upadacitinib, a specific JAK1 inhibitor, tofacitinib has a broader mechanism of action, inhibiting JAK1 and JAK3, which may offer a different therapeutic benefit. It is indicated for the treatment of PsA in adults who have had an inadequate response or who have been intolerant to one or more Disease-Modifying Anti-Rheumatic Drugs (DMARDs). Tofacitinib can be used in the management of both joint and skin symptoms of PsA.<br><br>The recommended dosage for PsA is tofacitinib 5 mg taken orally twice daily. This recommendation is based on its approval for PsA management and the need to explore alternative pathways and mechanisms of action in a patient with a complex treatment history and refractory disease. However, the decision to initiate tofacitinib should consider the patient's entire medical history, potential drug interactions, and current recommendations, emphasizing the importance of monitoring for side effects, given the safety profile of JAK inhibitors.	Tofacitinib is recommended as an alternative due to its broader inhibition of Janus kinases (JAK1 and JAK3), offering a different mechanism of action that may provide clinical benefits in Psoriatic Arthritis (PsA) not achieved with previous therapies, including upadacitinib, which specifically targets JAK1. This approach aims to address both joint and skin manifestations of PsA in a patient with a history of inadequate response to a wide range of targeted therapies, potentially offering a new avenue for disease control.

CASE VIGNETTE 12:

- Age: 40 years;
- Sex: female;
- Body height in cm: 161; Body weight in kg: 61; BMI: 23.5 kg/m^2;
- Diagnosis: Systemic lupus erythematosus (SLE) and secondary antiphospholipid syndrome (APS);
- Disease duration: 11 years (defined here as the time since the onset of symptoms, the diagnosis was made only 4 weeks ago)
- Previous illnesses: Hysterectomy for uterine myomatosis 5 years ago;
- Current medical history: Symptoms started around 12 years ago with a deep vein thrombosis on the right leg. She took one year of rivaroxaban. In the next 5 years she had recurrent thrombophlebitis of superficial leg veins (4 times in total) and a pelvic vein thrombosis in 2019. She took apixaban since then, did not have any thromboembolic events since then. In the last 6 months, she has experienced breathing-dependent pain and abdominal pain from time to time, but is currently symptom-free except for a butterfly-shaped erythema on the face;
- Current clinical examination: butterfly-shaped erythema of the cheeks, nose and forehead, involving the nasolabial folds. Other physical examination unremarkable, especially no signs of thrombosis in the examination. The further physical examination is unremarkable;
- Visual Analogue Scale (VAS) pain scoring: 0 (reference range 0-10);
- C-reactive protein (CRP): 0.4 mg/dl (reference range < 0.5 mg/dl);
- Erythrocyte sedimentation rate (ESR): 69 mm/h (reference range 3-8 mm/h);
- Immunoserology: Antinuclear antibody (ANA): 1:1000 AC-1; Anti-Ro/SSA: positive; double-stranded DNA antibodies slightly elevated: 32 IU/ml (<10 IU/ml), complement C3 and C4: normal; triple-positive antiphospholipid syndrome profile (LA, ACL IgG and anti- β2-GPI IgG antibodies);
- Urine analysis: Urine dipstick test and urine sediment analysis unremarkable;
- Other lab results: blood counts normal, kidney and liver function values normal;
- Tuberculosis elispot: negative;
- Hepatitis B serology: negative;
- Previous therapies for SLE: none;
- Systemic Lupus Erythematosus Disease Activity Index (SLEDAI): 4 (reference range 0-105);
- Medications taken regularly: apixaban 5 mg 1-0-1;
- Current abdominal sonography: No hepatosplenomegaly, no abdominal lymphadenopathy in the visible area of approx. 50 %. No free abdominal fluid. No pleural effusion;
- Current transthoracic echocardiography: Sinus rhythm. Heart cavities of normal size with normal wall thickness. Normal left ventricular systolic function (LVEF: 69 %). No evidence of regional left ventricular wall motion abnormalities. Normal diastolic function. Normal systolic pulmonary artery pressure (sPAP) with 29.9 mmHg + central venous pressure. Normal right ventricular function. Valves without significant vitium. No hepatic vein congestion. No pericardial effusion;
- Current pulmonary function test: physiological findings.

TREATMENT CONCEPTS FOR CASE VIGNETTE 12

	Therapy Concept	Reasoning behind the therapy concept	Alternative therapy concept	Reasoning behind the alternative therapy concept
**RB**	Hydroxychloroquine 200mg daily. Topical sun protection (sunmilk). Stop apixaban. Start phenprocoumon (target INR 2-3).	All patients with SLE should receive hydroxychloroquine to reduce longterm cardiovascular events. Current disease activity is not apparent (the described erythema of the face is not typical for acute cutaneous lupus, as nasolabial folds are affected. Rosacea could be a differential diagnosis). Although the last thrombosis is more than 3 years apart from detection of antiphospholipid-antibody detection, antiphospholipid syndrome is highly probable, why vitamin K-antagonists should be preferred over DOAKs	Hydroxychloroquine 200mg daily. Topical sun protection (sunmilk). Stop apixaban. Start warfarin (target INR 2-3).	There is no adequate alternative therapy to hydroxychloroquin. Low dose prednisolone would be less adequate. Vitamin K antagonists are interchangeable.
**GPT-3.5**	Based on the provided clinical information and current European guidelines (EULAR guidelines), a suitable therapy suggestion for this patient with Systemic Lupus Erythematosus (SLE) and secondary antiphospholipid syndrome (APS) would involve the initiation of immunosuppressive therapy to manage the SLE symptoms and prevent disease progression.<br><br>Therapy Concept:<br>Initiate treatment with hydroxychloroquine (HCQ) as a first-line agent for SLE management.<br><br>Specific Drug Name and Dosage:<br>Hydroxychloroquine (HCQ) 200 mg tablets orally once daily.<br><br>Rationale:<br><br>Hydroxychloroquine (HCQ) is recommended as a first-line agent in the treatment of SLE by EULAR guidelines.<br>HCQ has demonstrated efficacy in controlling disease activity, reducing flares, and improving overall outcomes in SLE patients.<br>It is also effective in preventing thrombotic events associated with APS.<br>HCQ has a favorable safety profile and is generally well-tolerated, making it suitable for long-term use.<br>Given the absence of severe organ involvement and the relatively low disease activity (SLEDAI score of 4), HCQ monotherapy is appropriate for initial management.<br>Additional Considerations:<br><br>Close monitoring of the patient's clinical response, disease activity, and laboratory parameters is essential to assess treatment effectiveness and adjust therapy as needed.<br>The patient should continue with anticoagulation therapy (apixaban 5 mg 1-0-1)	The choice of hydroxychloroquine (HCQ) as first-line therapy for this patient with SLE and secondary APS is supported by EULAR guidelines due to its demonstrated efficacy in controlling disease activity, its favorable safety profile, and its additional benefit in preventing thrombotic events associated with APS. Additionally, HCQ is suitable for long-term use, aligning with the chronic nature of SLE and APS management.	As an alternative therapy concept, considering the patient's relatively low disease activity and absence of severe organ involvement, a glucocorticoid-sparing immunosuppressive agent may be considered.<br>Therapy Concept:<br>Initiate treatment with mycophenolate mofetil (MMF) as an immunosuppressive agent for SLE management.<br>Specific Drug Name and Dosage:<br>Mycophenolate mofetil (MMF) 1 gram orally twice daily.<br>Rationale:<br>Mycophenolate mofetil (MMF) is recommended as an alternative immunosuppressive agent in SLE management, particularly in patients who require glucocorticoid sparing or have contraindications to hydroxychloroquine.<br>MMF has demonstrated efficacy in reducing disease activity, preventing flares, and improving long-term outcomes in SLE patients.<br>It is also effective in managing lupus nephritis, if present, which is a common manifestation in SLE.<br>MMF has a favorable safety profile, although monitoring for potential side effects such as gastrointestinal disturbances and myelosuppression is necessary.<br>Regular monitoring of disease activity, renal function, and	The alternative therapy suggestion of mycophenolate mofetil (MMF) is based on its efficacy as an immunosuppressive agent in reducing disease activity and preventing flares in SLE patients, particularly those requiring glucocorticoid sparing. MMF also offers the advantage of managing lupus nephritis if present, with a favorable safety profile, although regular monitoring for potential side effects is necessary.

	for secondary APS management.<br>Regular follow-up appointments with a rheumatologist or an interdisciplinary lupus clinic are recommended to optimize disease management and monitor for potential complications.<br>It's important to note that treatment decisions should always be made in consultation with a healthcare professional, considering individual patient factors and preferences.		complete blood count is essential during MMF therapy.<br>Additional Considerations:<br>The patient should continue with anticoagulation therapy (apixaban 5 mg 1-0-1) for secondary APS management.<br>Close monitoring of the patient's clinical response and laboratory parameters is crucial to assess treatment effectiveness and adjust therapy as needed.<br>Regular follow-up appointments with a rheumatologist or an interdisciplinary lupus clinic are recommended to optimize disease management and monitor for potential complications.<br>Again, treatment decisions should be made in consultation with a healthcare professional, considering individual patient factors and preferences.	

**GPT-4**	Given the detailed case of a 40-year-old female patient diagnosed with Systemic Lupus Erythematosus (SLE) and secondary Antiphospholipid Syndrome (APS), and considering the European League Against Rheumatism (EULAR) guidelines available up to my last update in April 2023, a specific therapy suggestion would focus on managing both the SLE and the thrombotic risks associated with APS.<br><br>For SLE with moderate disease activity (indicated by a SLEDAI score of 4) without major organ involvement but with positive autoantibodies (ANA, Anti-Ro/SSA, and double-stranded DNA antibodies slightly elevated), the EULAR guidelines recommend using hydroxychloroquine as a first-line therapy. Hydroxychloroquine is beneficial for its immunomodulatory effects, potential to reduce flares, and improve long-term outcomes in SLE patients.<br><br>Given her history of thrombotic events and the secondary APS diagnosis, continuing anticoagulation therapy is crucial. She is already on apixaban, which is appropriate for managing thrombosis risk in APS patients.<br><br>Specific Therapy Suggestion:<br><br>Hydroxychloroquine for SLE management, with a common starting dose being 5 mg/kg body weight per day (based on actual body weight). For a patient weighing 61 kg, this translates to approximately 300 mg per day. However, dosing should not exceed the recommended maximum to minimize the risk of retinopathy. The exact dose might be rounded to the nearest available tablet size (e.g., 200 mg or 400 mg tablets), and thus she might be prescribed either 200 mg daily or 400 mg daily, depending on her risk factors and physician's judgment.<br><br>Continue Apixaban 5 mg 1-0-1 for thrombosis prevention due to APS.<br><br>It's important to note that while hydroxychloroquine is generally well-tolerated, regular ophthalmologic monitoring is recommended due to the risk of retinopathy. Also, this patient should be monitored closely for any changes in symptoms or lab values, given the recent diagnosis and the potential for disease activity fluctuation.<br><br>This treatment plan is based on the information provided and the most current EULAR guidelines up to April 2023. Treatment of SLE and APS should be personalized, taking into account the patient's specific clinical scenario, comorbidities, and preferences. Regular follow-up with a rheumatologist and possibly a hematologist for APS management is advised to adjust therapy as needed and to monitor for side effects.	The choice of hydroxychloroquine is based on its efficacy in reducing flares and improving the long-term outcomes in SLE patients, as supported by EULAR guidelines, making it a cornerstone in the management of SLE due to its immunomodulatory effects. Continuing apixaban addresses the thrombotic risk associated with secondary Antiphospholipid Syndrome (APS), aligning with the recommendations for anticoagulation therapy in patients with APS to prevent further thrombotic events.	For an alternative therapy concept focusing on a patient with Systemic Lupus Erythematosus (SLE) and secondary Antiphospholipid Syndrome (APS), considering the patient's characteristics and the latest European guidelines up to my last update in April 2023, an alternative approach could involve the use of belimumab in addition to maintaining anticoagulation therapy. This recommendation considers that the patient has active SLE (as indicated by a moderate disease activity score and the presence of autoantibodies) and has not initiated SLE-specific immunosuppressive treatment yet.<br><br>Alternative Therapy Suggestion:<br><br>Belimumab, administered as an intravenous infusion, for the management of SLE. The approved dosage for adult patients is 10 mg/kg given at 2-week intervals for the first three doses and then every 4 weeks. This treatment is specifically recommended for patients with active, autoantibody-positive SLE who are receiving standard therapy, as it can help to reduce disease activity more effectively in some patients compared to standard therapy alone.<br><br>Continue Apixaban 5 mg 1-0-1 for the prevention of thrombosis due to APS.<br><br>Reason for the Alternative Therapy:<br>Belimumab has been approved for the treatment of SLE and is the first biological treatment that has been shown to be effective in a subset of SLE patients, particularly those with high disease activity and positive autoantibody status. It targets B-lymphocyte stimulator (BLyS), reducing the survival of B cells, including autoreactive B cells, and has been associated with reduced disease activity and flares. Maintaining anticoagulation with apixaban is essential for managing the patient's risk of thrombosis associated with APS, in line with current guidelines for APS management. This approach provides an immunomodulatory strategy targeting the underlying disease mechanisms of SLE while addressing the co-existing thrombotic risk.	Belimumab is selected for its targeted action against B-lymphocyte stimulator (BLyS), which is involved in the survival and differentiation of B cells, making it effective in reducing disease activity and preventing flares in patients with autoantibody-positive SLE. This choice is driven by its ability to specifically address the immunological aspects of SLE in patients who have shown active disease despite standard therapies, providing a targeted approach to disease management alongside necessary anticoagulation with apixaban for secondary APS, optimizing patient care by addressing both conditions concurrently.

- **CASE VIGNETTE 13:**
- Age: 32 years;
- Sex: female;
- Body height in cm: 150; Body weight in kg: 51; BMI: 22.7 kg/m^2;
- Diagnosis: Systemic lupus erythematosus (SLE);
- Disease duration: 5 months;
- Previous illnesses: Iron deficiency anemia, migraine;
- Current medical history: The patient reports night sweats, weight loss of 6 kg in the last 5 months and respiratory-dependent thoracic pain. There was diffuse myalgia all over the body, no joint-related pain. She suffers from pronounced fatigue and has recurrent severe unilateral headaches, albeit with known migraines;
- Current clinical examination: Patient in good general and nutritional condition. Inconspicuous integument. No palpably enlarged cervical lymph nodes. No cyanosis. No icterus. No signs of congestion. No peripheral edema. Heart sounds pure and rhythmic, no typical murmur. Auscultatory findings include a basally weakened breath sound on both sides on the right more than on the left, otherwise lungs equally ventilated, no murmurs. Abdomen soft, no tenderness, liver palpable directly under right costal arch. Neurological orientation unremarkable. Mild pressure pain over the upper arms and thighs without loss of muscle strength. Joint status: No synovitic joints. Good grip strength on both sides. Fist closure complete on both sides. No peripheral edema. Skin findings unremarkable. The neurological examination is unremarkable;
- Visual Analogue Scale (VAS) pain scoring: 7 (reference range 0-10);
- C-reactive protein (CRP): 2.4 mg/dl (reference range < 0.5 mg/dl);
- Erythrocyte sedimentation rate (ESR): 52 mm/h (reference range 3-8 mm/h);
- Immunoserology: Antinuclear antibody (ANA): >1:1000 AC-1; Ani-Smith (Sm): positive; anti-ribosomal P (Rib-P) phosphoproteins: positive; anti-Ro/SSA and anti-La/SSB: positive; double-stranded DNA antibodies: highly elevated above the detection limit (>379 IU/ml), complement C3, C4 decreased: C3: 25 mg/dl (reference range 75-140 mg/dl); C4: 3.9 mg/dl (reference range 10-34 mg/dl); Lupus anticoagulant test: normal, ACL and anti- β2-GPI antibodies: negative;
- Urine analysis: Urine dipstick test: ++++ for protein and ++ for erythrocytes, Differentiation of urine proteins: total protein/creatinine ratio: 4900 mg/g creatinine (reference range < 70 mg/g creatinine), albumin/creatinine ratio: 5090 mg/g creatinine (reference range < 30 mg/g creatinine), Urine sediment analysis: erythrocytes 70/µl, 29 % of akanthocytes;
- Results of the kidney biopsy: Histologically confirmed combined lupus nephritis class III with immunohistochemical full-house pattern, moderate signs of activity (modified National Institutes of Health NIH activity index 8/24) and few chronic changes (modified NIH chronicity index 2/12). In addition, there is mild membranous glomerulonephritis, but there is no evidence of an independent class V component;
- Other lab results: blood counts: normal, estimated glomerular filtration rate (eGFR): 63,5 ml/min due to cystatin c measurement, cholesterol: 225 mg/dl (130-220), triglycerides: 143 mg/dl (74-142 mg/dl), HDL-cholesterole: 64 mg/dl (>35 mg/dl); LDL cholesterol: 150 mg/dl (≤ 150 mg/dl);
- Tuberculosis elispot: negative;
- Hepatitis B serology: negative;
- Previous therapies for SLE: none;
- SLEDAI: 11 (reference range 0-105);

- Medications taken regularly: ibuprofen 800-2400 mg daily;
- Current abdominal sonography: Mild hepatosplenomegaly, no abdominal lymphadenopathy in the visible area of approximately 60 %. Small amount of free abdominal perihepatic fluid and in the pelvis cavity;
- Current transthoracic echocardiography: Sinus rhythm. Heart cavities of normal size with normal wall thickness. Normal left ventricular systolic function. No evidence of regional left ventricular wall motion abnormalities. Normal diastolic function. Normal systolic pulmonary artery pressure (sPAP) with 16 mmHg + central venous pressure. Normal right ventricular function. Valves without significant vitium. No hepatic vein congestion. Small, hemodynamically irreleveant pericardial effusion;
- Current chest X-ray: Minor pleural effusions on both sides, otherwise inconspicuous.

TREATMENT CONCEPTS FOR CASE VIGNETTE 13

	Therapy Concept	Reasoning behind the therapy concept	Alternative therapy concept	Reasoning behind the alternative therapy concept
**RB**	Start with mycophenolate mofetil 6g daily (500mg 2-2-2) and prednisolone 50mg daily (tapering to 5mg within 12 Weeks). Add 1000 IE colecalciferol and pantoprazole 40mg daily (if ibuprofen intake continues together with prednisolone). Contraception is necessary. Add belimumab 400mg weekly for 4 weeks, followed by 200mg weekly and hydroxychloroquine 200mg daily.	Active lupus nephritis should be treated with glucocorticoid pulse therapy, all patients with SLE should receive hydroxychloroquine to reduce longterm cardiovascular events. Contraception is necessary as long as MMF is taken. As large proteinuria is present also belimumab should be started.	Administer cyclophosphamide 500mg i.v. every 2 weeks, for a total of 6 times. Prednisolone 50mg daily (tapering to 5mg within 12 Weeks). 1000 IE Colecalciferol. Pantoprazole 40mg daily (if ibuprofen intake continues together with prednisolone). Contraception and ovary protection. Sulfamethoxazole/trimetoprime 960mg 3 times per week.	Induction therapy with cyclophosphamide is possible. Ovary protection is necessary if the patient plans a pregnancy in the future. Cotrim prohlylaxis against pneumocystis should be applied as long as cyclophosphamide is given or prednisolone dose is higher than 10mg daily.
**GPT-3.5**	Based on the provided clinical presentation and the most current European guidelines (EULAR guidelines), a suitable therapy suggestion for this patient with Systemic Lupus Erythematosus (SLE) and lupus nephritis would be to initiate induction therapy with a combination of glucocorticoids and immunosuppressive agents. Specifically, the patient should be started on:<br><br>Prednisone: Initiate with a dose of 1 mg/kg/day orally (or intravenously if necessary), not exceeding 60 mg/day. Once a response is achieved, the dose should be tapered gradually to the lowest effective maintenance dose, typically over several weeks to months.<br><br>Mycophenolate mofetil (MMF): Administer at a dose of 1000 mg orally twice daily. This dosage may be adjusted based on the patient's response and tolerance, with consideration given to increasing the dose to a maximum of 3 g/day if necessary.<br><br>Additionally, considering the patient's anemia and the presence of proteinuria, it's crucial to closely monitor renal function, blood pressure, and hematologic parameters throughout the course of treatment. Adequate supportive measures, including iron supplementation for the iron deficiency anemia and management of migraine symptoms, should also be incorporated into the patient's care plan.<br><br>It's important to note that treatment decisions should be made in consultation with a rheumatologist or specialist familiar with managing SLE, and the therapy regimen may need to be adjusted based on the patient's response to treatment and any emerging clinical considerations. Regular follow-up and monitoring are essential to assess treatment efficacy, manage potential side effects, and optimize long-term outcomes for the patient.	The combination of prednisone and mycophenolate mofetil (MMF) is a recommended induction therapy for lupus nephritis in the current EULAR guidelines due to its efficacy in achieving renal remission and preserving kidney function while minimizing corticosteroid-related adverse effects. Prednisone provides potent anti-inflammatory effects, while MMF, an immunosuppressive agent, targets the underlying autoimmune process, thus addressing both acute inflammation and long-term disease control in SLE.	An alternative therapy concept for this patient with Systemic Lupus Erythematosus (SLE) and lupus nephritis would be to initiate induction therapy with a combination of glucocorticoids and cyclophosphamide:<br><br>Prednisone: Initiate with a dose of 1 mg/kg/day orally (or intravenously if necessary), not exceeding 60 mg/day. Once a response is achieved, the dose should be tapered gradually to the lowest effective maintenance dose, typically over several weeks to months.<br><br>Cyclophosphamide: Administer intravenously at a dose of 500-1000 mg/m² body surface area once every 2 weeks for a total of 6 doses, followed by maintenance therapy with mycophenolate mofetil (MMF) or azathioprine for at least 3-6 months.<br><br>This alternative regimen may be considered in patients with severe lupus nephritis or contraindications to mycophenolate mofetil (MMF), with close monitoring for potential adverse effects such as bone marrow suppression and hemorrhagic cystitis. Treatment decisions should be made in consultation with a rheumatologist or specialist familiar with managing SLE, considering the patient's individual characteristics and preferences.	The alternative therapy suggestion of combining prednisone with cyclophosphamide is based on its effectiveness in inducing remission in severe cases of lupus nephritis, particularly when mycophenolate mofetil (MMF) is contraindicated or not tolerated. Cyclophosphamide, as a potent immunosuppressive agent, targets the underlying autoimmune process and has been shown to be beneficial in reducing renal inflammation and preserving kidney function in patients with SLE.

**GPT-4**	Given the detailed patient profile, the diagnosis of Systemic Lupus Erythematosus (SLE) with lupus nephritis (Class III with a full-house pattern and mild membranous glomerulonephritis), and considering the patient has not previously been treated for SLE, my therapy suggestion based on the most current European League Against Rheumatism (EULAR) guidelines would focus on addressing the active nephritis, which is a key concern in this case.<br><br>For a patient with active lupus nephritis, especially with a class III involvement and signs of significant proteinuria and renal impairment, the EULAR guidelines recommend the use of immunosuppressive therapy in addition to glucocorticoids to control the disease activity and protect kidney function.<br><br>A suitable single therapy concept for this patient, taking into account the need for specificity in drug choice and dosages, would be:<br><br>Mycophenolate Mofetil (MMF): Starting at a dosage of 1.5 to 2 g per day, divided into two doses. MMF is recommended because it has been shown to be effective in inducing remission in lupus nephritis and has a favorable side effect profile compared to cyclophosphamide, especially for women of childbearing age. It targets both the immunologic activity of lupus nephritis and helps in reducing the risk of further renal damage.<br>MMF is chosen over cyclophosphamide for initial treatment in this case due to its effectiveness in similar types of lupus nephritis and its relatively safer profile in terms of fertility preservation and lower risk of cytopenias. The dosage might be adjusted based on clinical response and tolerance.<br><br>The patient's response to treatment should be monitored through clinical evaluation and laboratory parameters, including repeat urine analysis, proteinuria levels, serum creatinine, complement levels, and anti-dsDNA antibodies, to assess the effectiveness of the therapy and adjust the treatment plan as necessary. Additionally, it's important to manage the patient's symptoms of SLE, monitor for potential side effects of the medication, and address any other complications or comorbid conditions that may arise during treatment.<br><br>Given the complexity of SLE and the individual variability in response to treatment, it's crucial to closely monitor the patient and adjust the treatment regimen as needed, in consultation with a rheumatologist experienced in managing SLE.	I recommended Mycophenolate Mofetil (MMF) for this patient primarily because it is effective in inducing remission of lupus nephritis, as evidenced by several studies and supported by EULAR guidelines. MMF is preferred for its favorable safety profile, particularly in terms of lower risks of infertility and fewer severe side effects compared to cyclophosphamide, making it a suitable choice for a young female patient of childbearing age.	As an alternative to Mycophenolate Mofetil, another therapy concept recommended by the European League Against Rheumatism (EULAR) guidelines for the treatment of lupus nephritis, particularly for a patient with the described profile, is:<br><br>Belimumab in addition to standard therapy: Belimumab, a biologic therapy approved for use in SLE, can be added to standard immunosuppressive treatment. For an adult patient, the dosage is typically 10 mg/kg given as an intravenous infusion on days 0, 14, and 28, and then every 4 weeks. Belimumab is recommended for patients with active autoantibody-positive SLE despite standard therapy, and it has been shown to be effective in reducing disease activity and preventing flares. This treatment option might be particularly beneficial in managing systemic manifestations of SLE in patients who have shown an inadequate response to conventional immunosuppressive agents or for whom these agents are contraindicated due to side effects or other concerns.<br>This recommendation is based on the efficacy of Belimumab in improving patient outcomes in SLE, including those with lupus nephritis, and its role in targeting B-lymphocyte stimulator (BLyS), a critical factor in the survival and differentiation of B cells, which play a significant role in the pathogenesis of SLE. The addition of Belimumab to standard therapy could offer a more targeted approach to modulating the immune response in SLE, providing a beneficial effect on disease activity and potentially improving renal outcomes.	Belimumab was suggested as an alternative therapy because it specifically targets B-lymphocyte stimulator (BLyS), which plays a crucial role in the survival and differentiation of B cells involved in the pathogenesis of SLE, offering a mechanism-based therapeutic option. It has been shown to reduce disease activity and prevent flares in SLE patients, making it an effective addition for those with active disease not adequately controlled by standard treatments, with a favorable impact on long-term outcomes and quality of life.

CASE 14:

- Age: 28 years;
- Sex: female;
- Body height in cm: 175; Body weight in kg: 80; BMI: 26.1 kg/m^2;
- Diagnosis: Systemic lupus erythematosus (SLE);
- Disease duration: 20 years;
- Previous illnesses: Diabetes mellitus type 1;
- Current medical history: The disease manifested at the age of 14 and was classified as juvenile idiopathic arthritis (type seronegative polyarthritis). The patient received methotrexate (MTX) during the first years of the disease. The disease was stable for several years and achieved remission, so that MTX could be discontinued at the age of 18 years. During a severe relapse with serositis (pleural effusions, pericardial effusion) and autoimmune hemolytic anemia at the age of 20 years, the diagnosis of SLE was made with typical autoimmune serology and therapy was escalated to MMF 3 g daily. Belimumab was added 6 months ago for pericardial effusion that had progressed under MMF. The patient currently still has pronounced arthralgias and feels very exhausted. An esophagogastroduodenoscopy and a colonoscopy were performed 8 months ago without any pathological findings;
- Current clinical examination: Patient in reduced general condition. Pale skin color without recognizable efflorescences. No palpable enlarged cervical lymph nodes. No peripheral edema, no signs of bleeding. Heart sounds pure and rhythmic, no typical murmur. Lungs equally ventilated, no murmurs. Abdomen soft, no tenderness, enlarged liver palpable one hand below the right costal arch, spleen also palpable. Neurological orientation unremarkable. Diffuse pressure pain over all hand and finger joints without clearly recognizable swelling. Gaenslen's sign positive. Forefoot pressure pain on both feet;
- Visual Analogue Scale (VAS) pain scoring: 7 (reference range 0-10);
- C-reactive protein (CRP): 0.4 mg/dl (reference range < 0.5 mg/dl);
- Erythrocyte sedimentation rate (ESR): 52 mm/h (reference range 3-8 mm/h);
- Immunoserology: Antinuclear antibody (ANA) >1:1000 AC-1; ENA screening test negative; double-stranded DNA antibodies elevated: 99 IU/ml (< 10 IU/ml), complement C3, C4 decreased: C3: 21 mg/dl (reference range 75-140 mg/dl), C4: 5 mg/dl (reference range 10-34 mg/dl); Lupus anticoagulant test: normal, ACL and anti- β2-GPI antibodies: negative; Rheumatoid factor: negative, anti-CCP antibodies: negative;
- Urine analysis: Urine dipstick test, urine protein analysis and urine sediment analysis inconspicuous;
- Other lab results: hemoglobin: 7.1 g/dl (reference range 12.0-14.6 g/dl); thrombocytes: $99*10^3$/µl (reference range $186\text{-}353*10^3$/µl), leucocytes: normal**,** total bilirubin: 2.1 mg/dl (reference range 0.1-1.2 mg/dl); haptoglobin: < 10 mg/dl (reference range 34-200 mg/dl); liver function values: normal, kidney function values: normal, electrolytes: normal, Folic acid deposit: balanced, vitamin B12 deposit: balanced, iron deposit: balanced;
- Tuberculosis elispot: negative;
- Hepatitis B serology: negative;
- SLEDAI: 9 (reference range 0-105);
- Medications taken regularly: MMF 500 mg 3-0-3: Belimumab 200 mg subcutaneously once a week, hydroxychloroquine 200 mg 1-0-0; prednisolone 5 mg 1-0-0; ferrosanol 100 mg 1-0-0

- Current abdominal sonography: Progressive hepatomegaly (liver size VAL 17 cm (previous examination 12.2 cm), STL 9.9 cm (VU 9.9 cm), splenomegaly of the same size as the previous findings (13x8x4.8 cm); otherwise inconspicuous;
- Current transthoracic echocardiography: Sinus rhythm. Heart cavities of normal size with normal wall thickness. Normal left ventricular systolic function. No evidence of regional left ventricular wall motion abnormalities. Normal diastolic function. Normal systolic pulmonary artery pressure (sPAP). Normal right ventricular function. Valves without significant vitium. No hepatic vein congestion. No pericardial effusion;
- Current chest X-ray: inconspicuous;
- Current pulmonary function test: physiological findings;
- Arthrosonography: not done.

TREATMENT CONCEPTS FOR CASE VIGNETTE 14:

	Therapy Concept	Reasoning behind the therapy concept	Alternative therapy concept	Reasoning behind the alternative therapy concept
**RB**	Initiate anifrolumab 300mg i.v. every 4 weeks. Increase prednisolone to 50mg daily (tapering to 5mg within 12 Weeks). Add 1000 IE Colecalciferol. Contraception is necessary. Continue MMF and hydroxycloroquine, stop belimumab.	A change in immunosuppressive therapy due to autoimmune hemolytic anemia despite MMF and belimumab should be made. Tuberculosis ELISPOT and hepatitis B serology should be done before.	Initiate rituximab 1g i.v. (day1, day15). Increase prednisolone 50mg daily (tapering to 5mg within 12 Weeks). Add 1000 IE colecalciferol. Contraception is necessary. Continuate MMF and hydroxycloroquine, stop belimumab.	Rituximab is off-label in SLE, but implemented in the EULAR recommendations and often efficatious in autoimmune hemolytic anemia. Hepatitis B serology has to be done before application of rituximab.
**GPT-3.5**	Based on the provided information and considering the current European guidelines (EULAR guidelines) for the treatment of systemic lupus erythematosus (SLE), the following therapy suggestion can be made:<br><br>Therapy Concept:<br>Introduce Rituximab as an additional therapy to address persistent arthralgias and exhaustion.<br><br>Drug Name and Dosage:<br>Rituximab 1000 mg intravenously, administered as two 500 mg infusions separated by two weeks, repeated every 6 months for a total of two doses.<br><br>Rationale:<br>Rituximab, a monoclonal antibody targeting CD20-positive B lymphocytes, has been shown to be effective in refractory cases of SLE, particularly in patients with persistent arthritis and exhaustion. By targeting B cells, Rituximab can help modulate the immune response and alleviate symptoms. This therapy concept aligns with the current treatment goals of achieving disease control and improving quality of life in SLE patients.<br><br>It's important to note that the addition of Rituximab should be carefully considered in consultation with a rheumatologist or immunologist, taking into account the patient's individual characteristics, disease severity, and potential risks and benefits of treatment. Close monitoring for adverse effects and therapeutic response is essential during Rituximab therapy.	The addition of Rituximab is justified due to the patient's persistent arthralgias and exhaustion despite current treatment with MMF, Belimumab, hydroxychloroquine, and low-dose prednisolone. Rituximab's mechanism of action in targeting CD20-positive B lymphocytes offers a targeted approach to further modulate the underlying immune dysregulation in systemic lupus erythematosus, potentially improving symptom control and quality of life.	An alternative therapy concept, considering the patient's current treatment and disease manifestations, would be to switch the patient to a calcineurin inhibitor-based regimen.<br><br>Therapy Concept:<br>Introduce Tacrolimus as an alternative immunosuppressant.<br><br>Drug Name and Dosage:<br>Tacrolimus 2 mg orally twice daily.<br><br>Rationale:<br>Tacrolimus, a calcineurin inhibitor, offers an alternative mechanism of immunosuppression that may be beneficial in refractory cases of SLE, particularly in patients with persistent arthralgias and exhaustion. Tacrolimus has been shown to effectively control disease activity in lupus nephritis and may also help alleviate extra-renal manifestations such as arthritis. This therapy concept aims to achieve better disease control and symptom relief while minimizing adverse effects associated with long-term corticosteroid use and other immunosuppressants. Close monitoring of drug levels and renal function is necessary during Tacrolimus therapy.	The alternative therapy suggestion of Tacrolimus is based on its potent immunosuppressive effects, particularly in refractory cases of SLE. Tacrolimus offers an alternative mechanism of action compared to the current medications and has demonstrated efficacy in controlling disease activity, including arthritis symptoms, thus providing a viable option to improve symptom management and overall disease control while minimizing corticosteroid dependence.

**GPT-4**	Given the detailed clinical picture of the patient with Systemic Lupus Erythematosus (SLE), taking into account the European League Against Rheumatism (EULAR) guidelines relevant to the management of SLE, and considering the patient's current therapy regimen and clinical status, a specific therapy suggestion is warranted. The patient has a history of diabetes mellitus type 1, indicating the need for careful selection of medications due to potential drug interactions and side effects related to glucose control.<br><br>Considering the current therapy with mycophenolate mofetil (MMF), belimumab, hydroxychloroquine, prednisolone, and ferrosanol, alongside the active symptoms of pronounced arthralgias, severe exhaustion, and the laboratory findings indicative of active disease (e.g., high ANA titers, elevated double-stranded DNA antibodies, low complement levels, and anemia), a modification in therapy may be beneficial.<br><br>The EULAR guidelines recommend individualized treatment plans based on disease activity, organ involvement, and the presence of comorbidities. Given the patient's active symptoms and laboratory findings suggesting moderate to severe disease activity, and considering the patient's history of diabetes mellitus which may limit the use of certain medications due to metabolic concerns, an addition of a biologic agent could be considered if not contraindicated and if the patient's condition is assessed to be inadequately controlled by the current regimen.<br><br>Specific Therapy Suggestion:<br><br>Continue the current treatment with MMF, hydroxychloroquine, and low-dose prednisolone, as these are standard treatments for SLE that address broad aspects of the disease and have steroid-sparing effects.<br>Given the ongoing active disease despite the current treatment, including the use of belimumab, which targets B-lymphocyte stimulator (BLyS), it might be beneficial to consider an alternative or additional biologic treatment that has a different mechanism of action, provided it aligns with the latest recommendations and the patient's specific condition.<br>Given the constraints of mentioning only one substance from each substance group and the need to select a therapy concept with specific drug names and dosages, without repeating a substance already mentioned in the patient's current regimen, it's challenging to recommend a new single agent without considering the full context of available and recommended treatments for SLE as per the latest EULAR guidelines. However, it's important to note that as of my last update, one possible approach (if aligned with the latest EULAR guidelines and considering the patient's specific clinical situation) could be the consideration of rituximab for patients with refractory disease.<br><br>Rituximab, an anti-CD20 monoclonal antibody, has been used in cases of refractory SLE, particularly for patients who do not respond adequately to conventional therapy, including	The suggestion to consider rituximab as an additional treatment option is based on its mechanism of action targeting CD20-positive B cells, which are implicated in the pathogenesis of SLE, particularly in cases that are refractory to standard treatments like MMF and belimumab. Rituximab has been used in clinical practice for patients with severe, active disease not adequately controlled by conventional therapies, offering a potential benefit in modifying the disease course by depleting B cells, which play a central role in autoantibody production and inflammation in SLE.	An alternative therapy concept for the patient, considering the limitations and partial response to current treatments, is the initiation of Voclosporin in the context of lupus nephritis management. While the patient's current symptoms do not explicitly mention lupus nephritis, Voclosporin has shown promise in broader SLE management due to its immunomodulatory effects and could be considered in cases where conventional treatments have been insufficient, especially when considering its approval for lupus nephritis which indicates its efficacy in severe manifestations of SLE.<br><br>Alternative Therapy Suggestion:<br><br>Voclosporin - This is a calcineurin inhibitor that has been recently approved for the treatment of adults with active lupus nephritis. Although the patient does not currently present with active kidney disease, Voclosporin's potent immunosuppressive properties might benefit her systemic disease activity, particularly if there's underlying renal involvement that's not overt. The usual dosage for lupus nephritis would be 23.7 mg orally twice daily, adjusted based on pharmacokinetics and patient response.<br>The rationale behind this alternative suggestion is Voclosporin's novel mechanism of action and its proven efficacy in a severe SLE manifestation, suggesting potential benefits in broader SLE management. This option might offer an innovative approach to managing refractory SLE, aiming to reduce disease activity and improve quality of life, although it's crucial to carefully consider the patient's overall health status and potential side effects.	The recommendation for Voclosporin as an alternative therapy is based on its recent approval for lupus nephritis and its potent immunosuppressive capabilities, which may offer a new avenue for managing systemic lupus erythematosus (SLE) symptoms in patients who have not adequately responded to traditional therapies. Its unique action as a calcineurin inhibitor, combined with a favorable safety and efficacy profile in lupus nephritis, suggests potential utility in broader SLE management, offering hope for improved disease control in refractory cases.

	MMF and belimumab. However, the specific dosage and administration schedule would need to be determined based on the patient's current health status, prior therapy responses, and potential contraindications:<br><br>Rituximab: The dosage often used in autoimmune diseases is 1000 mg administered intravenously on day 1 and day 15, followed by maintenance doses based on clinical evaluation.<br>Important Considerations:<br><br>Close monitoring of the patient's response to therapy, potential side effects, and adjustment of therapy as necessary.<br>Given the patient's diabetes mellitus, careful monitoring for infections or other complications is crucial.<br>Collaboration with a multidisciplinary team, including rheumatologists, endocrinologists, and possibly a hematologist, given the anemia and thrombocytopenia.<br>Note: The specific therapy suggestion should be discussed with the patient's healthcare provider, considering the most current EULAR guidelines, the latest clinical trials, and the patient's overall health status, preferences, and potential contraindications.			
**GPT-3.5**	Based on the provided information and considering the current European guidelines (EULAR guidelines) for the treatment of systemic lupus erythematosus (SLE), the following therapy suggestion can be made:<br><br>Therapy Concept:<br>Introduce Rituximab as an additional therapy to address persistent arthralgias and exhaustion.<br><br>Drug Name and Dosage:<br>Rituximab 1000 mg intravenously, administered as two 500 mg infusions separated by two weeks, repeated every 6 months for a total of two doses.<br><br>Rationale:<br>Rituximab, a monoclonal antibody targeting CD20-positive B lymphocytes, has been shown to be effective in refractory cases of SLE, particularly in patients with persistent arthritis and exhaustion. By targeting B cells, Rituximab can help modulate the immune response and alleviate symptoms. This therapy concept aligns with the current treatment goals of achieving disease control and improving quality of life in SLE patients.<br><br>It's important to note that the addition of Rituximab should be carefully considered in consultation with a rheumatologist or	The addition of Rituximab is justified due to the patient's persistent arthralgias and exhaustion despite current treatment with MMF, Belimumab, hydroxychloroquine, and low-dose prednisolone. Rituximab's mechanism of action in targeting CD20-positive B lymphocytes offers a targeted approach to further modulate the underlying immune dysregulation in systemic lupus erythematosus, potentially improving symptom control and quality of life.	An alternative therapy concept, considering the patient's current treatment and disease manifestations, would be to switch the patient to a calcineurin inhibitor-based regimen.<br><br>Therapy Concept:<br>Introduce Tacrolimus as an alternative immunosuppressant.<br><br>Drug Name and Dosage:<br>Tacrolimus 2 mg orally twice daily.<br><br>Rationale:<br>Tacrolimus, a calcineurin inhibitor, offers an alternative mechanism of immunosuppression that may be beneficial in refractory cases of SLE, particularly in patients with persistent arthralgias and exhaustion. Tacrolimus has been shown to effectively control disease activity in lupus nephritis and may also help alleviate extra-renal manifestations such as arthritis. This therapy concept aims to	The alternative therapy suggestion of Tacrolimus is based on its potent immunosuppressive effects, particularly in refractory cases of SLE. Tacrolimus offers an alternative mechanism of action compared to the current medications and has demonstrated efficacy in controlling disease activity, including arthritis symptoms, thus providing a viable option to improve symptom management and overall disease control while minimizing corticosteroid dependence.

	immunologist, taking into account the patient's individual characteristics, disease severity, and potential risks and benefits of treatment. Close monitoring for adverse effects and therapeutic response is essential during Rituximab therapy.		achieve better disease control and symptom relief while minimizing adverse effects associated with long-term corticosteroid use and other immunosuppressants. Close monitoring of drug levels and renal function is necessary during Tacrolimus therapy.	

CASE VIGNETTE 15:

- Age: 50 years;
- Sex: female;
- Body height in cm: 155; Body weight in kg: 55; BMI: 22.9 kg/m^2;
- Diagnosis: diffuse cutaneous systemic sclerosis (dcSSc)
- Disease duration: 1 year;
- Previous illnesses: none
- Current medical history: The patient reports ubiquitous pain in the entire skin and muscles and a feeling of tightness in the skin. The skin becomes increasingly firm. In particular, there was pain in the fingers. She could not say for sure whether there was also swelling of the joints. She can no longer open her mouth as wide. She can walk one floor without any problems, but on further floors she gets shortness of breath and a dry chesty cough. Heart palpitations or chest pain are denied.
- Current clinical examination: Edema sclerosis of the fingers and pronounces scleroderma, restricted mouth opening (3 cm distance between the rows of teeth), slight frenulum sclerosis, no pressure-pain or swollen joints but general ubiquitous pressure pain, full muscle strength 5 (0-5 scale) of all muscles tested according to Janda's manual muscle test, no forefoot pressure pain, grip strength good, abdomen soft, no pressure pain, bowel sounds normal, liver and spleen not palpable, cardiopulmonary examination findings unremarkable. No sensory disturbances. No cervical lymphadenopathy.
- mRSS (modified rodnan skin score): 28 (previous value 4 months ago: 14, reference range: 0-51)
- Visual Analogue Scale (VAS) pain scoring: 9 (reference range 0-10);
- C-reactive protein (CRP): 3.1 mg/dl (reference range < 0.5 mg/dl);
- Erythrocyte sedimentation rate (ESR): 47 mm/h (reference range 3-8 mm/h);
- Immunoserology: Antinuclear antibody (ANA): >1:1000 AC-29; positive autoantibodies against topoisomerase I (anti-Scl 70); further autoimmune serology is negative;
- Urine analysis: Urine dipstick test, urine protein analysis and urine sediment analysis: all inconspicuous;
- Other lab results: hemoglobin 11.1 g/dl (reference range 12.0-14.6 g/dl); thrombocytes and leucocytes normal**;** liver enzymes: normal; kidney function values: normal; electrolytes: normal; troponin elevated 375 pg/ml (reference range 0-14 pg/ml); NT-proBNP elevated: 942 pg/ml (reference range < 124 pg/ml);
- Tuberculosis elispot: negative;
- Hepatitis B serology: negative;
- Earlier therapy for SSc: methotrexate (MTX) 15 mg subcutaneously once per week, discontinued 3 months ago when switching to MMF, as the scleroderma was clearly progressive under MTX;
- Medications taken regularly: MMF 500 mg 2-0-2 (started 4 months ago)
- Current abdominal sonography: not done.
- Resting electrocardiography: Sinus rhythm with a heart rate of 70/min, indifference type, heart times normal, no regression of excitation disorders;
- Current transthoracic echocardiography: Sinus rhythm. Heart cavities of normal size with normal wall thickness. Normal left ventricular systolic function. No evidence of regional left ventricular wall motion abnormalities. Normal diastolic function. Borderline normal systolic pulmonary artery pressure (sPAP) with 26.82 mmHg + 5-10 mmHg central venous pressure. Normal right ventricular function. Valves without significant vitium. No hepatic vein congestion. Moist pericardium.

- Current cardiac MRI: Normal sized cardiac cavities with slender myocardium and good left ventricular systolic function without evidence of regionally accentuated wall motion abnormalities. No evidence of focal lesions in the T2-weighted image. After administration of contrast agent, evidence of diffuse intramyocardial fibrosis inferiorly basally and a flat aspect epicardially laterally midventricularly. Inconspicuous aspect of the pericardium. Moist pericardium.
- Current chest CT: Minor pleural reticulations and adjacent ground-glass thickening in the dorsal lower lobes on both sides. Minor lamellar pericardial effusion up to 6 mm seam width.
- Current pulmonary function test: No significant obstruction or restriction, slight diffusion disorder (DLCO 62 %);
- Arthrosonography: not done.

TREATMENT CONCEPTS FOR CASE VIGNETTE 15

	Therapy Concept	Reasoning behind the therapy concept	Alternative therapy concept	Reasoning behind the alternative therapy concept
**RB**	Administer cyclophosphamide 750mg/m2 every 3 weeks for at least 3 times (usually for 6 times). Prophylaxis for pneumocystis jirovecii pneumonia (sulfamethoxazole/trimetoprime 960mg 3 times per week).	The escalation of therapy is necessary, because skin disease is progressive there is probalbe heart involvement despite MMF.	Initiate rituximab 1g i.v. (day1, day15). Increase mycophenolate to 500mg 2-2-2.	Rituximab is off-label in systemic sclerosis. Regular monitoring of skin thickness and troponin are necessary, if both do not decrease an autologous hematopietic stem cell transplantation has to be performed.
**GPT-3.5**	Based on the provided information and considering the most current European guidelines (EULAR guidelines) for the treatment of diffuse cutaneous systemic sclerosis (dcSSc), I would suggest initiating the patient on a therapy regimen involving Tocilizumab.<br><br>Tocilizumab is a humanized monoclonal antibody against the interleukin-6 receptor (IL-6R), which has shown promising results in the treatment of dcSSc, particularly in patients who have failed or are intolerant to previous therapies. The recommended dosage for Tocilizumab in dcSSc, according to EULAR guidelines, is typically administered intravenously at a dose of 8 mg/kg every 4 weeks.<br><br>Given the patient's clinical presentation and disease severity, along with the evidence of disease progression despite previous therapy with methotrexate (MTX) and mycophenolate mofetil (MMF), Tocilizumab presents a viable option for disease management. Tocilizumab's mechanism of action targeting IL-6 can help mitigate inflammation and fibrosis associated with dcSSc, potentially leading to improvements in skin involvement, musculoskeletal symptoms, and pulmonary manifestations.<br><br>Therefore, I would recommend initiating the patient on Tocilizumab therapy at a dosage of 8 mg/kg administered intravenously every 4 weeks, in consultation with a rheumatologist experienced in the management of systemic sclerosis. Close monitoring of the patient's clinical response and potential adverse effects would be essential during the course of treatment.	The recommendation for Tocilizumab is based on its proven efficacy in targeting interleukin-6 (IL-6), a key cytokine implicated in the pathogenesis of systemic sclerosis. Given the patient's progressive disease despite previous treatment with methotrexate (MTX) and mycophenolate mofetil (MMF), Tocilizumab offers a targeted approach to mitigate inflammation and fibrosis, potentially leading to clinical improvement across multiple organ systems affected by diffuse cutaneous systemic sclerosis (dcSSc).	Rituximab, a monoclonal antibody targeting CD20-positive B cells, has demonstrated efficacy in the treatment of systemic sclerosis, particularly in patients with skin and lung involvement. The recommended dosage for Rituximab in systemic sclerosis is typically administered intravenously at a dose of 1,000 mg on days 1 and 15, with repeat courses as needed.<br><br>Given the patient's clinical presentation, including progressive skin involvement and pulmonary manifestations, Rituximab presents an alternative therapeutic option that could potentially modulate the autoimmune response and reduce disease activity in diffuse cutaneous systemic sclerosis (dcSSc). Close monitoring of treatment response and adverse effects would be warranted in collaboration with a rheumatologist experienced in managing systemic sclerosis.	The recommendation for Rituximab stems from its ability to target CD20-positive B cells, which play a significant role in the pathogenesis of systemic sclerosis. By depleting these B cells, Rituximab may help mitigate autoimmune processes, reduce inflammation, and potentially halt disease progression in diffuse cutaneous systemic sclerosis (dcSSc), particularly in cases refractory to conventional therapies like methotrexate and mycophenolate mofetil.

**GPT-4**	For a patient with diffuse cutaneous systemic sclerosis (dcSSc) characterized by rapid disease progression, significant skin involvement (as indicated by a high mRSS score), and organ involvement (evidenced by elevated cardiac markers and pulmonary involvement), the treatment strategy should aim at targeting the underlying disease process, reducing inflammation, and preventing further fibrosis and organ damage. Based on the most recent European League Against Rheumatism (EULAR) guidelines available up to my last update in April 2023, a suitable therapeutic approach for such a patient, considering the patient's history and current therapy, would be the use of tocilizumab.<br><br>Tocilizumab, an interleukin-6 (IL-6) receptor inhibitor, has shown promise in systemic sclerosis, particularly in patients with early diffuse disease and evidence of inflammation. It could be particularly relevant for this patient who has demonstrated disease progression despite treatment with methotrexate and mycophenolate mofetil (MMF), along with elevated CRP levels, indicating ongoing inflammation.<br><br>The specific therapy suggestion would be:<br><br>Tocilizumab administered as 162 mg subcutaneously once weekly.<br>This recommendation takes into account the patient's failure to respond adequately to MMF, the presence of significant skin and possible internal organ involvement, and the need for a therapy that can potentially address both the inflammatory and fibrotic components of systemic sclerosis. It is also based on the assumption that there are no contraindications to tocilizumab in this patient's case, such as active infections or a history of severe allergic reactions to biologic therapies.<br><br>It is crucial to monitor the patient closely for potential side effects associated with tocilizumab, including infections, changes in liver enzymes, neutropenia, and gastrointestinal perforations. Regular follow-up appointments should be scheduled to assess the effectiveness of the therapy and adjust it as necessary based on the patient's response and tolerance.<br><br>Please note that treatment recommendations can vary based on the most current research findings, guidelines updates, and the patient's overall health status. Therefore, it's essential to consult the most recent EULAR guidelines or other relevant guidelines and consider a multidisciplinary approach involving rheumatologists, dermatologists, and other specialists as appropriate for comprehensive care of patients with systemic sclerosis.	The suggestion of tocilizumab for this patient is based on its mechanism of action as an interleukin-6 (IL-6) receptor inhibitor, which targets inflammation and has been associated with improvements in skin fibrosis and function in systemic sclerosis. Given the patient's rapid disease progression despite previous therapies and elevated inflammatory markers (CRP), tocilizumab offers a targeted approach to modulate the immune response and potentially slow down the disease's progression.	For an alternative therapy concept in the management of diffuse cutaneous systemic sclerosis (dcSSc), particularly in a patient with significant skin involvement and evidence of interstitial lung disease (as suggested by pulmonary function test results and chest CT findings), nintedanib could be a viable option. Nintedanib is an antifibrotic agent that has been approved for the treatment of interstitial lung disease associated with systemic sclerosis (SSc-ILD).<br>The specific alternative therapy suggestion would be:<br>• Nintedanib at a dosage of 150 mg orally twice daily.<br>This recommendation is based on the ability of nintedanib to slow the rate of decline in lung function in patients with SSc-ILD, addressing a critical aspect of systemic sclerosis that significantly impacts morbidity and mortality. It's essential to monitor liver function tests regularly due to the risk of elevated liver enzymes and to manage gastrointestinal side effects that may occur.<br>Nintedanib's role in targeting the fibrotic component of SSc, especially in the lungs, provides a strategic treatment option for patients with pulmonary involvement, complementing the overall management strategy focused on controlling disease progression and improving quality of life.	Nintedanib was chosen as an alternative therapy for its antifibrotic properties, which have been demonstrated to slow the progression of interstitial lung disease in patients with systemic sclerosis, addressing a critical organ-specific manifestation of the disease. Its efficacy in SSc-ILD, coupled with a manageable safety profile, makes it a suitable option for patients with pulmonary involvement, aiming to preserve lung function and improve clinical outcomes in this complex, multi-system disease.

CASE VIGNETTE 16:

- Age: 61 years;
- Sex: female;
- Body height in cm: 162; Body weight in kg: 75; BMI: 28.6 kg/m^2;
- Diagnosis: Primary Sjogren syndrome;
- Disease duration: 5 years;
- Previous illnesses: Hashimoto's thyroiditis, arterial hypertension;
- Current medical history: The patient describes pronounced dry mouth and eyes, problems with tooth decay and complicated dental treatment as a result. There were arthralgias without joint swelling. What is new is a patchy skin rash on the lower extremities, which resembles bleeding and occurs intermittently. It first appeared spontaneously 6 months ago after a hiking vacation. This is now the 3rd episode of this rash. Due to the recent pronounced sicca symptoms, she has problems eating solid and dry food. Mild upper respiratory tract infections occur frequently (approximately 6 times a year), but she currently has no signs of infection.
- Current clinical examination: Skin rash with bluish purple spots (purpura) down the knee on both lower legs, one enlarged palpable lymph node in the right angle of the jaw (approximately 1.5 cm). Parotid gland slightly swollen on both sides (approximately 3 cm) and palpable with tenderness. No cyanosis. No jaundice. No signs of congestion. No peripheral edema. Heart sounds pure and rhythmic, no typical murmur. Lungs ventilated at the same level, no secondary murmurs. Abdomen soft, no tenderness, no palpable hepatosplenomegaly. Neurological orientation unremarkable.
  Joint status: tenderness in the wrists of both hands. No swollen joints. Good grip strength on both sides. Fist closure complete on both sides. No forefoot pressure pain.
- Visual Analogue Scale (VAS) pain scoring: 4 (reference range 0-10);
- C-reactive protein (CRP): 4.25 mg/dl (reference range < 0.5 mg/dl);
- Erythrocyte sedimentation rate (ESR): 79 mm/h (reference range 3-8 mm/h);
- Immunoserology: Antinuclear antibody (ANA): > 1:1000 AC-4; anti-Ro/SSA and anti-La/SSB positive; double-stranded DNA antibodies negative (reference range < 10 IU/ml), complement C3, C4: normal; Lupus anticoagulant test: normal, ACL and anti- β2-GPI antibodies: negative; highly positive rheumatoid factor 330 U/ml (reference range 0-14 U/ml); cryoglobulins positive 243 mg/l (reference range 0-80 mg/l)
- Urine analysis: Urine dipstick proteins + (negative for erythrocytes; leucocytes and nitrite); differentiation of urine proteins: total protein 150 mg/l (reference range < 120 mg/l); albumin/creatinine ratio: 26.9 mg/g creatinine (reference range < 30 mg/g creatinine);
- Other lab results: hypergammaglobulinemia IgG: 2510 mg/dl (690-1600 mg/dl), immunofixation: negative; blood counts: normal, kidney function values normal, liver function parameters: normal;
- Skin biopsy of the right lower leg: perivascularly accentuated, lymphocytic inflammatory infiltrate. The findings fit to leukocytoclastic vasculitis;
- Biopsy of the lower lip: Autoimmune adenitis grade IV according to Chisholm and Mason;
- Tuberculosis elispot: negative;
- Hepatitis B serology: negative;
- Previous therapies for sjogren's syndrome: hydroxychloroquine 200 mg 1-0-1 monday-saturday; 1-0-0 on sundays (weight-adapted)
- EULAR Sjogren's syndrome disease activity index (ESSDAI): 14 (high disease activity)
- Medications taken regularly: amlodipine, L-thyroxine;

- Current abdominal sonography: No hepatosplenomegaly, no abdominal lymphadenopathy in the visible area of approx. 50 %. No free abdominal fluid.
- Current transthoracic echocardiography: Sinus rhythm. Heart cavities of normal size with normal wall thickness. Normal left ventricular systolic function (LVEF: 69 %). No evidence of regional left ventricular wall motion abnormalities. Normal diastolic function. Normal systolic pulmonary artery pressure (sPAP) with 29.9 mmHg + central venous pressure. Normal right ventricular function. Valves without significant vitium. No hepatic vein congestion. No pericardial effusion.
- Current pulmonary function test: physiological findings;

TREATMENT CONCEPTS FOR CASE VIGNETTE 16:

	Therapy Concept	Reasoning behind the therapy concept	Alternative therapy concept	Reasoning behind the alternative therapy concept
RB	Saliva subsitutes (e.g. Glandosane Mundspray) and artificial tears (e.g. Artelac) both as needed and prednisolone 40mg daily (tapering to 5mg within 12 weeks). 1000 IE colecalciferol. Administer rituximab 1g i.v. (day 1, day 15)	Sicca symptomes should be treated symptomatical. EULAR recommendation for cyroglobulinemic vasculitis is rituximab and glucocorticoid.	Pilocarpin 5mg 1-1-1.<br>Prednisolone 40mg daily (tapering to 5mg within 12 Weeks). 1000 IE colecalciferol.<br>Azathioprine 150mg daily.	If saliva substitution and artificial tears are not sufficient pilocarpin can be applied against sicca. Against cryoglobulinemic vasculitis oral immunosupressants like azathioprine might work.
GPT-3.5	Based on the provided information and the European guidelines for the treatment of Sjogren's syndrome, considering the high disease activity (ESSDAI: 14) and the manifestation of leukocytoclastic vasculitis, the following therapy suggestion could be made:<br><br>Therapy Concept:<br><br>Rituximab (MabThera) infusion therapy.<br>Dosage:<br><br>Administer Rituximab intravenously at a dose of 1 gram (1000 mg) on days 1 and 15.<br>Rationale:<br><br>Rituximab, a monoclonal antibody targeting CD20 antigen on B cells, has shown efficacy in reducing disease activity and improving symptoms in patients with primary Sjogren's syndrome, particularly in those with systemic manifestations.<br>Rituximab has been recommended as a second-line treatment in patients with primary Sjogren's syndrome who have failed conventional therapy or have severe disease activity, as in the case of this patient with a high ESSDAI score.<br>Rituximab has also demonstrated effectiveness in treating leukocytoclastic vasculitis associated with autoimmune diseases like Sjogren's syndrome.<br>The dosing regimen of Rituximab as described above is commonly used in clinical practice and has been shown to be effective and well-tolerated in patients with autoimmune diseases, including Sjogren's syndrome.<br>Monitoring and Follow-up:<br><br>Regular monitoring of clinical symptoms, disease activity indices, and laboratory parameters to assess treatment response and adjust therapy as needed.<br>Follow-up visits with a rheumatologist to evaluate treatment efficacy, manage any adverse events, and optimize the patient's overall management plan.<br>It's important to note that treatment decisions should always be made in consultation with a rheumatologist or healthcare provider familiar with the patient's medical history and individual circumstances.	Rituximab was chosen due to its efficacy in reducing disease activity and improving symptoms in primary Sjogren's syndrome, particularly in cases of systemic manifestations and vasculitis, as seen in this patient. Its mechanism of action targeting CD20-positive B cells aligns with the pathophysiology of the disease, making it a suitable second-line therapy option according to European guidelines.	An alternative therapy concept for the patient could be:<br><br>Therapy Concept:<br><br>Mycophenolate mofetil (CellCept) oral therapy.<br>Dosage:<br><br>Initiate Mycophenolate mofetil at a dose of 1000 mg orally twice daily.<br>Rationale:<br><br>Mycophenolate mofetil, an immunosuppressive agent, has demonstrated efficacy in reducing disease activity and improving symptoms in primary Sjogren's syndrome, particularly in patients with systemic involvement.<br>This medication is recommended as a second-line treatment option in patients with Sjogren's syndrome who have failed conventional therapy or have severe disease activity, offering an alternative to Rituximab.<br>The prescribed dosage of 1000 mg twice daily is commonly used in clinical practice and has been shown to be effective and well-tolerated in patients with autoimmune diseases.<br>Monitoring and Follow-up:<br><br>Regular monitoring of clinical symptoms, disease activity indices, and laboratory parameters to assess treatment response and monitor for potential adverse effects.<br>Close follow-up visits with a rheumatologist to evaluate treatment efficacy, manage any adverse events, and adjust therapy as needed.<br>As with any treatment decision, individual patient factors,	Mycophenolate mofetil was selected as an alternative therapy due to its proven efficacy in reducing disease activity and improving symptoms in primary Sjogren's syndrome, particularly in cases where conventional therapy has failed or in patients with systemic involvement. Its immunosuppressive properties make it a suitable option for managing the autoimmune manifestations and vasculitis seen in this patient.

			preferences, and comorbidities should be considered, and discussions with a healthcare provider familiar with the patient's case are essential.	

**GPT-4**	Given the detailed clinical profile of the patient with primary Sjögren's syndrome, high disease activity as indicated by the EULAR Sjögren's syndrome disease activity index (ESSDAI) score of 14, and considering the current European League Against Rheumatism (EULAR) guidelines, a targeted approach is necessary to manage the complex manifestations of the disease, particularly in the context of the patient's pronounced sicca symptoms, leukocytoclastic vasculitis evidenced by skin biopsy, and high rheumatoid factor with cryoglobulins.<br><br>Considering the patient has already been treated with hydroxychloroquine, which is often a first-line agent for Sjögren's syndrome but appears insufficient in controlling her disease activity, stepping up the therapy is warranted. The presence of systemic features such as leukocytoclastic vasculitis and significant extraglandular manifestations (skin rash, persistent arthralgias) suggests the need for a more potent immunosuppressive or immunomodulatory agent.<br><br>Rituximab, a monoclonal antibody targeting CD20 on B cells, is recommended for patients with primary Sjögren's syndrome who have systemic involvement or refractory disease. Rituximab has shown efficacy in reducing glandular inflammation and systemic manifestations in several studies and is mentioned in the EULAR recommendations for the management of Sjögren's syndrome with systemic features.<br><br>A typical rituximab regimen for autoimmune conditions like Sjögren's syndrome involves:<br><br>Rituximab infusion: 1000 mg administered intravenously on day 0 and day 14, followed by a reassessment of disease activity and symptoms to decide on further treatment.<br>This recommendation is based on the assumption that there are no contraindications to rituximab in this patient (e.g., history of severe infections, severe cardiac disease). Pre-medication with steroids, acetaminophen, and antihistamines is often employed to reduce infusion reactions. Monitoring for infections and regular follow-up is crucial due to the immunosuppressive nature of the treatment.<br><br>It's important to note that treatment should be tailored to the patient's specific needs, considering potential side effects, comorbidities (like Hashimoto's thyroiditis and arterial hypertension), and the patient's overall health status. Coordination with the patient's healthcare team, including rheumatologists, ophthalmologists, and dentists, is essential for comprehensive care.	Rituximab is suggested due to its efficacy in treating systemic manifestations of primary Sjögren's syndrome, particularly in cases where conventional disease-modifying antirheumatic drugs (DMARDs) like hydroxychloroquine are insufficient. Its targeted action on B cells, which are implicated in the pathogenesis of Sjögren's syndrome, can help reduce glandular inflammation and manage systemic symptoms such as vasculitis and arthralgias, offering a potential improvement in the quality of life for patients with refractory or systemic disease.	For an alternative therapy concept, considering the patient's high disease activity, systemic manifestations, and previous inadequate response to hydroxychloroquine, Mycophenolate Mofetil (MMF) could be recommended. MMF inhibits inosine monophosphate dehydrogenase and exerts a cytostatic effect on lymphocytes, which are actively involved in the autoimmune response associated with Primary Sjogren Syndrome. It has been used in various systemic autoimmune diseases for its immunosuppressive properties.<br><br>The typical dosage for Mycophenolate Mofetil in the context of autoimmune diseases like pSS, based on its efficacy and tolerability, would be:<br><br>1000 mg orally twice daily.<br>This recommendation considers MMF's ability to control systemic symptoms and potentially modulate the underlying autoimmune processes without the B-cell depleting effects of Rituximab. MMF is often well-tolerated and can be effective in managing the systemic involvement and improving glandular function in pSS. However, monitoring for potential side effects, such as gastrointestinal disturbances and hematological abnormalities, is important. This alternative therapy offers a different mechanism of action that may be suitable for patients with certain contraindications to B-cell depleting therapies or those seeking a non-biologic immunosuppressive option.	Mycophenolate Mofetil (MMF) is recommended as an alternative due to its immunosuppressive action on lymphocytes, which are central to the pathogenesis of Primary Sjogren Syndrome, offering a means to control systemic symptoms and inflammation without the B-cell depletion associated with Rituximab. This option provides a balance between efficacy in reducing disease activity and a favorable safety profile, making it suitable for patients requiring systemic treatment but for whom B-cell targeting therapy may not be appropriate or preferred.

CASE VIGNETTE 17:

- Age: 49 years;
- Sex: male;
- Body height in cm: 181; Body weight in kg: 77; BMI: 23.5 kg/m^2;
- Diagnosis: Eosinophilic granulomatosis with polyangiitis (EGPA);
- Disease duration: unclear;
- Previous illnesses: arterial hypertension, bronchial asthma;
- Current medical history: The patient is currently disoriented and unable to make meaningful contact. Oriented to person, but not to time, place and situation. Diffuse pain in the legs is reported. Otherwise, there are no other complaints. The patient lives alone. The neighbor had informed the emergency services when he found the neighbor unoriented in the stairwell. Unfortunately, no further anamnestic information is available;
- Current clinical examination: Reduced general status and normal nutritional status. No cyanosis. No dyspnea at rest. Skin: inconspicuous, Enorally dry mucous membranes. Discrete pretibial edema. Lungs: Basally on the right attenuated breath sound. Otherwise, vesicular breath sounds over all lung fields. Abdomen: slight ubiquitous defensive tension, pressure pain in the left lower abdomen, bowel sounds present in all quadrants, spleen not palpable. Heart: regular cardiac action, no murmurs. Abdomen: inconspicuous. Pulse status: inconspicuous. No flow noises over the carotid arteries and the aorta. Neurology: cranial nerves intact, pupillary reaction consensual, pupils isocor, sock-like hypesthesia of both feet and in the area of the ulnar nerve on the left, pronounced allodynia and painful dysesthesia, dysesthesias emphasized in both feet. Distally emphasized leg paresis on the left with leading left foot drop;
- Visual Analogue Scale (VAS) pain scoring: 7 (reference range 0-10);
- C-reactive protein (CRP): 5.2 mg/dl (reference range < 0.5 mg/dl);
- Erythrocyte sedimentation rate (ESR): 71 mm/h (3-8 mm/h);
- Immunoserology: Antinuclear antibody (ANA): negative, c-ANCA (anti-neutrophil cytoplasmic antibody): negative, p-ANCA: negative;
- Other lab results: absolute Leukocytosis: 18200/µl (reference range 3900/µl-8700/µl); 47 % of eosinophils in the differential hemogram; Creatinine: 1.24 mg/dl (reference range 0-1.17 mg/dl), estimated glomerular filtration rate (eGFR): 73 ml/min (reference range > 60 ml/min), Creatine kinase: 840 U/l (reference range 0-190 U/l); liver enzymes normal;
- Urine analysis: Urine dipstick test: erythrocytes +; protein ++, protein differentiation: total protein 321 mg/l (reference range < 120 mg/l), total protein/creatinine ratio 540 mg/g creatinine (reference range < 70 mg/l); albumin/creatinine 349 mg/g creatinine (reference range < 30 mg/g creatinine); urine sediment analysis: detection of dysmorphic erythrocytes, no acanthocytes;
- Tuberculosis elispot: negative;
- Hepatitis B serology: negative, protective anti-HBs titer;
- Previous therapies for eGPA: none;
- Medications taken regularly: salbutamol spray as required, enalapril, amlodipine, metoprolol, clonidine;
- Current abdominal sonography: No hepatosplenomegaly, no abdominal lymphadenopathy in the visible area of approximately 50 %. No free abdominal fluid. No pleural effusion.
- Current transthoracic echocardiography: Sinus rhythm. Heart cavities of normal size with normal wall thickness. Normal left ventricular systolic function (LVEF: 69 %). No evidence of regional left ventricular wall motion abnormalities. Normal diastolic function. Normal systolic

pulmonary artery pressure (sPAP). Normal right ventricular function. Valves without significant vitium. No hepatic vein congestion. No pericardial effusion;

- Current pulmonary function test: Mild obstructive ventilation disorder; No restrictive ventilation disorder, no diffusion disorder;
- Cranial MRI and MR angiography**:** Bihemispheric, partly acute and subacute diffusion disturbances as evidence of ischemia, without evidence of hemorrhage. No occlusions and stenoses of the arteries supplying the brain;
- CT chest: Multiple small nodular to circumscribed flat consolidations distributed in the lungs, consistent with lung involvement in the context of small-vessel vasculitis.

TREATMENT CONCEPTS FOR CASE VIGNETTE 17:

	Therapy Concept	Reasoning behind the therapy concept	Alternative therapy concept	Reasoning behind the alternative therapy concept
**RB**	Cyclophosphamide 750mg/m2 every 3 weeks, usually for 6 times. Prophylaxis for pneumocystis jirovecii pneumonia (sulfamethoxazole/trimetoprime 960mg 3x per week). Prednisolone 250mg i.v. for 3 days, followed by 80mg daily (tapering until 5mg daily within 12 weeks). Add colecalciferol 1000IE daily (as long as prednisolone is taken). Start azathioprine 150mg daily 4 weeks (best with an increasing dose i.e. 75mg daily for 2 weeks, then 150mg daily) after last cyclophosphamide.	Organ/life-threatening cerebral EGPA requires induction therapy with CYC and high-dose glucocorticoids. After remission induction (about 4 weeks after the last CYC-cycle) azathioprine should be started.	Rituximab 375mg/m2 weekly for 4 times. Sulfamethoxazole/trimetoprime 960mg 3x per week. Prednisolone pulse with 250mg i.v. for 3 days, followed by 80mg daily (tapering until 5mg daily within 12 weeks). Colecalciferol 1000IE daily (as long as prednisolone is taken). 4 months after rituximab and if remission is achieved, rituximab maintainence follows 500mg i.v. d1 and day15, then 500mg every 6 months.	Induction therapy for life-threatening EGPA can be achieved by rituximab. If remission is achieved by rituximab, maintenance therapy should also be rituximab.
**GPT-3.5**	Based on the provided patient information and considering the most current European guidelines, a suitable therapy concept for eosinophilic granulomatosis with polyangiitis (EGPA) would involve initiating glucocorticoid therapy along with an adjunctive immunosuppressive agent.<br><br>Therapy Suggestion:<br><br>Glucocorticoid: Prednisone<br><br>Initial dose: 1 mg/kg/day orally (77 mg/day for a 77 kg patient)<br>Tapering schedule: Gradual tapering over weeks to months based on clinical response and normalization of inflammatory markers.<br>Adjunctive Immunosuppressive Agent: Rituximab<br><br>Dosage: 375 mg/m² body surface area once weekly for four weeks<br>Total dose: 1,000 mg per cycle<br>Administration: Intravenous infusion<br>Rationale:<br><br>Glucocorticoids are the cornerstone of initial therapy for EGPA to rapidly suppress inflammation and control disease activity. Prednisone is commonly used at an initial dose of 1 mg/kg/day, which can then be tapered gradually based on clinical response and normalization of inflammatory markers.<br><br>Rituximab, a monoclonal antibody targeting CD20-positive B cells, has shown efficacy in inducing and maintaining remission in EGPA, particularly in patients with refractory disease or those at risk of glucocorticoid toxicity. It is recommended by EULAR guidelines as a first-line option for remission induction in severe cases of EGPA.<br><br>This combination therapy aims to achieve rapid control of disease activity with glucocorticoids while minimizing the cumulative dose and duration of glucocorticoid exposure through the	The combination of prednisone and rituximab is recommended due to its efficacy in rapidly suppressing inflammation, inducing remission, and reducing the risk of glucocorticoid-related adverse effects, particularly in severe cases of EGPA where conventional therapy alone may not be sufficient according to EULAR guidelines. Prednisone addresses acute inflammation, while rituximab targets B-cell involvement, offering a comprehensive approach to disease management.	An alternative therapy concept for EGPA could involve the use of a combination of glucocorticoids and cyclophosphamide, particularly in cases where rituximab is not readily available or contraindicated.<br><br>Therapy Suggestion:<br><br>Glucocorticoid: Prednisone<br><br>Initial dose: 1 mg/kg/day orally (77 mg/day for a 77 kg patient)<br>Tapering schedule: Gradual tapering over weeks to months based on clinical response and normalization of inflammatory markers.<br>Immunosuppressive Agent: Cyclophosphamide<br><br>Dosage: 2 mg/kg/day orally or 0.5-1 g/m² intravenously once monthly<br>Duration: Typically administered for 3-6 months, followed by transition to a maintenance immunosuppressive regimen if needed.<br>Rationale:<br><br>Glucocorticoids are essential for rapidly suppressing inflammation and controlling disease activity in EGPA, while cyclophosphamide serves as an immunosuppressive agent targeting both B and T lymphocytes, helping to induce remission.<br><br>Cyclophosphamide has demonstrated efficacy in inducing remission in severe	The alternative therapy suggestion of combining glucocorticoids with cyclophosphamide is based on its established efficacy in inducing remission, particularly in severe cases of EGPA with rapidly progressive or severe organ involvement. Cyclophosphamide serves as an alternative immunosuppressive agent when rituximab is not available or contraindicated, providing an additional option for achieving disease control according to EULAR guidelines.

	addition of rituximab, thereby reducing the risk of glucocorticoid-related adverse effects.<br><br>Close monitoring of the patient's clinical status, inflammatory markers, and adverse effects of therapy is essential throughout treatment. Adjustments to the treatment plan may be necessary based on the individual patient's response and tolerability.		cases of EGPA and is considered an alternative to rituximab according to EULAR guidelines, particularly in patients with rapidly progressive or severe organ involvement.<br><br>Close monitoring of the patient's clinical status, inflammatory markers, and adverse effects of therapy is crucial, given the potential for significant side effects associated with cyclophosphamide, such as bone marrow suppression, infection, and bladder toxicity.<br><br>The choice between rituximab and cyclophosphamide may depend on factors such as disease severity, comorbidities, patient preferences, and drug availability, necessitating individualized treatment decisions in collaboration with the patient and healthcare team.	

**GPT-4**	Given the patient's diagnosis of Eosinophilic Granulomatosis with Polyangiitis (EGPA), also known as Churg-Strauss syndrome, and considering the latest European League Against Rheumatism (EULAR) guidelines for the management of vasculitis, a specific therapy suggestion would involve the use of glucocorticoids combined with an additional immunosuppressive agent, given the severity and organ involvement indicated in the patient's presentation. However, as per the request to mention only one substance from the same substance group, focusing on a singular treatment approach is key.<br><br>For initial induction therapy in severe EGPA cases, especially with evidence of organ-threatening or life-threatening disease (as suggested by the CNS involvement, elevated eosinophil count, and potential renal involvement indicated by the urine analysis and slightly elevated creatinine), cyclophosphamide or rituximab are commonly recommended options. Given the instruction to provide a specific therapy concept with drug names and dosages and to only mention one substance name from the same substance group, I will focus on:<br><br>Rituximab: It is an anti-CD20 monoclonal antibody that depletes B-cells, which are implicated in the pathogenesis of EGPA. The use of Rituximab for EGPA is supported for cases where there is a contraindication to cyclophosphamide, when previous therapies have failed, or when there is relapsing disease.<br><br>The typical dosage for Rituximab in the context of EGPA, according to the EULAR guidelines, would be:<br><br>1,000 mg administered intravenously on day 1 and day 15, followed by maintenance doses depending on the clinical response and relapse. Maintenance therapy might involve repeated cycles every 6 months for 2 years, but this depends on individual patient response and tolerability.<br>It's important to note that Rituximab therapy should be accompanied by glucocorticoids to control inflammation rapidly. The glucocorticoid therapy would typically start with a high dose (e.g., prednisolone 1 mg/kg/day, max 60 mg/day) and then be tapered based on the patient's response and EULAR guidelines.<br><br>This treatment recommendation is made with the assumption that the patient's condition is severe and potentially life-threatening given the CNS involvement and multi-organ issues. The choice between cyclophosphamide and rituximab would be made based on a thorough consideration of the patient's specific case, including previous medication history, risk of infertility with cyclophosphamide, and potential for hepatitis B reactivation with rituximab.<br><br>As always, this treatment should be initiated and monitored by a specialist in rheumatology or clinical immunology, considering the patient's comprehensive medical history, current medications, and potential for drug interactions and side effects. Regular follow-up is essential to	I suggested Rituximab for this patient with Eosinophilic Granulomatosis with Polyangiitis (EGPA) because it is effective in treating cases where the disease is refractory to standard therapies or when there are contraindications to cyclophosphamide, such as fertility concerns or previous adverse reactions. Furthermore, Rituximab targets B-cells, which play a crucial role in the pathogenesis of EGPA, potentially leading to a reduction in disease activity and prevention of relapses in patients with severe or life-threatening manifestations, as evidenced by the patient's CNS involvement and renal impairment.	As an alternative therapy concept for this patient with Eosinophilic Granulomatosis with Polyangiitis (EGPA) and severe multi-organ involvement, Mepolizumab represents a targeted treatment option. Mepolizumab is an interleukin-5 (IL-5) antagonist monoclonal antibody that specifically targets eosinophilic inflammation, a hallmark of EGPA.<br><br>The recommended dosage for Mepolizumab in the treatment of EGPA, based on clinical trials and the most recent guidelines up to April 2023, is 300 mg administered subcutaneously once every 4 weeks. This therapy is particularly beneficial for patients with a history of asthma and significant eosinophilia, as it directly targets the eosinophilic component of the disease, which is responsible for much of the pathology in EGPA.<br><br>Mepolizumab has been shown to reduce the need for systemic corticosteroids while maintaining disease control and reducing the rate of relapses. This makes it a valuable option for patients who are either steroid-refractory or for whom steroid side effects are a concern, addressing both the eosinophilic and the vasculitic aspects of the disease with a favorable safety profile.	Mepolizumab is recommended as an alternative due to its targeted action against IL-5, a key driver of eosinophil production and survival, thus directly addressing the eosinophilic component of EGPA and reducing eosinophil-mediated inflammation and damage. Its use is supported by evidence showing efficacy in reducing relapse rates, decreasing corticosteroid dependency, and improving symptoms and quality of life in patients with EGPA, making it an excellent option for those with severe eosinophilic manifestations and a history of asthma.

	monitor the effectiveness of the therapy and adjust as necessary.				

CASE VIGNETTE 18:

- Age: 62 years;
- Sex: male;
- Body height in cm: 172; Body weight in kg: 75; BMI: 25.4 kg/m^2;
- Diagnosis: ANCA (anti-neutrophil cytoplasmic antibody)-associated vasculitis;Disease duration: 3 years;
- Previous illnesses: Condition after hepatitis B virus infection, spinal canal stenosis L4/L5
- Previous medical history: The patient describes a chronic cold for almost 10 years. Three months ago he developed hearing loss in his left ear. He also has nosebleeds from time to time. He does not suffer from coughing or shortness of breath. Foaming urine is also denied. No skin florescences. With known spinal canal stenosis, he suffers from chronic back pain, but has hardly any joint pain and never any joints swelling.
- Current clinical examination: Slightly reduced general status. Nose shape inconspicuous. Incrustations and old blood deposits in the nasal vestibule. Hearing loss in the left ear and tapping pain over the left mastoid. No cyanosis. No dyspnea at rest. Skin: inconspicuous, discrete pretibial edema. Lungs: Vesicular breath sounds on both sides. Abdomen: Auscultation and palpation unremarkable. Heart: regular cardiac action, no murmurs. Pulse status: inconspicuous. Orienting neurological examination unremarkable. Pressure pain in the lumbar spine. No other joints that are painful and swollen.
- Visual Analogue Scale (VAS) pain scoring: 2 (reference range 0-10);
- C-reactive protein (CRP): 5.94 mg/dl (reference range < 0.5 mg/dl);
- Erythrocyte sedimentation rate (ESR): 73 mm/h (reference range 3-8 mm/h);
- Other lab results: Blood counts normal. Creatinine: 1.21 mg/dl (reference range 0-1.17 mg/dl), estimated glomerular filtration rate (eGFR): 59 ml/min (reference range > 60 ml/min), liver function values normal, lactate dehydrogenase 310 U/l (reference range < 250 U/l), IgG normal;
- Immunoserology: Antinuclear antibody (ANA): negative, c-ANCA (anti-neutrophil cytoplasmic antibody): positive 1:100; p-ANCA: positive, 1:100; proteinase-3 (Pr-3) antibody: positive, 39 IU/ml (reference range ≤ 2 IU/ml); myeloperoxidase (MPO) antibody: positive, 7 IU/ml (reference range ≤ 3.5 IU/ml); complement C3/C4: normal; rheumatoid factor (RF): negative; anti-cyclic citrullinated peptide (CCP) titer: negative; double-stranded DNA antibodies: negative;
- Urine analysis: Urine dipstick test: protein +, differentiation of urine proteins: total protein 190 mg/l, albumin/creatinine ratio 36 mg/g (reference range < 30 mg/g creatinine); a1-microglobulin 16 mg/l (reference range < 12 mg/l), gamma and kappa light chains negative;
- Tuberculosis elispot: negative;
- Hepatitis B serology: negative, protective anti-HBs titer;
- Previous therapies for small vessel vasculitis: none;
- Medications taken regularly: ibuprofen 400 mg daily;
- Computed tomography of the paranasal sinuses: Moderate mucosal swelling especially in the left ethmoid cells and in the right sphenoid sinus more than on the left and basally in both maxillary sinuses on the right more than on the left. The bony margins are intact. Subtotally occluded mastoid cells on the left;
- Chest CT scan: No indication of lung involvement as part of the underlying disease. No pleural effusions;

- Biopsy of the mucous membrane of the nasal sinus: chronic, lymphocyte-rich fibrosing rhinitis or granulomatous, eosinophil-rich, giant cell containing and fibrosing rhinitis with polyangiitis.

TREATMENT CONCEPTS FOR CASE VIGNETTE 18:

	Therapy Concept	Reasoning behind the therapy concept	Alternative therapy concept	Reasoning behind the alternative therapy concept
**RB**	Methotrexate 20mg s.c. once weekly. Folic acid 5mg p.o. once weekly 24h after methotrexate. Prednisolone 20mg daily (tapering and stopping within 12 weeks is recommended). Add colecalciferol 1000IE daily (as long as prednisolone is taken) and pantoprazole 40mg daily, if ibuprofen intake continues together with prednisolone.	Not organ-threatening GPA can be treated with methotrexate and it is the condition after hepatitis B. Therefore rituximab is the 2nd choise. Double positivitiy for ANCAs (PR3+ and MPO+) is uncommon and should prompt search for drug abuse (e.g. cocaine).	Administer rituximab 375mg/m2 weekly for 4 times. Add prophylaxis for pneumocystis jirovecii pneumonia with sulfamethoxazole/trimetoprime 960mg 3 times per week. Prednisolone 20mg daily (tapering and stopping within 12 weeks is recommended). Add colecalciferol 1000IE daily (as long as prednisolone is taken). Add pantoprazole 40mg daily, if ibuprofen intake continues together with prednisolone. 6 months after rituximab and if remission is achieved, rituximab maintainence follows 500mg i.v. d1 and day15, then 500mg every 6 months.	Condition after hepatitis B virus infection is described, but status of HBs-antigen and anti-HBc titer is not noted. Assuming positivity for anti-HBc-antibodies and negativity for HBs-antigen entecavir 0,5mg daily should be coadministered to avoid hepatitis B reactivation due to B cell depletion.
**GPT-3.5**	Based on the provided clinical information and considering the European guidelines for the management of ANCA-associated vasculitis (AAV), particularly granulomatosis with polyangiitis (GPA), here's a therapeutic suggestion for the patient:<br><br>Therapy Concept:<br><br>Induction Phase: Rituximab (375 mg/m^2 body surface area) once weekly for 4 weeks in combination with glucocorticoids.<br>Rationale:<br><br>Rituximab, a monoclonal antibody targeting CD20 antigen on B cells, is recommended as first-line induction therapy for AAV by the European League Against Rheumatism (EULAR) guidelines.<br>It has shown efficacy comparable to cyclophosphamide in inducing remission while having a favorable safety profile.<br>Rituximab has been increasingly favored due to its ability to deplete B cells, which play a crucial role in the pathogenesis of AAV.<br>The dosage of 375 mg/m^2 body surface area once weekly for 4 weeks is commonly used for induction therapy in AAV and is supported by evidence from clinical trials.<br>Combining rituximab with glucocorticoids is standard practice for induction therapy in AAV to achieve remission promptly and to prevent relapse.<br>Additional Considerations:<br><br>Glucocorticoid dosing should be tailored based on disease severity and individual patient factors, aiming for a rapid reduction once remission is achieved to minimize adverse effects.<br>Close monitoring for infectious complications, especially during the induction phase, is essential given the immunosuppressive nature of rituximab and glucocorticoids.	The therapy suggestion of rituximab in combination with glucocorticoids for induction phase in ANCA-associated vasculitis is supported by EULAR guidelines due to rituximab's efficacy in B cell depletion, which is central to the pathogenesis of the disease, along with glucocorticoids for their anti-inflammatory effects to achieve remission promptly and prevent relapse. This combination has demonstrated comparable efficacy to cyclophosphamide-based regimens with potentially fewer adverse effects.	An alternative therapy concept, considering the patient's clinical profile, would be:<br><br>Alternative Therapy Concept:<br><br>Induction Phase: Cyclophosphamide (oral or intravenous) at a dosage of 2 mg/kg/day in combination with glucocorticoids.<br>Rationale:<br><br>Cyclophosphamide, a cytotoxic agent, is a well-established induction therapy for ANCA-associated vasculitis, particularly granulomatosis with polyangiitis (GPA), according to EULAR guidelines.<br>The dosage of 2 mg/kg/day, whether administered orally or intravenously, is commonly used for induction therapy and has demonstrated efficacy in achieving remission.<br>Combining cyclophosphamide with glucocorticoids is a standard practice to induce remission rapidly and prevent disease progression.<br>Additional Considerations:<br><br>Close monitoring for potential adverse effects of cyclophosphamide, such as bone marrow suppression, hemorrhagic cystitis, and increased risk of infections, is crucial.<br>Once remission is achieved, consideration should be given to	The alternative therapy suggestion of cyclophosphamide in combination with glucocorticoids is based on its well-established efficacy as an induction therapy for ANCA-associated vasculitis, supported by EULAR guidelines. This regimen has demonstrated effectiveness in achieving remission and preventing disease progression, particularly in patients with contraindications to or preference against rituximab therapy.

	After achieving remission, the patient should be transitioned to maintenance therapy, which may include rituximab at a reduced frequency or other immunosuppressive agents, as per guidelines. This therapeutic approach aims to induce remission effectively while minimizing the risk of adverse effects, providing the patient with the best opportunity for disease control and long-term outcomes. However, treatment decisions should always be individualized based on the patient's clinical status, comorbidities, and preferences, and discussed thoroughly with the healthcare team.		transitioning to maintenance therapy, typically with a less toxic agent such as azathioprine or methotrexate, to sustain remission and minimize long-term adverse effects. Individualized dosing adjustments and monitoring of renal function are necessary, particularly given the patient's history of spinal canal stenosis and potential impact on drug metabolism and excretion. While rituximab is a preferred alternative due to its favorable safety profile, cyclophosphamide remains a viable option, especially in cases where rituximab is contraindicated or not readily available. Treatment decisions should be made collaboratively with the patient, taking into account their preferences, comorbidities, and potential risks and benefits of each therapy option.	

**GPT-4**	Given the patient's diagnosis of ANCA-associated vasculitis (AAV) with specific clinical manifestations including hearing loss, nosebleeds, and chronic rhinosinusitis without lung involvement, and considering the background of a previous hepatitis B virus infection, the most current European guidelines (EULAR guidelines) would suggest a tailored approach for induction of remission that considers both efficacy and safety profile due to the patient's comorbidities.<br><br>For a patient like this, Rituximab is a recommended therapy for induction of remission in AAV, including both granulomatosis with polyangiitis (GPA) and microscopic polyangiitis (MPA), especially in patients with relapsing disease, or when cyclophosphamide is contraindicated or in the presence of significant comorbidities such as previous hepatitis B infection. Rituximab is preferred due to its effectiveness in inducing remission and its relatively favorable safety profile compared to cyclophosphamide, particularly regarding infection risk and gonadal toxicity.<br><br>The specific recommended dose for Rituximab in the context of AAV induction therapy is:<br><br>375 mg/m² body surface area (BSA) once weekly for 4 weeks or<br>A fixed dose of 1000 mg administered intravenously on days 1 and 15.<br>Given the patient's body surface area (which can be calculated based on height and weight), the appropriate dosing regimen should be determined. However, the fixed-dose regimen is commonly used and may be preferable for its simplicity and effectiveness.<br><br>Additionally, considering the patient's hepatitis B history, antiviral prophylaxis should be considered to prevent reactivation, as Rituximab is associated with an increased risk of hepatitis B reactivation in carriers or those with resolved infection. Close monitoring of liver enzymes and hepatitis B viral load is also recommended during and after treatment with Rituximab.<br><br>This recommendation is in line with the most recent EULAR guidelines for the management of ANCA-associated vasculitis, which emphasize the use of Rituximab for induction of remission, especially in cases with certain comorbidities or contraindications to cyclophosphamide. However, it's crucial to tailor the treatment plan to the individual patient, considering all aspects of their health status and previous medical history.	I recommended Rituximab for several reasons: Firstly, it is effective in inducing remission in ANCA-associated vasculitis, including in patients with renal involvement and those with relapsing disease, making it suitable for the patient's condition. Secondly, Rituximab's safety profile is preferable for patients with a history of hepatitis B, as it poses a lower risk of reactivation compared to cyclophosphamide, especially when antiviral prophylaxis is employed, aligning with the need to manage the patient's complex medical history carefully.	As an alternative therapy concept for a patient with ANCA-associated vasculitis (AAV) without severe organ-threatening disease, but requiring treatment initiation, methotrexate can be considered for induction of remission in cases where rituximab is not suitable or available. Methotrexate is recommended for patients with non-severe AAV as it is effective in controlling the disease and has a well-established safety profile in the management of autoimmune diseases.<br><br>The typical starting dosage of methotrexate for induction of remission in AAV is 15-20 mg once weekly, administered orally or subcutaneously, with a gradual increase in dose based on clinical response and tolerance, up to a maximum of 25 mg/week. Folic acid supplementation, usually 5 mg once per week on a day different from the methotrexate administration, is also recommended to reduce the risk of side effects such as gastrointestinal disturbances and hepatotoxicity.<br><br>This recommendation takes into account the patient's overall health status, including their previous hepatitis B infection (where careful monitoring for liver function is essential) and their ability to tolerate methotrexate. It provides an effective and less costly alternative to biologics for inducing remission in non-severe cases of AAV, with the advantage of oral administration. However, patient monitoring for potential adverse effects, especially hepatotoxicity and bone marrow suppression, is crucial.	Methotrexate is chosen as an alternative due to its efficacy in controlling non-severe forms of ANCA-associated vasculitis and its utility as a less intensive, immunosuppressive option compared to biologics, making it suitable for patients with less aggressive disease. Its established use in autoimmune conditions, alongside manageable side effects and the convenience of oral administration, makes it a viable option for long-term disease management, especially when balancing the need for efficacy with safety considerations such as the patient's past hepatitis B infection.

CASE VIGNETTE 19:

- Age: 65 years;
- Sex: female;
- Body height in cm: 151; Body weight in kg: 60; BMI: 26.3 kg/m^2;
- Diagnosis: ANCA (anti-neutrophil cytoplasmic antibody)-associated vasculitis;
- Disease duration: 2 years;
- Previous illnesses: none;
- Current medical history: Initially, 2 years ago, the patient presented with chronic sinusitis and borky rhinitis. Treatment with azathioprine was initiated. A few weeks earlier, the patient was coughing up blood and the patient's respiratory condition deteriorated rapidly. The patient had to be intubated in an emergency and is currently being ventilated in the intensive care unit. There is a significantly elevated c-ANCA titer with a significantly elevated PR-3-ANCA of 280 U/l (reference range ≤ 2 U/ml).
- Current clinical examination: Lungs ventilated on both sides, ubiquitous coarse bubbling rales on both sides. Abdomen: inconspicious; Heart: regular cardiac action, no murmurs. Pulse status: inconspicuous. Neurology: unremarkable;
- C-reactive protein (CRP): 11.3 mg/dl (reference range < 0.5 mg/dl);
- Erythrocyte sedimentation rate (ESR): 92 mm/h (reference range 3-8 mm/h);
- Laboratory results: absolute Leukocytosis 10100/µl (reference range 3900/µl-8700/µl), differential hemogram: relative neutrophilia 89% (reference range 42.5-73.2), relative lymphopenia 9.8% (reference range 42.5-73.2%), absolute lymphocytes 0.72*1000/µl (reference range 1.16-3.18). Creatinine 1.18 mg/dl (reference range 0-1.17 mg/dl), other lab values including liver function parameters, creatinine kinase and troponine: normal
- Immunoserology: Antinuclear antibody (ANA): negative, c-ANCA positive, PR3-ANCA 280 U/ml (reference range ≤ 2 U/ml)
- Urine analysis: Urine dipstick test and urine sediment analysis inconspicuous;
- Tuberculosis elispot: negative;
- Hepatitis B serology: negative, protective anti-HBs titer;
- Current therapy for ANCA-associated vasculitis: azathioprine 200 mg daily, prednisolone 5 mg daily;
- Previous therapies for ANCA-associated vasculitis: none;
- Other medications taken regularly: currently analgosedation and catecholamines;
- Current CT chest: Bipulmonary predominantly centrally and peribronchovascularly arranged patchy consolidations with adjacent milky glass-like infiltrates, here it is not possible to distinguish between inflammatory atypical infiltrates and hemorrhages by image morphology. No intrapulmonary granulomas. No pericardial or pleural effusion.

TREATMENT CONCEPTS FOR CASE VIGNETTE 19:

	Therapy Concept	Reasoning behind the therapy concept	Alternative therapy concept	Reasoning behind the alternative therapy concept
**RB**	Administer rituximab 375mg/m2 i.v. weekly for 4 times and start prophylaxis for pneumocystis jirovecii pneumonia with sulfamethoxazole/trimetoprime 960mg 3x per week. Prednisolone 250mg i.v. for 3 days, followed by 80mg p.o. daily (tapering until 5mg daily within 12 weeks). Colecalciferol 1000IE daily (as long as prednisolone is taken). 4 months after rituximab and if remission is achieved, rituximab maintainence follows 500mg i.v. day1 and day15, then 500mg every 6 months.	If an infectious cause of the lung infiltrates has been excluded via bronchoalveolar lavage the most probable diagnosis is GPA with alveolar hemorrhage.	Administer cyclophosphamide 750mg/m2 every 3 weeks, usually for 6 times and start sulfamethoxazole/trimetoprime 960mg 3x per week. Prednisolone 250mg i.v. for 3 days, followed by 80mg p.o. daily (tapering until 5mg daily within 12 weeks). Colecalciferol 1000IE daily (as long as prednisolone is taken). Start azathioprine 150mg daily 4 weeks after last cyclophosphamide.	Maintenance therapy could also be done with rituximab (instead of azathioprine).
**GPT-3.5**	Based on the provided information and current European guidelines (EULAR guidelines), for the treatment of ANCA-associated vasculitis with severe pulmonary involvement, the following therapy suggestion can be made:<br><br>Therapy Concept:<br><br>Initiate induction therapy with a combination of glucocorticoids and cyclophosphamide.<br>Specific Drug Names and Dosages:<br><br>Cyclophosphamide: Intravenous pulse therapy with cyclophosphamide at a dose of 15 mg/kg every 2 to 3 weeks, with adjustments based on renal function. Alternatively, oral cyclophosphamide at a dose of 2 mg/kg daily can be considered.<br>Glucocorticoids: Prednisolone at a dose of 1 mg/kg/day (maximum 80 mg/day) orally or intravenously for induction therapy, followed by a tapering regimen aiming to reach a maintenance dose of 5-10 mg/day once remission is achieved.<br>Explanation:<br><br>Cyclophosphamide is a potent immunosuppressive agent used for induction therapy in severe ANCA-associated vasculitis with organ-threatening manifestations, such as pulmonary involvement leading to respiratory failure.<br>Glucocorticoids are administered concomitantly with cyclophosphamide to achieve rapid control of inflammation. They are used at high doses initially and tapered gradually to minimize adverse effects.<br>Additional Considerations:<br><br>Close monitoring of renal function, blood counts, and infectious complications is essential during therapy.<br>Once remission is achieved, transition to maintenance therapy with azathioprine or methotrexate should be considered to minimize long-term toxicity associated with cyclophosphamide.<br>Prophylaxis against Pneumocystis jirovecii pneumonia with trimethoprim-sulfamethoxazole should be initiated during immunosuppressive therapy.<br>It's important to note that the choice of therapy and dosages should be individualized based on	The combination of glucocorticoids and cyclophosphamide is recommended for induction therapy in severe ANCA-associated vasculitis with pulmonary involvement leading to respiratory failure, as per EULAR guidelines. This approach aims to rapidly suppress inflammation and prevent further organ damage, particularly in cases where the patient's condition has deteriorated rapidly and requires intensive care management.	An alternative therapy concept for ANCA-associated vasculitis with severe pulmonary involvement, considering the patient's condition and current treatments, could involve the following:<br><br>Therapy Concept:<br><br>Initiate induction therapy with rituximab.<br>Specific Drug Names and Dosages:<br><br>Rituximab: Administer rituximab at a dose of 375 mg/m² body surface area weekly for four weeks or 1000 mg IV on days 1 and 15, as per the RAVE trial protocol.<br>Explanation:<br><br>Rituximab, a monoclonal antibody targeting CD20-positive B cells, has shown efficacy comparable to cyclophosphamide in inducing remission in ANCA-associated vasculitis with less toxicity, particularly in patients with severe pulmonary involvement. This approach may be considered as an alternative to cyclophosphamide-based therapy, especially in patients with contraindications to cyclophosphamide or those who prefer a less toxic regimen.<br>It's crucial to discuss the risks and benefits of rituximab therapy with the patient and consider factors such as comorbidities, treatment preferences, and previous treatment responses before making a decision. Close monitoring for infusion reactions and infectious complications is necessary during rituximab therapy.	Rituximab offers a less toxic alternative to cyclophosphamide-based therapy for induction treatment of ANCA-associated vasculitis with severe pulmonary involvement, as it effectively depletes B cells implicated in the pathogenesis of the disease, with comparable efficacy and potentially lower risks of adverse effects such as infertility and malignancy. This approach may be particularly suitable for patients with contraindications to cyclophosphamide or those seeking alternative treatment options.

	the patient's clinical status, comorbidities, and preferences, and should be carried out in consultation with a rheumatologist or nephrologist experienced in managing vasculitis.			
**GPT-4**	For a patient with ANCA-associated vasculitis (AAV), especially with a severe manifestation as described, the European League Against Rheumatism (EULAR) guidelines recommend induction of remission with high-dose glucocorticoids and either rituximab or cyclophosphamide, followed by maintenance therapy. Given the patient's condition, including rapid deterioration and intensive care unit (ICU) ventilation, a more aggressive induction therapy is warranted.<br><br>Considering the patient's previous use of azathioprine for maintenance (which is inadequate for managing severe flares or induction of remission in critical cases), and the need for a single therapy concept from each substance group, I would recommend:<br><br>Rituximab as the induction therapy agent. Rituximab is a monoclonal antibody that targets CD20-positive B lymphocytes and has been shown to be effective for induction of remission in AAV. The standard dose for induction of remission in AAV is 1,000 mg intravenously (IV) on day 1 and day 15, alongside glucocorticoids. This regimen is based on the RAVE and RITUXVAS trials, which demonstrated rituximab's efficacy in AAV comparable to cyclophosphamide, with a potentially more favorable safety profile in certain patient groups.<br>Given the patient's critical condition and the presence of significant organ involvement (pulmonary hemorrhage), rituximab could offer a more favorable balance between efficacy and safety. The choice of rituximab over cyclophosphamide in this context also considers the long-term risk profile, especially regarding infection risk, malignancy, and fertility preservation, which might be more relevant in non-critical aspects but still essential for holistic patient care.<br><br>It's crucial to closely monitor the patient's response to therapy and adjust the treatment plan based on clinical improvement, side effects, and laboratory markers of disease activity. Maintenance therapy should be considered following successful induction of remission, with options including azathioprine or methotrexate, depending on the patient's condition and response to the induction therapy.<br><br>Please note, the specific therapy should be tailored to the patient's individual condition, potential drug interactions, and side effects profile, under the guidance of a multidisciplinary team experienced in managing ANCA-associated vasculitis.	I recommended Rituximab for several key reasons: Firstly, Rituximab is effective for induction of remission in severe ANCA-associated vasculitis, as evidenced by its success in major clinical trials (RAVE and RITUXVAS), showing comparable efficacy to cyclophosphamide but with a potentially more favorable safety profile. Secondly, given the patient's severe disease manifestation and previous inadequate response to azathioprine, Rituximab provides a targeted approach to deplete B cells, which are crucial in the pathogenesis of ANCA-associated vasculitis, offering a chance for rapid and sustained remission.	As an alternative to Rituximab, I would recommend Cyclophosphamide for the induction of remission in severe ANCA-associated vasculitis. Cyclophosphamide has a long history of use in severe cases of AAV and is particularly effective in cases with life-threatening organ involvement, such as pulmonary hemorrhage. The therapy concept would be as follows:<br><br>Cyclophosphamide administered intravenously (IV) as part of the standard induction regimen. The European Vasculitis Study (EUVAS) group protocols suggest a dosage of 15 mg/kg IV every 2 to 3 weeks for 3 to 6 months, adjusted based on age, renal function, and white blood cell count, with a usual maximum dose of 1.2 grams per administration. This regimen is often accompanied by high-dose glucocorticoids to control inflammation rapidly. Cyclophosphamide is chosen for its potent immunosuppressive effects, which can rapidly control the autoimmune response in ANCA-associated vasculitis. Its use is supported by extensive clinical experience and evidence demonstrating its efficacy in inducing remission in severe cases. However, careful monitoring for adverse effects, particularly infections, bladder toxicity, and bone marrow suppression, is essential due to its potent immunosuppressive and cytotoxic profile.	Cyclophosphamide is selected as an alternative therapy due to its proven efficacy in rapidly inducing remission in severe forms of ANCA-associated vasculitis, especially in cases with critical organ involvement. Its long-standing use in clinical practice is supported by robust evidence, making it a reliable choice for managing life-threatening manifestations of the disease, despite its potential for significant side effects.

CASE VIGNETTE 20:

- Age: 81 years;
- Sex: male;
- Body height in cm: 168; Body weight in kg: 78; BMI: 27.6 kg/m^2;
- Diagnosis: giant cell arteritis (GCA);
- Disease duration: 7 months;
- Previous illnesses: Aortic valve stenosis, diabetes mellitus type 2; arterial hypertension; dyslipidemia;
- Current medical history: The patient reports night sweats, subfebrile temperatures up to 37.9 °C and a weight loss of 7 kg in the last 4 months. Significantly elevated inflammatory parameters (CRP 8.0 mg/dl, reference range < 0.5 mg/dl) were detected by the family doctor and he was referred to a hospital. Broadly effective antibiotic therapies, including piperacillin/tazobactam, had no therapeutic benefit. A FDG-PET/CT examination revealed aortitis of the thoracic aorta. The patient never had any visual disturbances. For about 7 months, there had been slight left-temporal headaches, especially when pressure was applied to the corresponding area, and the patient reported morning stiffness lasting 1.5 hours and a feeling of heaviness in the shoulder girdle, both of which, however, had already clearly regressed under prednisolone;
- Current clinical examination: Reduced general condition, cachectic nutritional status, both lungs unremarkable on percussion and auscultation, systolic murmur above the aortic valves, abdomen unremarkable on auscultation and palpation, pain on movement of the shoulder joints on both sides, limited elevation up to approx. 80°, no swollen and painful joints. Fist closure completely possible on both sides. Temporal region on the left slightly sensitive to pressure. Temporal artery not prominent. Pulses of the temporal arteries and of the extremities arteries strongly palpable on both sides. No flow noise over the aorta;
- C-reactive protein (CRP): 7.8 mg/dl (reference range < 0.5 mg/dl);
- Erythrocyte sedimentation rate (ESR): 81 mm/h (reference range 3-8 mm/h);
- Other laboratory results: absolute leukocytosis 15.7*1000/µl and thrombocytosis 510*1000/µl, kidney and liver function values normal;
- Immunoserology: Antinuclear antibody (ANA): negative; ENA (extractable nuclear antigen) screening: negative; IgG4: normal; complement C3/C4: normal; rheumatoid factor (RF): negative; anti-cyclic citrullinated peptide (CCP) titer: negative; double-stranded DNA antibodies: negative;
- Urine analysis: Urine dipstick test: glucose ++++, otherwise unremarkable;
- Tuberculosis elispot: negative;
- Hepatitis B serology: negative;
- Medications taken regularly: metformin, dapagliflozin, candesartan, rosuvastatin;
- Arthrosonography of both shoulder joints: Right: No capsular expansion, rounded humeral head with clear minus defects, rotator cuff with calcifications, long biceps tendon intact, hypoechoic peritendinous rim and grade II hyperperfusion, AC joint arthrosis, no bursitis. Assessment: Most likely degenerative glenohumeral changes and florid biceps tendinitis, Left: No capsular expansion, rounded humeral head with minus defects, rotator cuff and long biceps tendon intact, hypoechoic peritendinous rim and grade II hyperperfusion, acromioclavicular joint arthrosis, no bursitis, no calcifications. Assessment: Most likely degenerative glenohumeral changes and florid biceps tendinitis;
- Current tempory artery ultrasound: Halo sign of the superficial temporal artery on the left and the occipital artery on the left;

- Current FDG PET/CT scan: Findings compatible with a less florid large vessel vasculitis of the intrathoracic aorta, especially aortic arch.

TREATMENT CONCEPTS FOR CASE VIGNETTE 20:

Case vignette number	Therapy Concept	Reasoning behind the therapy concept	Alternative therapy concept	Reasoning behind the alternative therapy concept
**RB**	Start with tocilizumab 162mg s.c. once per week and prednisolone 40mg daily (tapering over 20 weeks until 5mg daily). Colecalciferol 1000IE daily (as long as prednisolone is taken).	Giant cell arteritis without ischemic symptoms (no i.v. glucocorticoids necessary) but high risk for glucocorticoid-induce adverse events (therefore start of a DMARD). FDG PET/CT scan has to be evaluated for pulmonary TBC signs, as this is not described.	Methotrexate 20mg s.c. once weekly and folic acid 5mg p.o. once weekly 24h after methotrexate. Prednisolone 40mg daily (tapering over 20 weeks until 5mg daily) and colecalciferol 1000IE daily (as long as prednisolone is taken).	According to EULAR recommendations methotrexate is an alternative DMARD to tocilizumab.
**GPT-3.5**	Based on the provided patient information and considering the most current European guidelines, a suitable therapy suggestion for the patient with giant cell arteritis (GCA) would be:<br><br>Therapy Concept:<br>Initiate glucocorticoid therapy with prednisolone.<br><br>Specific Drug Name and Dosage:<br>Prednisolone: Start with an initial dose of 40-60 mg/day orally.<br><br>Rationale:<br><br>Prednisolone is the first-line treatment for giant cell arteritis (GCA) according to EULAR guidelines.<br>The patient's symptoms, including morning stiffness and shoulder girdle pain, have shown improvement with prednisolone, indicating responsiveness to glucocorticoid therapy.<br>The presence of significantly elevated inflammatory parameters supports the need for immediate and aggressive treatment.<br>A starting dose of 40-60 mg/day is appropriate for the management of GCA, with subsequent tapering based on clinical response and normalization of inflammatory markers.<br>It's important to closely monitor the patient for any adverse effects associated with prednisolone therapy, particularly considering his age and comorbidities such as diabetes mellitus and arterial hypertension. Additionally, adjunctive therapy with low-dose aspirin may be considered to reduce the risk of ischemic events. Regular follow-up assessments are necessary to evaluate treatment response and adjust the dosage accordingly.	The therapy suggestion of initiating prednisolone is based on its status as the first-line treatment for giant cell arteritis (GCA) according to EULAR guidelines. Prednisolone effectively suppresses inflammation, mitigating symptoms and reducing the risk of complications associated with GCA.	An alternative therapy concept to prednisolone for giant cell arteritis (GCA), considering the patient's age and comorbidities, could be:<br><br>Therapy Concept:<br>Initiate tocilizumab therapy.<br><br>Specific Drug Name and Dosage:<br>Tocilizumab: Administer 162 mg subcutaneously once weekly.<br><br>Rationale:<br><br>Tocilizumab, an interleukin-6 receptor inhibitor, is recommended as an alternative therapy for GCA in patients who have contraindications or intolerance to glucocorticoids, or are at high risk of glucocorticoid-related adverse effects, as per EULAR guidelines.<br>Given the patient's age and multiple comorbidities, including diabetes mellitus and hypertension, tocilizumab offers a steroid-sparing option with potentially fewer systemic side effects.<br>Subcutaneous administration facilitates ease of use and compliance for the elderly patient.<br>The dosage of 162 mg once weekly has been demonstrated to be effective in the treatment of GCA in clinical trials.<br>Regular monitoring for infections and liver function abnormalities is necessary during tocilizumab therapy.<br>Additionally, close follow-up assessments are required to evaluate treatment response and adjust the dosage or frequency as needed.	The alternative therapy suggestion of tocilizumab is based on its recommendation as an effective treatment option for giant cell arteritis (GCA) in patients who cannot tolerate or have contraindications to glucocorticoids, as outlined in EULAR guidelines. Tocilizumab offers a steroid-sparing approach with potential benefits in reducing glucocorticoid-related adverse effects, which is particularly important in an elderly patient with multiple comorbidities.

**GPT-4**	Based on the comprehensive patient profile provided, including the diagnosis of Giant Cell Arteritis (GCA) with no visual disturbances but significant systemic symptoms and elevated inflammatory markers, the most current European League Against Rheumatism (EULAR) guidelines for the management of GCA would recommend glucocorticoids (GCs) as the first-line treatment. Given the patient's age, comorbidities (aortic valve stenosis, diabetes mellitus type 2, arterial hypertension, dyslipidemia), and the need to minimize glucocorticoid-related side effects, a glucocorticoid-sparing agent should also be considered early in the treatment plan to reduce the glucocorticoid dose and associated toxicity. Tocilizumab, an interleukin-6 receptor alpha inhibitor, is recommended by EULAR guidelines as a glucocorticoid-sparing agent for patients with GCA, particularly those with relapsing disease or those at high risk of glucocorticoid-related adverse effects. Tocilizumab has been shown to be effective in maintaining remission and reducing the cumulative glucocorticoid dose.<br>Specific Therapy Suggestion:<br>Glucocorticoids: Start with Prednisolone (or equivalent glucocorticoid) at a dose adjusted for the patient's age, comorbid conditions, and the severity of GCA symptoms. A typical starting dose for GCA is about 0.5-1 mg/kg body weight per day. Given the patient's age and comorbidities, a lower end of the dosing range and careful monitoring would be prudent. For this patient (78 kg), an initial dose could be approximately 40 mg daily, with a plan to taper based on clinical response and reduction in inflammatory markers.<br>Tocilizumab: As a glucocorticoid-sparing agent, tocilizumab can be introduced early in the treatment regimen. The recommended dose for GCA is 162 mg administered subcutaneously once weekly. This treatment should be considered in consultation with the patient, considering potential benefits and risks, especially given the patient's age and comorbid conditions.<br>The therapy plan should include close monitoring for the efficacy and side effects of the treatment, with adjustments as needed. Regular follow-ups to monitor the patient's response to therapy, including symptoms, CRP/ESR levels, and potential side effects of the treatment, are crucial. Given the patient's diabetes, monitoring blood glucose levels is also important as glucocorticoids and tocilizumab can affect glycemic control.<br><br>It's essential to coordinate care with the patient's primary care provider and specialists managing his comorbid conditions, particularly in managing diabetes and hypertension, which may be affected by the proposed therapy.<br><br>Please note, this recommendation is based on the information provided and general guidelines. Individual patient care decisions should involve a multidisciplinary team and consider the patient's preferences, potential for side effects, and other factors unique to the patient's health status.	The recommendation for glucocorticoids as the initial treatment for Giant Cell Arteritis (GCA) is based on their effectiveness in quickly reducing inflammation and preventing complications, such as vision loss. Tocilizumab, an interleukin-6 receptor alpha inhibitor, is suggested as a glucocorticoid-sparing agent due to its proven ability to maintain remission in GCA, reduce the cumulative dose of glucocorticoids required, and consequently minimize the risk of glucocorticoid-related side effects, which is particularly important in elderly patients with comorbid conditions.	As an alternative therapy concept for the management of Giant Cell Arteritis (GCA) in a patient with significant comorbidities and for whom minimizing glucocorticoid exposure is a priority, Methotrexate can be considered as a glucocorticoid-sparing agent. Methotrexate has been used in GCA to reduce the total dose of glucocorticoids needed and to help in maintaining remission. While it may not be as effective as Tocilizumab for all patients, it offers a cost-effective alternative with a well-known safety profile, especially useful in settings where advanced biologic treatments might not be readily available or suitable.<br><br>Specific Alternative Therapy Suggestion:<br><br>Methotrexate: Start with a low dose, typically 7.5 to 15 mg once weekly, and adjust based on clinical response and side effects. Given the patient's age and comorbidities, starting at the lower end of this range (e.g., 7.5 mg weekly) and gradually increasing as tolerated and as necessary could be prudent.<br>Folic acid supplementation (e.g., 5 mg per week on a day different from the Methotrexate dose) is recommended to reduce the risk of side effects such as gastrointestinal upset and liver enzyme elevations.<br>Methotrexate serves as an immunosuppressant by reducing the activity of the immune system, which is beneficial in treating autoimmune conditions like GCA. It's chosen as an alternative to Tocilizumab for its effectiveness in reducing the need for long-term high-dose glucocorticoids, thereby lowering the risk of glucocorticoid-induced side effects. This recommendation considers the balance between efficacy, safety, patient comorbidities, and potential accessibility issues with biologic treatments.	Methotrexate is recommended as an alternative glucocorticoid-sparing agent in the management of Giant Cell Arteritis (GCA) due to its demonstrated efficacy in reducing the required dose of glucocorticoids and helping maintain disease remission, which is crucial for patients at high risk of glucocorticoid-related side effects. Its long history of use in various rheumatic diseases provides a well-characterized safety profile and experience in managing potential side effects, making it a suitable option for patients with multiple comorbidities.
