# Supplementary material for: Vignette-based comparative analysis of ChatGPT and specialist treatment decisions for rheumatic patients: results of the Rheum2Guide study
Source: Rheumatol Int. 2024 Aug 10;44(10):2043–53. doi: 10.1007/s00296-024-05675-5 (PMC11392980; doi:10.1007/s00296-024-05675-5)
Supplement: Supplementary file 2 — Supplementary file2 (DOCX 32 KB) [file 296_2024_5675_MOESM2_ESM.docx]

**Supplementary table 1**

| Case Vignette Number | Disease | Summary of clinical findings | Potential pitfalls | Vignette difficulty  (Median) |
| --- | --- | --- | --- | --- |
| 1 | RA | late-onset disease (81 years); high disease activity (DAS-28 5.46); disease duration 3 months; no previous RA therapies | reduced kidney function | 2 |
| 2 | RA | low to moderate disease activity (DAS-28 2.82); two swollen joints, disease duration 2 years | increased liver values when taking MTX and NSAIDs | 2 |
| 3 | RA | high disease activity (DAS-28 5.3); disease duration 10 years; various previous therapies (TNFi, IL-6Ri) | deep vein thrombosis and pulmonary artery embolism; carcinoma of the left breast in medical history | 3 |
| 4 | RA | moderate disease activity (DAS-28 4.8); disease duration 8 months; no previous RA therapies | active desire to have children | 2 |
| 5 | RA | moderate disease activity (DAS-28 3.17); disease duration 25 years; so far only cDMARDs have been taken | extra-articular manifestation: ulcerative keratitis | 4 |
| 6 | RA | high disease activity (DAS-28 5.2), disease duration 13 years; so far only cDMARDs have been taken | extra-articular manifestation: interstitial lung disease | 3 |
| 7 | axSpA | high disease activity (ASDAS-CRP 3.1); disease duration 8 months; insufficient response to two different NSAIDs | latent tuberculosis | 3 |
| 8 | PsA | high disease activity (DAPSA 20); moderate skin disease activity (PASI score 5.5); disease duration 24 months, no previous PsA therapy | active axial involvement; no peripheral joint involvement | 2 |
| 9 | axSpA | very high disease activity (ASDAS-CRP 3.8); disease duration 6 months; axial and peripheral joint involvement; no previous axSpA therapy | gastrointestinal ulcer occurred when taking NSAIDs regularly | 3 |
| 10 | PsA | high disease activity (DAPSA 18); monoarthritis of the right knee joint; low PASI score (0.6); no axial involvement; disease duration 12 years; previous therapies with cDMARDs and ustekinumab | ustekinumab underdosed depending on weight | 2 |
| 11 | PsA | very high disease activity (DAPSA 29); high PASI score; disease duration 35 years | complex, refractory disease; treatment failure of many different cDMARDs and bDMARDs | 4 |
| 12 | SLE | low disease activity (SLEDAI: 4); disease duration 11 years; no previous SLE therapy | secondary antiphospholipid syndrome, post pelvic vein thrombosis; anticoagulation with apixaban | 3 |
| 13 | SLE | active disease (SLEDAI: 11), double-stranded DNA antibodies highly elevated above the detection limit; disease duration 5 months | polyserositis and active lupus nephritis class III | 3 |
| 14 | SLE | active disease (SLEDAI: 9); arthralgia; splenomegaly; disease duration 6 years | autoimmune hemolytic anemia despite therapy with MMF and belimumab, hydroxychloroquine and low-dose prednisolone | 4 |
| 15 | dcSSc | rapidly progressive skin sclerosis, pulmonary and myocardial involvement; disease duration 1 year; previous therapy with MTX | rapidly progressive disease despite therapy with MMF | 4 |
| 16 | pSS | high disease activity (ESSDAI: 14); sicca symptoms; arthralgias; disease duration 5 years, current therapy: hydroxychloroquine | cryoglobulinemic vasculitis | 4 |
| 17 | ANCA-associated vasculitis (eGPA) | eosinophilia; typical pulmonary involvement; CNS vasculitis; possible renal involvement; disease duration unclear | multiple organ manifestations | 3 |
| 18 | ANCA-associated vasculitis (most likely GPA) | sinonasal involvement; disease duration 3 years, no DMARDs taken so far | unusual ANCA status (double positivitiy for MPO- and PR3 antibodies); condition after hepatitis b virus infection | 3 |
| 19 | ANCA-associated vasculitis (GPA) | Significantly elevated PR3-ANCA titer; disease duration 2 years; no previous therapies for ANCA-associated vasculitis | life-threatening condition; ventilation in intensive care due to pulmonary involvement | 3 |
| 20 | GCA | significantly elevated inflammatory parameters; headaches, disease onset 7 months ago at the age of 81 years | Aortitis; temporal arteritis; no ischemic complications so far; PMR overlap | 3 |

**Supplementary table 2**

| Case  difficulty level | Safety | Guideline  adherence | Medical  adequacy | Quality | Logic of  justification |
| --- | --- | --- | --- | --- | --- |
| RB | P=0.0368  R=-0.4695 | P=0.5512  R=-01417 | P=0.8170  R=0.05526 | P=0.6087  R=0.1219 | P>0.999  R=0.000 |
| GPT-3.5 | P=0.6196  R=-0.1182 | P=0.4199  R=0.1910 | P=0.5359  R=0.1471 | P=0.7068  R=-0.0897 | P=0.9620  R=0.0114 |
| GPT-4 | P=0.6434  R=-0.1103 | P=0.2431  R=0.2736 | P=0.4376  R=1840 | P=0.2368  R=0.2771 | P=0.9432  R=-0.0170 |

**Supplementary table 3**

| **Treatment plan** |  | **Safety** | **Conformity** | **Adequacy** | **Completeness** | **Quality** | **Logic of the Justification** |
| --- | --- | --- | --- | --- | --- | --- | --- |
|  |  | ICC (*95% CI*) | | | | | |
| **First-Line** | **RB** | 0.03 *(-0.02 to 0.15)* | 0 *(-0.07 to 0.13)* | 0.02 *(-0.05 to 0.18)* | 0.04 *(-0.04 to 0.19)* | -0.02 *(-0.08 to 0.1)* | -0.05 *(-0.11 to 0.07)* |
|  | **GPT-3.5** | 0.49 *(0.26 to 0.72)* | 0.34 *(0.13 to 0.59)* | 0.42 *(0.2 to 0.66)* | 0.2 *(0 to 0.47)* | 0.19 *(0.02 to 0.44)* | 0.13 *(-0.02 to 0.37)* |
|  | **GPT-4** | 0.31 *(0.11 to 0.58)* | 0.41 *(0.19 to 0.65)* | 0.41 *(0.19 to 0.65)* | 0.18 *(-0.01 to 0.46)* | 0.22 *(0.04 to 0.48)* | 0.1 *(-0.04 to 0.34)* |
| **Second-Line** | **RB** | 0.03 *(-0.02 to 0.14)* | 0.09 (-0.02 to 0.29) | -0.02 *(-0.0.05 to 0.07)* | -0.01 *(-0.05 to 0.09)* | -0.07 *(-0.1 to 0.05)* | -0.03 *(-0.08 to 0.1)* |
|  | **GPT-3.5** | 0.49 *(0.26 to 0.71)* | 0.48 (0.26 to 0.71) | 0.47 *(0.25 to 0.7)* | 0.23 *(0.02 to 0.5)* | 0.34 *(0.13 to 0.6)* | 0.38 *(0.16 to 0.63)* |
|  | **GPT-4** | 0.28 *(0.08 to 0.54)* | 0.53 *(0.32 to 0.74)* | 0.45 *(0.23 to 0.69)* | 0.15 *(-0.04 to 0.42)* | 0.31 *(0.1 to 0.56)* | 0.3 *(0.09 to 0.56)* |

**Supplementary table 1.** Overview of the case vignettes

A summary of clinical findings, characteristics/potential pitfalls and the complexity level (median of RB and raters) is shown in tabular form. Vignette difficulty varied from 1 (very easy) to 5 (very difficult).

*Abbreviations: RB, rheumatology board; RA, rheumatoid arthritis; DAS-28, disease activity score 28; NSAIDs, non-steroidal anti-inflammatory drugs; MTX, methotrexate; TNFi, tumor necrosis factor inhibitors; IL-6Ri, interleukin-6 receptor inhibitors; cDMARDs, conventional disease-modifying antirheumatic drugs; axSpA, axial spondyloarthritis; ASDAS-CRP, ankylosing spondylitis disease activity score; PsA, psoriatic arthritis; DAPSA, disease activity index for psoriatic arthritis; PASI, psoriatic area and severity index; SLE, systemic Lupus Erythematosus; SLEDAI, systemic lupus erythematosus disease activity index; MMF, mycophenolate mofetil; dcSSc, diffuse cutaneous systemic sclerosis; pSS, primary sjögren's syndrome; ESSDAI, EULAR sjögren's syndrome disease activity index; eGPA, eosinophilic granulomatosis with polyangiitis; ANCA, anti-neutrophil cytoplasmic antibody; MPO, myeloperoxidase; PR3, proteinase 3; GCA, giant cell arteritis; PMR, polymyalgia rheumatica.*

**Supplementary table 2.** Correlation analysis of median case difficulty and ratings.

Significant correlations. *Abbreviations: RB, rheumatology board.*

**Supplementary table 3.** Inter-rater agreement.

*Abbreviations: ICC, intra-class correlation coefficient; CI, confidence interval.*
